# Targeting FOXM1 regulates metabolic signatures through ROS-dependent JNK/Bmi1/Skp2 axis in human cutaneous T-cell lymphoma

**DOI:** 10.1038/s41419-025-08389-z

**Published:** 2026-01-07

**Authors:** Abdul Q. Khan, Maha Agha, Fareed Ahmad, Rasheeda Anver, Majid Alam, Joerg Buddenkotte, Shahab Uddin, Martin Steinhoff

**Affiliations:** 1 Translational Research Institute, Academic Health System, Hamad Medical Corporation, Doha, Qatar; 2 Dermatology Institute, Academic Health System, Hamad Medical Corporation, Doha, 3050 Qatar; 3 Department of Dermatology and Venereology, Rumailah Hospital, Hamad Medical Corporation, Doha, 3050 Qatar; 4 Department of Medicine, Weill Cornell Medicine Qatar, Qatar Foundation-Education City, Doha, 24144 Qatar; 5 Department of Medicine, Weill Cornell Medicine, 1300 York Avenue, New York, NY 10065 USA; 6 College of Medicine, Qatar University, Doha, 2713 Qatar

**Keywords:** Cancer metabolism, Skin cancer

## Abstract

Cutaneous T-cell lymphoma (CTCL) is a progressive and heterogeneous malignancy characterized by deregulated metabolic reprogramming and cancer stemness, with limited therapeutic options. Therefore, elucidating the mechanisms driving metabolic reprogramming and poor clinical outcomes in CTCL is imperative. Forkhead box protein M1 (FOXM1), an oncogenic transcription factor, plays a pivotal role in cancer pathogenesis by orchestrating metabolic reprogramming and stemness signaling, thereby contributing to therapeutic resistance. In this study, we investigated the therapeutic potential of FOXM1 inhibition in human CTCL cells. Both genetic and pharmacological targeting of FOXM1 markedly suppressed CTCL cell growth and proliferation by inducing programmed cell death (apoptosis and autophagy) via reactive oxygen species (ROS) generation. Mechanistic analyses revealed that the activation of the MAPK, particularly JNK activation, is crucial for thiostrepton-induced programmed cell death. Metabolomics profiling further demonstrated that thiostrepton treatment triggers ROS- and JNK-dependent alteration in metabolic pathways central to cancer hallmarks, including amino acid and lipid metabolism. Notably, FOXM1 inhibition abrogated stemness-associated metabolic reprogramming genes (KLF-4, Bmi1) and Skp2, while upregulating the tumor suppressor p21 in a JNK-dependent manner. Moreover, thiostrepton treatment sensitized the CTCL cells to proteasome inhibitor bortezomib, promoting apoptosis and autophagy. Collectively, these findings demonstrate that FOXM1 targeting disrupts the metabolic status and stemness features of CTCL cells via JNK activation, thereby offering novel insights into potential therapeutic strategies for overcoming therapeutic challenges in CTCL.

## Facts

- FOXM1 is integral to the acquisition of various cancer hallmarks.
- FOXM1 modulates metabolic signatures in CTCL cells.
- Inhibition of FOXM1 enhances the efficacy of bortezomib in CTCL cells.
- ROS-dependent JNK activation is crucial for FOXM1 depletion-induced cell death.

## Introduction

Cancer is a complex and heterogeneous disorder often accompanied by metabolic dysregulation and aberrant signaling that drives disease progression and associated challenges [1]. Cutaneous T-cell lymphoma (CTCL) is an alarming cancer of lymphoid organs characterized by a significant increase in malignant T cells in the skin [2]. CTCL represents a heterogeneous group of T-cell disorders or non-Hodgkin lymphomas of the skin (Mycosis Fungoides and Sézary syndrome), and can also metastasize to distant parts of the body [3]. Indeed, the deregulated proliferation of skin-resident CD4+ T cells is a characteristic feature in both Mycosis Fungoides and Sézary syndrome, accounting for more than 50% of all CTCLs [4]. It is also a major cause of recalcitrant chronic skin rash that mimics other dermatological problems, leading to significant diagnostic delay [3]. Recent epidemiological reports further suggest an increasing incidence, particularly among the young population in the United States [5].

Increasing evidence suggests that CTCL pathogenesis is not only driven by the genetic heterogeneity but also by recurrent genetic and epigenetic alterations, and deregulated oncogenic signaling [6]. These abnormalities lead to a poor prognosis for advanced-stage CTCL patients, whose response to therapeutics is often very poor and short-lived [7, 8]. As in many other malignancies, deregulated signaling cascades-particularly MAPK, NF-κB, and JAK/STAT-are central to CTCL progression, driving hallmark features such as uncontrolled proliferation, metabolic reprogramming, therapeutic resistance, and recurrence [6–8]. Consequently, it is essential to identify signaling mechanisms of the CTCL pathogenesis that can serve as novel therapeutic targets.

Deregulated signaling has been the prime factor associated with the development of cancer hallmarks, including drug resistance and recurrence with poor survival. In line, MAPK signaling is extensively studied in chronic human diseases, including hematological malignancies [9–11]. Thus, identifying aberrant signaling pathways is imperative to attenuate the hallmarks of CTCL pathogenesis. Deregulated transcription factors (TFs) are critical in several types of human cancer pathogenesis and associated complications. Similarly, CTCL cancer patients have shown deregulated expression of various TFs, including FOXM1, NF-KB, STAT, and TWIST [12–17], which contribute to its heterogeneity and recurrent alterations.

Forkhead box protein M1 (FOXM1), a member of the FBOX family of TFs, is particularly critical in regulating genes involved in proliferation, metabolism, and stemness [18–24]. Although the oncogenic role of FOXM1 is well-established in solid tumors and B-cell lymphomas [19, 25], its function in CTCL has been relatively less explored. This represents a significant gap, particularly since deregulation of FOXM1 has been associated with poor outcomes in related hematological malignancies.

Given the pathogenic significance of FOXM1, considerable efforts have been made to develop or identify effective FOXM1 inhibitors. Among them, thiostrepton (TST), an FDA-approved natural cyclic oligopeptide antibiotic and inhibitor of protein translation produced by Streptomyces, has emerged as a potent FOXM1 inhibitor with anticancer, antimicrobial, and anti-inflammatory activities [26–29]. Mechanistically, TST blocks the binding or interaction of the forkhead or DNA-binding domain of FOXM1 with the regulatory regions of different genes, thereby suppressing the transcription of oncogenic target genes [29–31]. Despite these advances, the effects of FOXM1 inhibition on CTCL pathogenesis remain unexplored.

Combination targeted therapies have become a cornerstone in CTCL management to improve survival and reduce patient burden [7, 32]. Building on this concept, FOXM1 inhibition may enhance the efficacy of proteasome inhibitors such as bortezomib (BOR), providing a promising avenue for therapeutic intervention.

In this study, we investigated the mechanisms underlying the anticancer effects of FOXM1 inhibition in CTCL using both pharmacological (TST) and genetic (siRNA) approaches. Our results show that targeting FOXM1 suppresses cellular proliferation, induces cell-cycle arrest, and activates programmed cell death pathways, including apoptosis and autophagy. We further demonstrate that ROS-induced JNK activation is integral for TST-mediated anti-CTCL actions and abrogation of cancer stemness. Metabolomic profiling identifies FOXM1 as a key regulator of metabolic reprogramming and energy production, reinforcing its role in CTCL progression and adverse clinical outcomes. Finally, we show that FOXM1 inhibition enhances CTCL cell sensitivity to bortezomib, highlighting its potential as a therapeutic target in novel combination treatment strategies.

## Chemicals and reagents

CCK-8, dimethyl sulfoxide (DMSO), 3-methyladenine, thiostrepton, monodansylcadaverine (MDC), and other high-grade reagents were purchased from Sigma-Aldrich (St. Louis, Missouri, United States). VAD (Z-VAD-FMK (carbobenzoxy-valyl-alanyl-aspartyl-[O-methyl]- fluoromethylketone) was purchased from Selleck Chemicals (14408 W Sylvanfield Drive, Houston, TX 77014 USA). Different antibodies, including Phospho-JNK (Thr183/Tyr185) (Cat# 4668), JNK (Cat# 9252), FOXM1 (Cat# 20459), Caspase-3 (Cat# 9662), Cleaved caspase-3 (Cat# 9661), Cleaved caspase-8 (Cat# 9496), Skp2 (Cat# 4358), Bmi1(Cat# 6964), p21 (Cat# 64016), PARP (Cat# 9542), Cleaved PARP (Cat# 9544), LC3A/B (Cat# 4108), GAPDH (Cat# 5174), and β-actin (Cat# 4967), were procured from Cell Signaling Technology, Inc. (3 Trask Lane Danvers, MA 01923). KLF4 (Cat# SC-166190), p-H2AX (Cat# SC-517348), and HSP60 (Cat# SC13115) were purchased from Santa Cruz Biotechnology, Inc. (Finnell Street, Dallas, Texas, USA). Bortezomib and SP600125 were purchased from Cell Signaling Technology, Inc., USA. Laemmli Sample buffer 1X, resolving Gel Buffer, acrylamide/Bis solution, stacking gel solution, developer kit (Clarity Western ECL) purchased from BIO-RAD (Hercules, California, USA).

### Cell culture

Human cutaneous T-cell lymphoma cell lines (HH and H9) were obtained from the American Type Culture Collection (ATCC; Manassas, VA, USA). Each cell line was accompanied by a Certificate of Analysis confirming authentication by short tandem repeat (STR) profiling and verification of absence of mycoplasma contamination. Upon receipt, frozen vials were thawed and cultured in Roswell Park Memorial Institute (RPMI) 1640 Medium supplemented with 10% (v/v) fetal bovine serum (FBS), 100U/ml penicillin, and 100U/ml streptomycin at 37 °C in a humidified atmosphere containing 5% CO2. Equal numbers of cells were plated into culture dishes or 96-well plates and randomly assigned to treatment groups (control, drug/inhibitor, or inhibitor + drug) using a simple randomization method to ensure unbiased allocation and equal representation across groups.

### Cell viability assay

The effect of various treatments, including TST, bortezomib, N-acetyl cysteine (NAC), Z-VAD-FMK, and SP600125, either alone or in combination, on the proliferation and survival of CTCL cell lines (HH and H9) was assessed using the Cell Counting Kit-8 (CCK-8) assay, as described earlier [33]. Briefly, 1 × 10^4^ cells were seeded into 96-well plates and treated with the indicated drugs/inhibitors, followed by incubation at 37 °C in a humidified atmosphere containing 5% CO_2._ Subsequently, 10 µl of CCK-8 reagent was added to each well and incubated for the recommended period. Absorbance was then measured at 450 nm using a microplate reader.

### Annexin V/propidium iodide dual staining

To assess apoptosis and necrosis, HH and H9 cells were treated with TST, bortezomib, N-acetyl cysteine (NAC), z-VAD-FMK, SP600125, either alone or in combination. The cell fractions were determined using flow cytometry, as described previously [34]. Briefly, cells were gently washed with PBS and then stained with fluorescein-conjugated annexin-V antibody and propidium iodide for 30 min at room temperature in annexin binding buffer. Stained cells were then analyzed using a BD LSRFortessa flow cytometer (BD Biosciences, USA). The frequencies of apoptotic and necrotic cells were quantified using FlowJo software (version 10.7.1).

### Measurement of mitochondrial membrane potential

Mitochondrial membrane potential (MMP) alterations of HH and H9 cells treated with TST were evaluated using the JC-1 stain kit. JC-1 is a cationic, lipophilic dye that accumulates in mitochondria in a potential-dependent manner, with the red/green fluorescence ratio serving as an indicator of MMP. A decrease in this ratio indicates a loss of mitochondrial membrane potential. Cells were washed with PBS, stained with JC-1 according to the manufacturer’s instructions, and analyzed using flow cytometry (BD LSR Fortessa analyzer, BD Biosciences, USA) as previously described [33, 35]. Data were processed using FlowJo software (version 10.7.1).

### Live/dead assay

HH and H9 cells were treated with TST, N-acetyl cysteine (NAC), and SP600125, either alone or in combination, and then washed with PBS. Cells were stained using LIVE/DEAD Viability/Cytotoxicity Kit (cat# L3224, Thermo Fisher) according to the manufacturer’s instructions. Briefly, a staining solution containing calcein-AM and ethidium homodimer-1 (EthD-1) was prepared and added to the cells, followed by incubation at room temperature for 15–30 minutes in the dark. Live and dead cells were identified by green fluorescence (calcein-AM), and red fluorescence (EthD-1), respectively. Stained cells were visualized, and images were captured using the EVOS FLc cell imaging system from Invitrogen (Thermo Fisher Scientific) as previously described [33].

### Cell lysis and immunoblotting

To further investigate the mechanisms underlying programmed cell death and cell signaling, HH and H9 cells were treated with TST, bortezomib, N-acetyl cysteine (NAC), Z-VAD-FMK, and SP600125, either alone or in combination. After treatment, the cells were harvested, washed with PBS, and lysed to measure protein concentration. An equal amount of proteins from each sample was resolved using SDS-PAGE and transferred onto polyvinylidene difluoride (PVDF) membranes. The membranes were blocked with milk and then incubated with primary antibodies overnight at 4 °C. Following this, the membranes were washed and probed with secondary antibodies. Finally, the protein bands were visualized using enhanced chemiluminescence (ECL) on a Chemi-Doc System (Bio-Rad, Hercules, California, USA) [33].

### Cell cycle analysis

The effect of TST on the cell-cycle distribution of CTCL cell lines (HH and H9) was assessed as previously described [34]. Following treatment, cells were harvested, fixed in ethanol, and stored overnight at 4 °C. The next day, cells were washed with HBSS and incubated in PI/RNase staining solution (Cat# 550825, BD Biosciences) for 15 min at room temperature. After staining, cells were washed, resuspended in HBSS, and analyzed using a BD LSRFortessa flow cytometer (BD Biosciences, USA). Cell-cycle profiles were quantified using FlowJo software (version 10.7.1).

### Analysis of cell autophagy

Cellular autophagy was assessed by flow cytometry, using monodansylcadaverine (MDC) staining as described previously [36]. Briefly, HH and H9 cells were treated with TST, followed by incubation with 50 µM MDC, a fluorescent dye that selectively accumulates in autophagic vacuoles. After stating, cells were washed with PBS, centrifuged, and the resulting cell pellets were resuspended in PBS containing propidium Iodide (PI) to exclude dead cells. Samples were incubated in the dark to prevent photobleaching and subsequently analyzed using a BD LSRFortessa flow cytometer. Autophagy levels were quantified based on the intensity of MDC fluorescence, and data analysis was performed using FlowJo software (version 10.7.1).

### FOXM1 siRNA transfection

FOXM1 expression was silenced via siRNA-mediated transfection. We sourced FOXM1 siRNA (catalogue no. S100421050, lot no. 100579824) and scrambled control siRNA (catalogue no. 1027281, lot no. 190563210) from Qiagen. Cells were transfected with Lipofectamine 2000 reagent (Invitrogen) according to the manufacturer’s protocol as described previously [37]. Briefly, siRNAs and Lipofectamine were each diluted in Opti-MEM medium and incubated for 10 min before being combined and incubated for an additional 30 min. The resulting complexes were then applied to the cells. After 6 h, the medium was replaced with complete growth medium, and the cells were maintained for 48 h at 37 °C in a humidified incubator with 5% CO₂. Transfected cells were then harvested, washed with PBS, lysed, and assessed for protein concentration. Western blotting was performed to determine FOXM1 knockdown efficiency, with scrambled siRNA included as a negative control for non-specific effects.

### CTCL spheroid culture

CTCL spheroids were generated following the protocol described previously [34], with minor modifications. Briefly, cells were initially cultured in 10% RPMI media and maintained under standard culture conditions. An equal number of cells along with drugs or inhibitors were then seeded into ultra-low attachment plates (Corning, USA) using complete cancer stem cell medium (3D Tumorsphere Medium XF, Promo Cell, Germany, C-28070). Cultures were maintained in a humidified chamber containing 5% CO_2_ at 37 °C. The size, morphology, and compactness of the spheroids were monitored regularly. At the end of the experimental period, spheroids were imaged using an EVOS FL imaging system from Invitrogen (Invitrogen, Thermo Fisher Scientific) at a magnification of 4× to assess structural integrity and potential treatment-induced alterations.

### Targeted metabolomics

Targeted metabolomics of TST, SP600125, and NAC-treated CTCL cells was done using MxP® Quant 500 kit (Biocrates Life Sciences AG, Innsbruck, Austria) with an ultra-High-Performance Liquid Chromatography (UPLC) system coupled to a Triple Quad 5500 + MS/MS (SCIEX, Framingham, MA, USA), as previously described [34]. Briefly, 10 μL cell lysate, calibration standards, and quality control samples were transferred onto a 96-well plate containing a filter with internal standards. The plate was dried under a stream of nitrogen gas and subsequently incubated with 5% phenyl-isothiocyanate to effectively derivatize the amino acids and biogenic amines. Dried analytes were then extracted using 5 mmol ammonium acetate, followed by dilution and LC-MS/MS analysis. Metabolite separation was achieved using the MxP® Quant 500 kit LC column system (Biocrates) with the mobile phase gradient of A: 0.2% formic acid in water and B: 0.2% formic acid in acetonitrile. All operational parameters were set as per the MxP® Quant 500 kit specifications. Data acquisition was carried out with Analyst software (SCIEX) and processed in MetIDQ software (Biocrates). Further analysis was done using the MetaboAnalyst 6.0 platform (https://www.metaboanalyst.ca/)[38]. Briefly, data were processed and normalized prior to analysis. Univariate (one-way ANOVA with FDR correction, p < 0.05; t-test, volcano plots) and multivariate analyses (PLS-DA) were performed. Significantly altered metabolites were identified using univariate methods. Metabolite set enrichment and pathway analysis were carried out using the SMPDB and KEGG databases.

### Statistical analysis

Data are presented as mean ± S.D. Statistical analyses were performed using GraphPad Prism version 10.0 (GraphPad Software Inc., San Diego, CA, USA). Comparisons among multiple experimental groups were conducted using one-way ANOVA followed by appropriate post hoc tests, while differences between two groups were analyzed using an unpaired Student’s *t* test. A p-value less than 0.05 was considered statistically significant (*p < 0.05). Higher levels of significance were indicated as *p* < 0.01(**), *p* < 0.001 (***), and *p* < 0.0001 (****). Each graph represents the mean of at least three replicates, with an error bar indicating the standard deviation.

## Results

### Thiostrepton inhibits the proliferation of CTCL cells

**Fig. 1:**
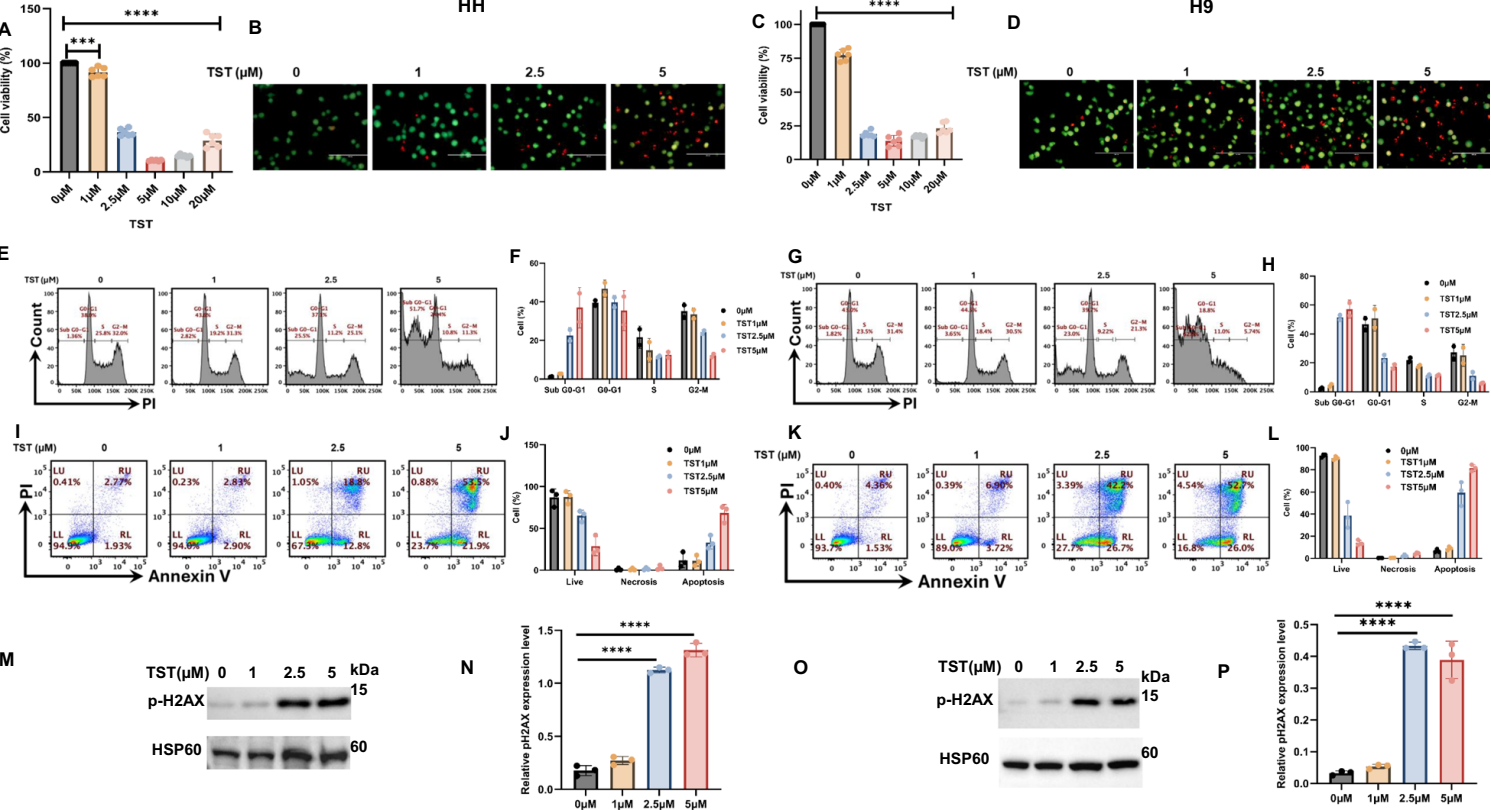
Thiostrepton (TST) inhibits the growth of CTCL cells. **A**, **C** HH and H9 cells were treated with increasing concentrations of TST for 24 hours, and the cell viability was measured using CCK-8. The data are presented as mean ± SD (*n* = 6). **B**, **D** Show representative images of live/dead cell staining, with green, fluorescent calcein-AM dye indicating intracellular esterase, and red-fluorescent ethidium homodimer-1 indicating loss of membrane integrity. **E**–**H** Representative flow cytometry-based cell cycle analysis of HH and H9 cells treated with TST for 24 h (*n* = 3). (**I**, **K**) shows the representative flow cytometric dot plot analysis of live, early apoptotic, late apoptotic, and necrotic cells treated with TST for 24 h, followed by staining with fluorescein-conjugated Annexin-V/PI. LL: live, LR: early apoptotic, UR: late apoptotic, UL: necrotic cells. (**J**, **L**) shows the quantification of apoptosis and necrosis, expressed as mean ± SD (*n* = 3). **M**–**P** Western blot analysis of p-H2AX and its densitometric quantification are presented as mean ± SD (*n* = 3). Band intensities were normalized with the respective loading control. *P < 0.05, and ****P < 0.0001 indicate the level of significance between treatment groups compared to the control.

Aberrant growth and proliferation of cancer cells are the primary concerns associated with cancer-related challenges, including the therapeutic drawbacks. In this line, we first treated HH and H9 cells with different concentrations of TST (0 µM, 1 µM, 2.5 µM, 5 µM, 10 µM, and 20 µM) for 24 hours and then used CCK-8 to determine cell viability. Interestingly, our results show that TST treatment significantly suppressed the proliferation of CTCL cells in a dose-dependent fashion (Fig. 1A and Fig. 1C). Moreover, live and dead cell staining analysis also revealed a marked increase in nuclear and membrane alterations in TST-treated CTCL cells, as illustrated in HH (Fig. 1B) and H9 (Fig. 1D) cells. Notably, siRNA-mediated FOXM1 knockdown significantly inhibits the proliferation of HH and H9 cells, suggesting a key role of FOXM1 in tumor cell growth and proliferation (Supplementary Fig. S1, A, B).

Furthermore, we performed cell cycle analysis on CTCL cells treated with TST, and the results showed increased cell population in sub-G0-G1 and G0-G1 arrest, as demonstrated by flow cytometry analysis (Fig. 1E–H). Similarly, we further analyzed the change in mitochondrial membrane potential (MMP) due to TST treatment in CTCL cells, and our results indeed show a marked decrease in MMP (Supplementary Fig. S2,A–D). This prompted us to further analyze the percentages of live, necrotic, and apoptotic cells following TST treatment. Consistently, TST significantly increased apoptotic and necrotic cells (Fig. 1I–L), thereby supporting its anti-proliferative potential.

### Thiostrepton inhibits the proliferation of CTCL cells through apoptosis and autophagy

**Fig. 2:**
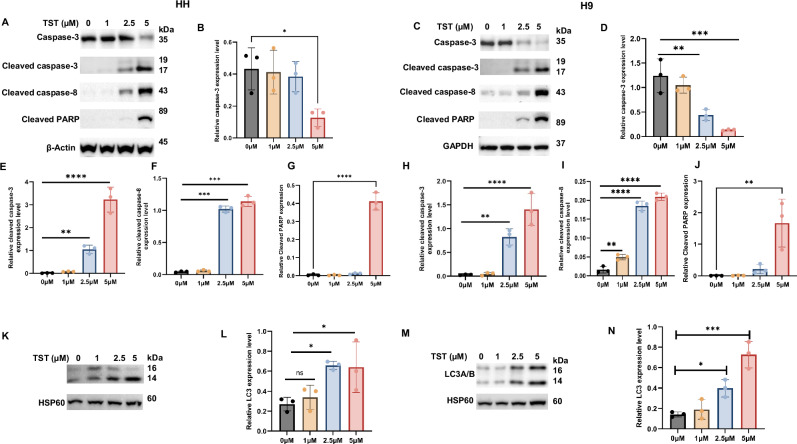
Thiostrepton inhibits the proliferation of CTCL cells by inducing programmed cell death. **A**–**J** HH and H9 cells were treated with the indicated concentrations of TST. Cell lysates were subjected to western blot analysis of caspase-3, cleaved caspase-3, cleaved caspase-8, and cleaved PARP. Relative quantification of band intensities is shown as mean ± SD (*n* = 3), normalized to the respective loading control. **K**–**N** Western blot analysis of LC3 in HH and H9 cells treated with the indicated concentrations of TST. Relative quantification of band intensities is presented as mean ± SD (*n* = 3), normalized to respective loading control. Statistical significance is indicated as *P < 0.05, **P < 0.01, ***P < 0.001, and ****P < 0.0001, representing the level of significance between treatment groups relative to the control.

**Fig. 3:**
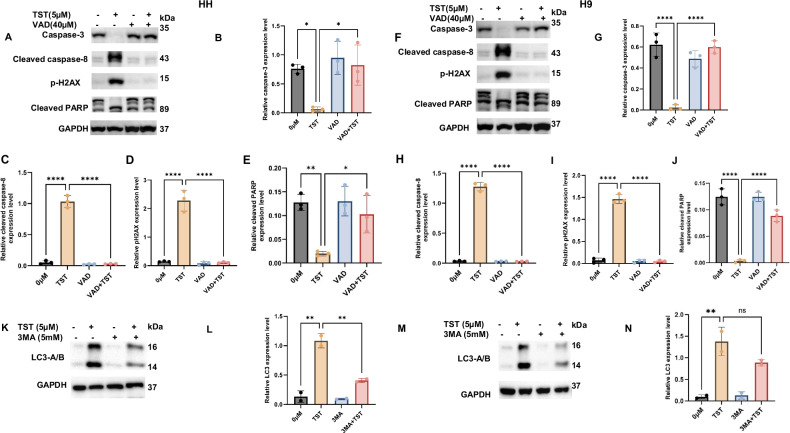
Thiostrepton inhibits the proliferation of CTCL cells by inducing programmed cell death. **A**–**J** HH and H9 cells were treated with the indicated concentrations of TST and Z-VAD-FMK, alone or in combination. Cell lysates were prepared, and western blot analysis of caspase-3, cleaved caspase-8, p-H2AX, and cleaved PARP was performed. Relative quantification of protein band is presented as mean ± SD (*n* = 3). The band intensities were normalized to the respective loading control. **K**–**N** HH and H9 cells were treated with the indicated concentrations of TST and 3-MA, either alone or in combination. Cell lysates were prepared, and western blot analysis of LC3 was performed. Relative quantification results of the LC3 expression are presented as mean ± SD (*n* = 3). Band intensity was normalized to the respective loading control. *P < 0.05, **P < 0.01, and ****P < 0.0001 represent the level of significance between treatment groups relative to control (positive and negative) groups.

**Fig. 4:**
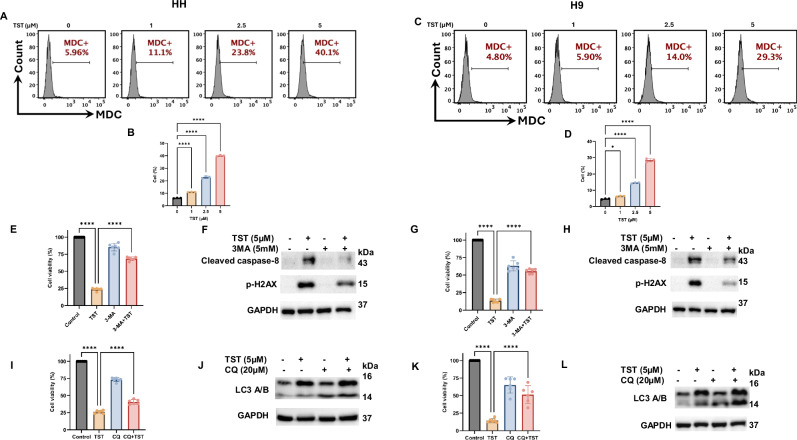
Thiostrepton induces autophagy in CTCL cells. **A**–**D** HH and H9 cells were treated with increasing concentrations of TST for 24 h. The percentage of MDC-positive cells was acquired and analyzed using flow cytometry, and the results are presented as mean ± SD (*n* = 3). **E**, **I**, **G**, **K** HH and H9 cells were treated with TST (5 μM), autophagy inhibitors chloroquine (CQ) (20 μM) or 3-methyladenine (3-MA) (5 mM), either alone or in combination, for 24 h. Cell viability was assessed using the CCK-8 assay (*n* = 6). **F**, **H**, **J**, **L** HH and H9 cells were treated with the indicated concentrations of TST, CQ, or 3-MA, alone or in combination, for 24 h. Cell lysates were prepared and analyzed by immunoblotting for cleaved caspase-8, LC3A/B, and p-H2AX. *P < 0.05, and ****P < 0.0001 represent the level of significance between treatment groups relative to control (positive and negative) groups.

To further investigate the mechanisms underlying TST-mediated growth inhibition of CTCL cells, we examined various proteins involved in the regulation of apoptosis and autophagy. First, we observed a significantly increased expression of DNA damage marker p-H2AX (Fig. 1M–P). Next, we explored the status of apoptosis and autophagy proteins, and our results show that TST activated various caspases, including caspase-3, cleaved caspase-3, cleaved caspase-8, DNA repair enzyme PARP, and LC3 (Fig. 2A–J). Additionally, siRNA-mediated inhibition of FOXM1 also triggered programmed cell death, as evidenced by changes in the expression of caspase-3, cleaved caspase-3, and p-H2AX (Supplementary Fig. S1). To support the role of apoptosis in TST-treated cells, we utilized z-VAD-FMK, a pan-caspase inhibitor. Notably, z-VAD-FMK reversed TST-induced cell viability inhibition (Supplementary Fig. S2E, F). Furthermore, as illustrated in Fig. 3A–J, z-VAD-FMK significantly reversed the TST-induced activation of caspases (caspase-3 and cleaved caspase-8) as well as cleaved PARP and p-H2AX. These data provide evidence for the crucial role of apoptosis in CTCL cells. We also investigated the role of autophagy, another form of cell death, in TST-treated CTCL cells. Initially, we assessed autophagy induction through flow cytometry using MDC staining and observed a significant increase in MDC-positive cells in TST-treated HH and H9 cells (Fig. 4A–D). Additionally, our data revealed a significant increase in LC3 expression level in HH and H9 cells, as depicted in Fig. 2K–N. Consistently, siRNA-mediated FOXM1 targeting also modulated expression of LC3A/B and P62, which are integral in autophagy (Supplementary Fig. S1,C). Moreover, 3-Methyladenine (3MA), an early-stage autophagy inhibitor, markedly suppressed the expression of LC3, further supporting the role of autophagy in TST-induced growth inhibition of CTCL cells (Fig. 3K–N). Importantly, CQ, an inhibitor of late-stage autophagy, enhanced the accumulation of LC3A/B in TST-treated cells, as shown in Fig. 4J, L. These results indicate disruption in the formation of the autophagosome and autophagolysosome. Further, we observed that the autophagy inhibitors CQ and 3-MA reversed TST-induced growth inhibition in CTCL cells (Fig. 4 E, I G, K). Notably, TST-induced apoptosis was reversed by 3-MA as demonstrated by the expression of cleaved caspase-8 and p-H2AX (Fig. 4F, H). Overall, these findings suggest that inhibition of autophagy diminishes the cytotoxic effect of TST in CTCL cells.

### Reactive oxygen species (ROS) are integral in thiostrepton-induced apoptosis and autophagy in CTCL cells

**Fig. 5:**
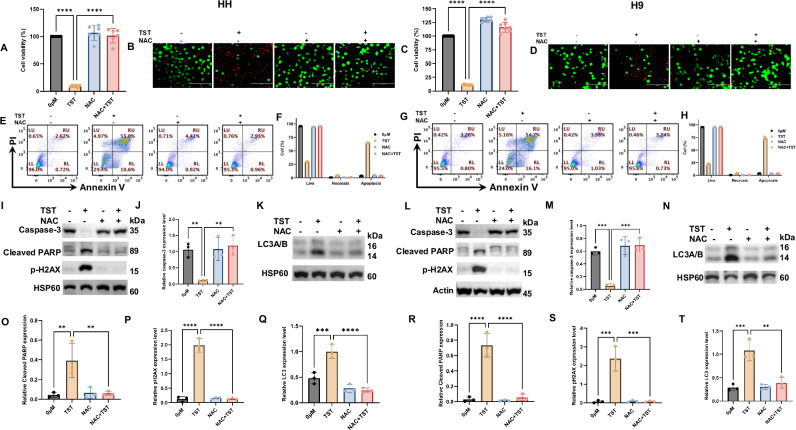
Thiostrepton inhibits the growth of CTCL cells through ROS generation. **A**, **C** HH and H9 cells were treated with TST (5 μM) and N-Acetyl-cystine (NAC) (6 mM), alone or in combination, and cell viability was evaluated using the CCK-8 assay. Results are presented as mean ± SD (*n* = 6). **B**, **D** shows the representative live/dead cell staining in HH and H9 cells treated with TST (5 μM) and NAC (6 mM), alone or in combination. Green fluorescence indicates (calcein-AM) live cells, whereas red fluorescence (ethidium homodimer-1) denotes dead cells. **E**, **G** shows the representative flow cytometric dot plot analysis of live, early apoptotic, late apoptotic, and necrotic cells treated with TST (5 μM) and NAC (6 mM), alone and or in combination, after staining with fluorescein-conjugated Annexin-V/PI. LL: live, LR: early apoptotic, UR: late apoptotic, UL: necrotic cells. **F**, **H** shows the percentage quantification of live, apoptotic, and necrotic cells of treatment groups in HH and H9 cells (*n* = 3). **I**–**T** Western blot analysis of apoptotic and autophagy markers in HH and H9 cells treated with TST (5 μM) and NAC (6 mM) either alone or in combination. Relative quantification of protein band intensities are presented as mean ± SD (*n* = 3). The intensity of the bands was normalized with the respective loading control. **P < 0.01, ***P < 0.001, and ****P < 0.0001 represent the level of significance between treatment groups relative to control (positive and negative) groups.

The role of reactive oxygen species (ROS) is indispensable in maintaining biological homeostasis, as well as in diverse types of cancer therapy, including chemotherapy, radiotherapy, and phototherapy. A disturbed redox status triggers various cellular events, including programmed cell death. Therefore, we aimed to explore the potential role of ROS in TST-induced growth inhibition and programmed cell death in CTCL cells. Our findings show that treatment of CTCL cells with N-acetyl cysteine (NAC) reversed TST-induced reduction in cell viability, underscoring the important role of ROS in TST-mediated growth inhibition (Fig. 5A, C). Live and dead cell staining corroborated these results, showing a reversal in TST-induced dead cell staining with NAC treatment (Fig. 5B, D). To further confirm the role of ROS in TST-induced programmed cell death, we demonstrated that NAC treatment significantly reversed TST-mediated annexin-PI staining, as illustrated in Fig. 5E–H. Additionally, we examined the expression of various apoptosis-related proteins (caspase-3, cleaved PARP, and p-H2AX) and an autophagy-related protein (LC3) in cells treated with NAC and TST, both alone and in combination. We observed a significant change in the expression of these proteins (Fig. 5I-T). Overall, these findings collectively support the crucial role of ROS in TST-induced programmed cell death.

### ROS-dependent JNK activation is vital in TST-induced programmed cell death

**Fig. 6:**
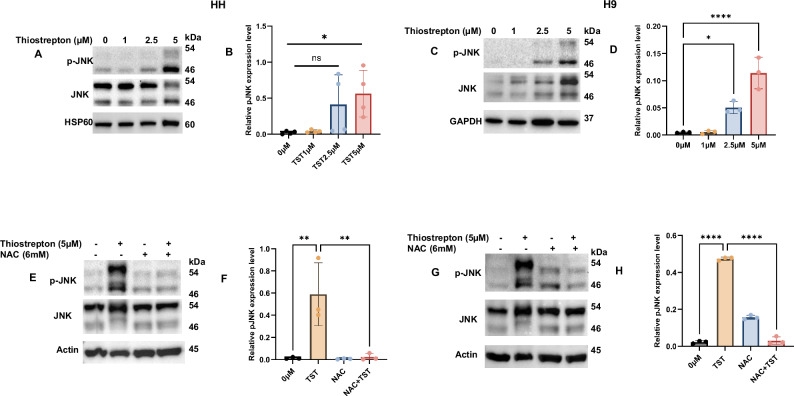
Thiostrepton activates MPAK signaling through ROS generation. **A**–**D** HH and H9 cells were treated with the indicated concentration of TST for 24 h, and cell lysates were prepared. Immunoblotting was performed, and blot analysis of p-JNK, JNK, and their relative quantification results are presented as mean ± SD (*n* = 3). The intensity of the bands was normalized with the respective loading control and quantified using image lab software. **E**–**H** HH and H9 cells were treated with the indicated concentrations of TST and NAC, either alone or in combination, and cell lysates were prepared. Western blot analysis of p-PJNK, JNK, and their relative quantification results are presented as mean ± SD (*n* = 3). The intensity of the bands was normalized with the respective loading control and quantified using Image Lab software. *P < 0.05, **P < 0.01, and ****P < 0.0001 represent the level of significance between treatment groups relative to control. ns (non-significant).

Mitogen-activated protein kinase (MAPK) signaling is integral in regulating the underlying mechanisms of biological and physiological homeostasis. Increasing evidence highlights MAPK as one of the central regulators of growth, proliferation, stemness, therapy, and drug resistance. Hence, we first checked the status of MAPK signaling proteins in TST-treated cells. Strikingly, our results showed significant activation of p-JNK in CTCL cells, as depicted in Fig. 6A–D. Considering the crucial role of ROS in programmed cell death, we also explored the role of ROS in TST-induced JNK activation in HH and H9 cells. Intriguingly, our data shows a significant (*p* < 0.05) inhibition of TST-mediated JNK activation using NAC, and hence evidently supports the role of TST-induced ROS generation in JNK activation (Fig. 6E–H). Thus, these results provide evidence about the importance of ROS-triggered MAPK signaling in TST-treated CTCL cells.

### TST causes growth inhibition and programmed cell death via JNK activation

**Fig. 7:**
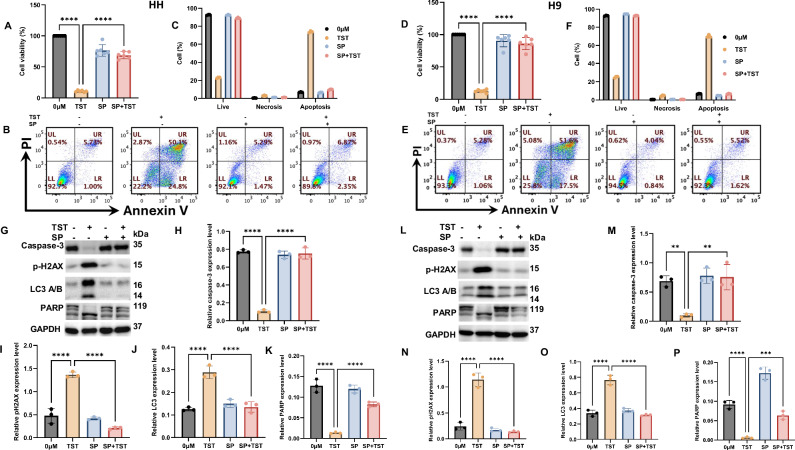
TST induces growth inhibition and programmed cell death in CTCL cells through JNK activation. **A**, **D** HH and H9 cells were treated with TST(5 μM) and SP600125 (10 μM), alone or in combination. Cell viability was determined by using a CCK-8 kit assay. Results are presented as mean ± SD (*n* = 6). **B**, **E** shows the representative flow cytometric dot plot analysis of the live, early apoptotic, late apoptotic, and necrotic cells treated with TST and SP600125, alone or in combination, followed by staining with fluorescein-conjugated Annexin-V/PI. LL: live, LR: early apoptotic, UR: late apoptotic and necrotic, UL: necrotic cells. **C**, **F** shows the percentage of apoptosis and necrosis in CTCL cells treated with TST and SP600125 alone and in combination (*n* = 3). **G**–**P** HH and H9 cells were treated with TST(5 μM) and SP600125 (10 μM), alone or in combination. Western blot analysis of caspase-3, p-H2AX, LC3, and PARP. Relative quantification data of band intensities is presented as mean ± SD (*n* = 3), normalized with the respective loading control and quantified using Image Lab software. **P < 0.01, ***P < 0.001, and ****P < 0.0001 represent the level of significance between treatment groups relative to control (positive and negative) groups.

c-Jun N-terminal kinase (JNK) signaling regulates numerous essential cellular processes, including apoptosis, autophagy, survival, proliferation, metabolic reprogramming, and cancer stemness features. Cancer therapies often act through modulation of JNK, but the exact mechanism remains unclear. In this context, we investigated the crucial role of JNK in the antiproliferative effects of TST and the underlying mechanisms in CTCL cells using SP600125, a specific JNK inhibitor. Our initial findings revealed a significant (*p* < 0.05) reversal of TST-induced reduced viability in CTCL cells, highlighting the essential role of JNK signaling (Fig. 7A, D). Additionally, we performed flow cytometry-based annexin staining and observed a significant (*p* < 0.05) reduction in the number of cells undergoing programmed cell death due to JNK inhibition in TST-treated cells (Fig. 7 B, C, E, F). This further supports the critical role of JNK in TST-mediated programmed cell death. Motivated by these results, we assessed expression levels of regulatory proteins involved in programmed cell death using western blotting in cells treated with TST alone and in combination with SP600125. Notably, we observed a significant change in the expression of caspase 3, p-H2AX, LC3, and PARP due to JNK inhibition. This indicates the vital role of JNK activation in TST-induced growth inhibition of CTCL cells (Fig. 7G–P). Collectively, these findings demonstrate the integral role of JNK signaling in anti-CTCL effects of TST.

### JNK signaling is important for TST-mediated regulation of proteins associated with cancer Hallmarks

**Fig. 8:**
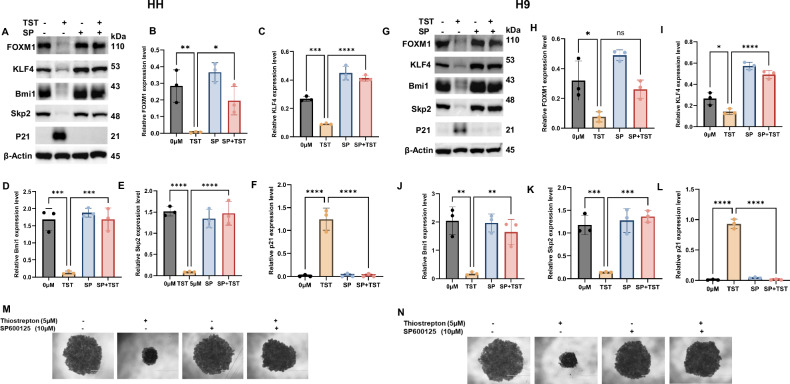
TST inhibits the expression of regulatory proteins of metabolic reprogramming through JNK upregulation. HH and H9 cells were treated with TST(5 μM) and SP600125 (10 μM), either alone or in combination. Following treatment, cell lysates were prepared, and western blotting was performed. **A**–**L** show the immunoblotting results for FOXM1, KLF4, Bmi1, Skp2, and p21, along with their densitometric quantification data presented as mean ± SD (*n* = 3). Band intensities were normalized to the respective loading control and quantified using Image Lab software. Statistical significance is indicated as *P < 0.05, **P < 0.01, ***P < 0.001, and ****P < 0.0001 compared with the respective control (positive and negative) groups. **M**, **N** Demonstrate that JNK inhibition reversed TST-mediated suppression of spheroid formation in HH and H9 cells.

Aberrant expression and the function of regulatory proteins play a crucial role in the orchestration of cancer hallmarks, including stemness, metabolic reprogramming, and acquired resistance. To investigate this, we examined the status of FOXM1, KLF4, Bmi1, Skp2, and p21, as well as their correlation with JNK status in CTCL cells treated with TST. Our data indicate that JNK inhibition significantly reversed the expression of FOXM1, KLF4, Bmi1, Skp2, and p21 in TST-treated CTCL cells (Fig. 8A–L). Given that TST acts as a pharmacological FOXM1 inhibitor, we also explored the potential correlation between siRNA-mediated FOXM1 gene silencing and the expression levels of FOXM1, Bmi1, and Skp2. We found a significant (*p* < 0.05) inhibition in the expression of these proteins in the FOXM1 knocked down CTCL cells (Supplementary Fig. S1C). Additionally, we assessed the effect of JNK inhibition on the 3D-spheroid of TST-treated CTCL cells using an ultralow attachment 96-well plate. Notably, our results showed that JNK inhibition markedly attenuated and restored the spheroid structure as compared to the TST-only-treated cells (Fig. 8M, N). Taken together, these findings suggest that TST mediates tumor suppressive effects, including the attenuation of regulatory proteins associated with cancer hallmarks through JNK signaling.

### TST modulates metabolic profiling of CTCL cells through JNK-dependent FOXM1 inhibition

**Fig. 9:**
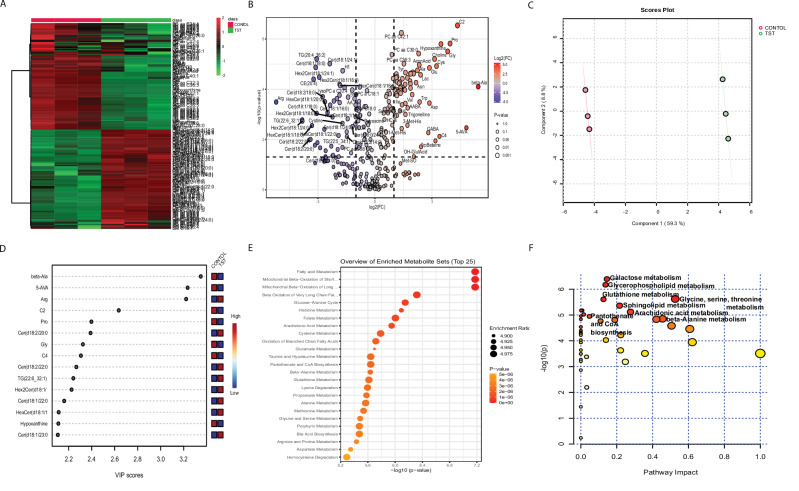
Metabolomic profiling of CTCL cells (HH) treated with TST (5 μM). **A** Heatmap with hierarchical clustering of the significantly altered metabolites (*p* < 0.05) in TST-treated CTCL cells. Red indicates increased metabolite levels and green indicates decreased levels, with samples shown in columns and metabolites in rows. **B** Volcano plot of the differentially expressed metabolites. The metabolites represented by red dots are up-regulated, whereas metabolites shown as blue dots are down-regulated. Fold change and the raw p-value cutoffs were set at 2.0 and 0.05, respectively. **C** PLS-DA of metabolomics data from control and TST tread samples. **D** Variable importance in projection (VIP) score plot of the top 15 features based on the PLS-SDA model. The color scale on the right side (red and blue) indicates increased and decreased levels in the control and TST groups, respectively. **E** Functional analysis of the significant features in TST-treated cells using MetaboAnalyst 6.0 (https://www.metaboanalyst.ca/). Quantitative enrichment analysis (QEA) overview showing the top 25 enriched metabolic pathways based on SMPDB. **F** Pathway analysis based on the KEGG database. Each circle represents a pathway, where circle color and size correspond to the *p*-value (enrichment analysis) and pathways’ impact (topology analysis), respectively.

We performed the LC-MS-based targeted metabolomics profiling of CTCL cells treated with TST, SP600125, and NAC, either alone or in combination, using the MxP Quant 500 kit from Biocrates, as outlined in the Materials and Methods section. The data were normalized and statistically analyzed (univariate and multivariate) using Web-based Metaboanalyst V. 6.0. One-way ANOVA with post hoc analysis identified 205 metabolites/features with a significant change in CTCL cells treated with TST, SP600125, and NAC, either alone or in combination. The robustness of our findings is further supported by the detailed statistical characteristics, including f-values, p-values, and FDR of the significant features, presented in Supplementary Table S1. We also show the relative abundance of key metabolites through heatmap and box plot analysis in CTCL cells treated with TST, SP600125, and NAC, alone or in combination (Supplementary Fig. S3). One-way ANOVA with post hoc analysis revealed significant changes in the relative abundance of metabolites from various biochemical classes, including amino acids and amino acid–related metabolites, carboxylic acids, biogenic amines, free fatty acids, vitamins/cofactors, nucleobases, alkaloids, and amine oxides, acylcarnitines, ceramides, hexosylceramides, lysophosphatidylcholines, phosphatidylcholines, and sphingomyelins, in CTCL cells treated with TST, SP600125, and NAC, either alone or in combination (Supplementary Fig. S3,A–K). Overall, treatment with TST resulted in substantial alterations in metabolite profiles in CTCL cells, suggesting that TST drastically altered the metabolic fingerprints essential for the growth and survival of CTCL cells. Next, we performed the metabolomics data analysis of CTCL cells treated with TST compared to the controls. A t-test reveals a significant change in 184 features (p < 0.05) (Supplementary Table S2). Heatmap with hierarchical clustering analysis of the significant features (p < 0.05) demonstrated a distinct metabolite alteration in TST-treated cells, with red indicating increased and green depicting decreased metabolite levels (Fig. 9A). Moreover, volcano plot analysis using fold change (FC = 2) and raw p-value (0.05), identified 50 downregulated (blue dots), and 63 upregulated (red dots) metabolites (Fig. 9B, Supplementary Table S3). Furthermore, comparative metabolomics analysis of TST vs TST + SP600125 and TST vs NAC + TST treatments showed modulation of the TST-induced metabolic changes as depicted by the heat maps (Supplementary Figs. S4,A, S5,A), and volcano plots (Supplementary Figs. S4,B, S5,B, and Supplementary Tables S4, S5).

Inline, chemometric Partial Least Squares Discriminant Analysis (PLS-DA) also showed a clear separation between control and TST-treated groups, with a variance of 59.3% for component 1 and 8.8% for component 2 (Fig. 9C), supporting a clear metabolomics-based differential abundance of metabolites. These findings suggest that the different metabolites or the different metabolic signatures play a role in each treatment group. Additionally, we conducted a variable importance in projection (VIP) score analysis, which identified key signatures/metabolites driving these TST-induced metabolic changes (Fig. 9D). PLS-DA also showed a clear separation between the different treatment groups: TST vs. TST + SP600125, and TST vs. NAC + TST, as illustrated in Supplementary Figs. S4,C and S5,C, respectively. Furthermore, the VIP scores highlighting critical metabolites in these treatment groups are also shown in Supplementary Figs. S4,D and S5,D, respectively. Collectively, these data demonstrate that FOXM1 targeting alters the metabolic state of cancer cells, contributing to the attenuation of cancer hallmarks.

### TST-mediated FOXM1 inhibition targets metabolic pathways in CTCL

To assess the effect of TST on metabolic pathways, we performed metabolite set enrichment analysis using the Small Molecule Pathway Database (SMPDB) with a quantitative enrichment approach of the significant features. This analysis highlighted the top 25 enriched metabolite sets in TST-treated cells (Fig. 9E; Supplementary Table S6). In line, pathway analysis of the significant features using the KEGG database revealed that TST treatment influenced several critical metabolic pathways, including galactose metabolism, glycerophospholipid metabolism, primary bile acid biosynthesis, lipoic acid metabolism, glutathione metabolism, arachidonic acid metabolism, glycine/serine/threonine metabolism, pantothenate and CoA biosynthesis, and beta-alanine metabolism (Fig. 9F; Supplementary Table S7). The statistical values for these pathways, including *p*-value, –log(p), Holm-adjusted p-value, FDR, and pathway impact, are provided in Supplementary Table S7.

Furthermore, metabolite set enrichment and pathway analyses of TST vs. TST + SP600125 (Supplementary Fig. S4,E, F; Supplementary Tables S8, S9) and TST vs. NAC + TST treatment groups (Supplementary Fig. S5,E, F; Supplementary Tables S10, S11) based on SMPDB and KEGG databases demonstrated a modulation in the TST-induced metabolic alterations. Overall, metabolomics profiling revealed that TST treatment profoundly reprogrammed the metabolic landscape of CTCL cells, altering the levels of amino acids, lipids, carboxylic acids, biogenic amines, and related metabolites. Pathway enrichment analysis identified significant changes in glycerophospholipid, glutathione, cysteine/methionine, and other key metabolic pathways. Notably, inhibition with SP600125 or NAC reversed many of these alterations, underscoring the role of ROS-JNK signaling. These findings demonstrate that FOXM1 targeting by TST disrupts central metabolic pathways, contributing to the attenuation of cancer hallmarks.

### TST treatment sensitized CTCL cells to bortezomib (Velcade)

**Fig. 10:**
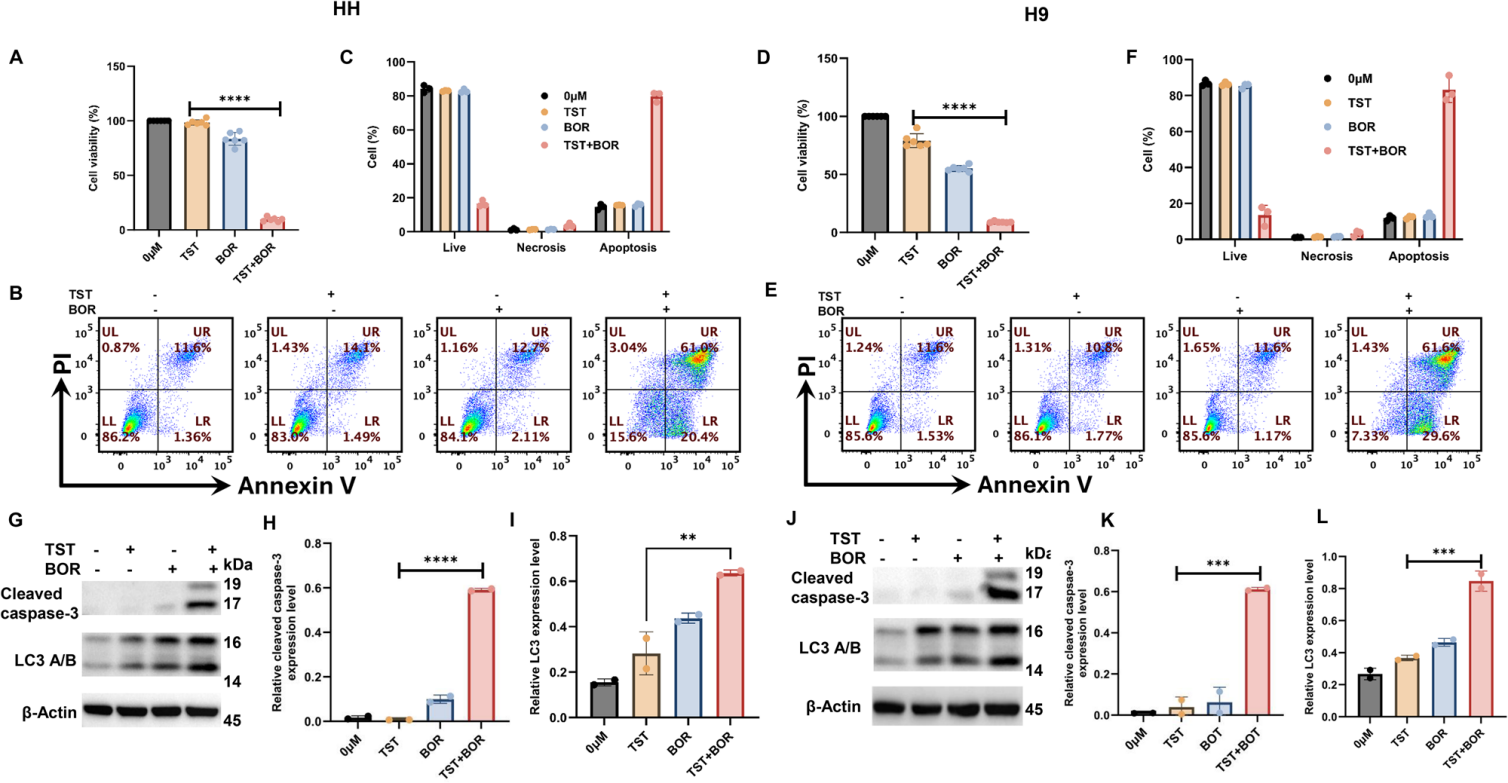
TST sensitizes CTCL cells to bortezomib (BOR). HH and H9 cells were treated with TST (1 µM) and BOR (5 nM), either alone or in combination, for 24 h. **A**, **D** show the effects of TST and BOR, alone or in combination, on the percentage of cell viability in HH and H9 cells. The results are expressed as mean ± SD (*n* = 6). **B**, **E** Representative flow cytometry dot plot analysis of live, early apoptotic, late apoptotic, and necrotic cells treated with TST and BOR, alone or in combination, for 24 h, followed by staining with fluorescein-conjugated Annexin-V/PI. LL: live, LR: early apoptotic, UR: late apoptotic, UL: necrotic cells. **C**, **F** Displays the percentage of live, apoptosis, and necrosis (*n* = 3). **G**–**L** Western blot analysis of cleaved caspase-3 and LC3 in HH and H9 cells following the indicated treatments. Relative quantification of band intensities is presented as mean ± SD. Band intensities were normalized with the respective loading controls and quantified using Image Lab software. **P < 0.01, ***P < 0.001, and ****P < 0.0001 indicate the levels of significance between treatment groups.

There is a growing concern about how to enhance the therapeutic outcomes of anticancer therapeutics. In this context, we explored the sensitizing potential of TST in CTCL cells to the anticancer drug bortezomib. Our initial results demonstrated a significant decrease in cell viability in cells treated with TST + bortezomib compared to either treatment alone (Fig. 10A, D). Consistently, flow cytometry–based Annexin V/PI staining revealed a significant increase in the percentage of apoptotic cells following combined treatment (Fig. 10 B, C, E, F). Furthermore, we observed a noticeable increase in the expression of cleaved caspase-3 and LC3 in cells treated with TST + bortezomib compared to their counterparts alone (Fig. 10G–L). Thus, these findings suggest that FOXM1 inhibition can potentiate the sensitivity of cancer cells to anticancer therapies.

## Discussion

Cutaneous T-cell lymphoma is a progressive and heterogeneous malignancy characterized by the abnormal accumulation of malignant T cells in the skin, leading to significant distress for patients and their families. Despite advances in CTCL research, the available therapeutic options are primarily symptomatic, highlighting the need to investigate the underlying mechanisms contributing to clinical challenges. In this study, we demonstrate for the first time the crucial role of FOXM1 in CTCL growth, proliferation, and the metabolic regulation required for maintaining cancer stemness. Our findings show that TST exerts potent anticancer effects and suppresses cancer stemness features through the activation of tumor suppressors via ROS-dependent JNK activation. Furthermore, metabolomics analyses underscore the central role of FOXM1 in modulating cellular metabolism and metabolites implicated in CTCL pathogenesis.

Thiostrepton, the bacterium *Streptomyces azureus*, was first used as an antibiotic in 1954. Since then, its use as a potential therapeutic agent has increased significantly, and it is now an FDA-approved antibiotic. Various findings suggest that TST specifically inhibits the nuclear binding of the transcription factor FOXM1 [30]. Interestingly, this inhibition of FOXM1 by TST has been shown to suppress the pathogenesis of human malignancies and the features of cancer stemness, including therapy resistance [21, 27, 28, 39]. Furthermore, recent studies have highlighted the potential of TST to sensitize cancer cells to conventional therapeutic measures [26, 40]. Given TST’s therapeutic values, we potentially for the first time have explored its therapeutic importance in managing CTCL. Our results demonstrate that TST inhibits the growth and proliferation of CTCL cells by blocking the nuclear binding of FOXM1. We also investigated underlying mechanisms associated with TST-induced growth inhibition in CTCL cells. TST treatment increased both the subG0-G1 population and G0-G1 cell cycle arrest. It also upregulated p21, a well-established cyclin-dependent kinase inhibitor that inhibits the activity of CDK2 and CDK4, thereby preventing the activation of the retinoblastoma protein (Rb) and the subsequent transcription of genes required for S-phase entry. As a result, cells fail to progress from G1 to S phase, leading to G0–G1 arrest [41]. This establishes a mechanistic link between TST exposure and growth inhibition through cell cycle arrest. Furthermore, annexin staining, MMP analysis, and the expression profiles of key regulatory proteins collectively demonstrate the involvement of both apoptosis and autophagy in TST-induced cell death.

As reactive oxygen species play a significant role in TST-mediated pharmacological effects, this study also examined the ROS level in TST-mediated growth inhibition of CTCL cells [28]. Our findings highlight the essential role of ROS in TST-induced growth inhibition, apoptosis, and autophagy in these cells, which are supported by previous studies [28, 42]. Our findings further demonstrate the central role of the MAPK (JNK) in TST-mediated growth inhibition of CTCL cells. Furthermore, our results highlight that the JNK activation plays a central role in TST-induced growth inhibition, apoptosis, and autophagy, thereby underscoring its importance as an underlying mechanism.

Tumor cell stemness plays a crucial role in cancer recurrence and can lead to poor clinical outcomes [43, 44]. We investigated the effect of TST on the regulators of the stemness features, and our results indicate that it leads to the downregulation of stemness regulatory genes in a JNK-dependent manner. Skp2, an F-box protein and known oncogene, plays a vital role in pathogenesis and stemness of various human malignancies, including CTCL, by targeting cyclin-dependent kinase inhibitors (CDKIs) such as p21 [45–48]. Notably, FOXM1 is among the transcription factors reported to regulate SKP2 expression [48, 49]. Our findings provide compelling evidence that deregulation of the FOXM1/Skp2/p21 axis contributes to the pathogenesis of CTCL. Furthermore, we demonstrate that TST-induced JNK activation is critical for suppressing CTCL pathogenesis and stemness through inhibiting this axis, in agreement with previous reports [29]. Additionally, TST-mediated modulation of GKLF/KLF4 and Bmi1 expression via JNK activation underscores its role in regulating cancer stemness features.

Krüppel-like Factor 4 (KLF4/GKLF), a zinc finger transcription factor, plays an important role in developmental and stemness, functioning as both a transcriptional activator and suppressor. Its deregulated expression is frequently associated with the stemness features in cancer cells [50, 51]. In line, we also observed modulation of B-cell-specific Moloney murine leukemia virus integration region 1 (Bmi-1), a polycomb group (PcG) of protein that plays a central role in cancer stemness and pathogenesis [52]. Our findings reveal that TST suppresses Bmi1 through JNK activation, in agreement with previous studies showing that Bmi1 inhibition abrogates cancer stemness [53, 54]. Therefore, these results highlight that targeting FOXM1 disturbs key pathways, thereby abrogating cancer stemness in CTCL cells.

Metabolic deregulation or reprogramming plays an imperative role in cancer pathogenesis and is strongly associated with poor clinical outcomes [1, 55]. Aberrant activation of FOXM1 has been implicated in metabolic reprogramming and maintaining stemness in various human malignancies [56, 57]. However, the metabolic consequences of FOXM1 inhibition in cutaneous T-cell lymphoma (CTCL) remain largely unexplored. In this context, metabolomics profiling of CTCL cells provides valuable insights into the underlying mechanisms in TST-mediated anticancer effects, particularly highlighting how JNK-dependent FOXM1 suppression perturbs key metabolic pathways and metabolites involved in tumor growth and survival. Consistent with this, statistical analysis revealed significant alterations in the relative abundance of metabolites across different treatments, confirming distinct treatment-specific metabolite signatures. Pathway enrichment analysis demonstrated TST-induced perturbation in multiple cancer-relevant pathways, including galactose metabolism, glycerophospholipid metabolism, primary bile acid biosynthesis, lipoic acid metabolism, glutathione metabolism, arachidonic acid metabolism, glycine/serine/threonine metabolism, pantothenate and CoA biosynthesis, and beta-alanine metabolism. These metabolic pathways are crucial for sustaining the growth, proliferation, and stemness of cancer cells, and thus alteration in these pathways provides strong evidence that TST drives programmed cell death through metabolic stress and JNK activation [58–62]. Collectively, these findings highlight the crucial role of metabolic reprogramming in CTCL and elucidate how TST reprograms CTCL metabolism to induce JNK-mediated cell death, thereby revealing metabolic vulnerabilities that can be targeted for novel therapeutic strategies.

Interestingly, there has been an increasing focus on developing combinational therapeutics to enhance clinical outcomes for cancer patients. In this context, our findings strongly support the therapeutic importance of TST, both as a standalone treatment and in combination with other agents. Notably, we observed that TST, at subtoxic concentrations, sensitizes CTCL cells to the FDA-approved drug bortezomib, consistent with previous reports [29, 63–65]. These findings not only strengthen the rationale for combinational approaches but also highlight TST as a promising candidate for integration into CTCL treatment strategies.

In summary, this study delineates a novel therapeutic mechanism in which TST targets FOXM1-driven pathways, reprograms cellular metabolism, and induces programmed cell death via JNK activation. Furthermore, TST sensitizes CTCL cells to the proteasome inhibitor, highlighting a potential synergistic effect. These findings reveal important metabolic and stemness-associated vulnerabilities in CTCL, underscoring the potential of TST as a promising candidate for therapeutic development, either as monotherapy or in strategic combinations.

## Supplementary information

Western blots

Supplementary Figures

Supplementary Tables

## Data Availability

The data sets generated and/ or analyzed during this study are available from the corresponding author on reasonable request.
