# Supplementary material for: Targeting FOXM1 regulates metabolic signatures through ROS-dependent JNK/Bmi1/Skp2 axis in human cutaneous T-cell lymphoma
Source: Cell Death Dis. 2026 Jan 7;17(1):170. doi: 10.1038/s41419-025-08389-z (PMC12876963; doi:10.1038/s41419-025-08389-z)
Supplement: Supplementary file 1 — Western blots [file 41419_2025_8389_MOESM1_ESM.pptx]

## Slide 1
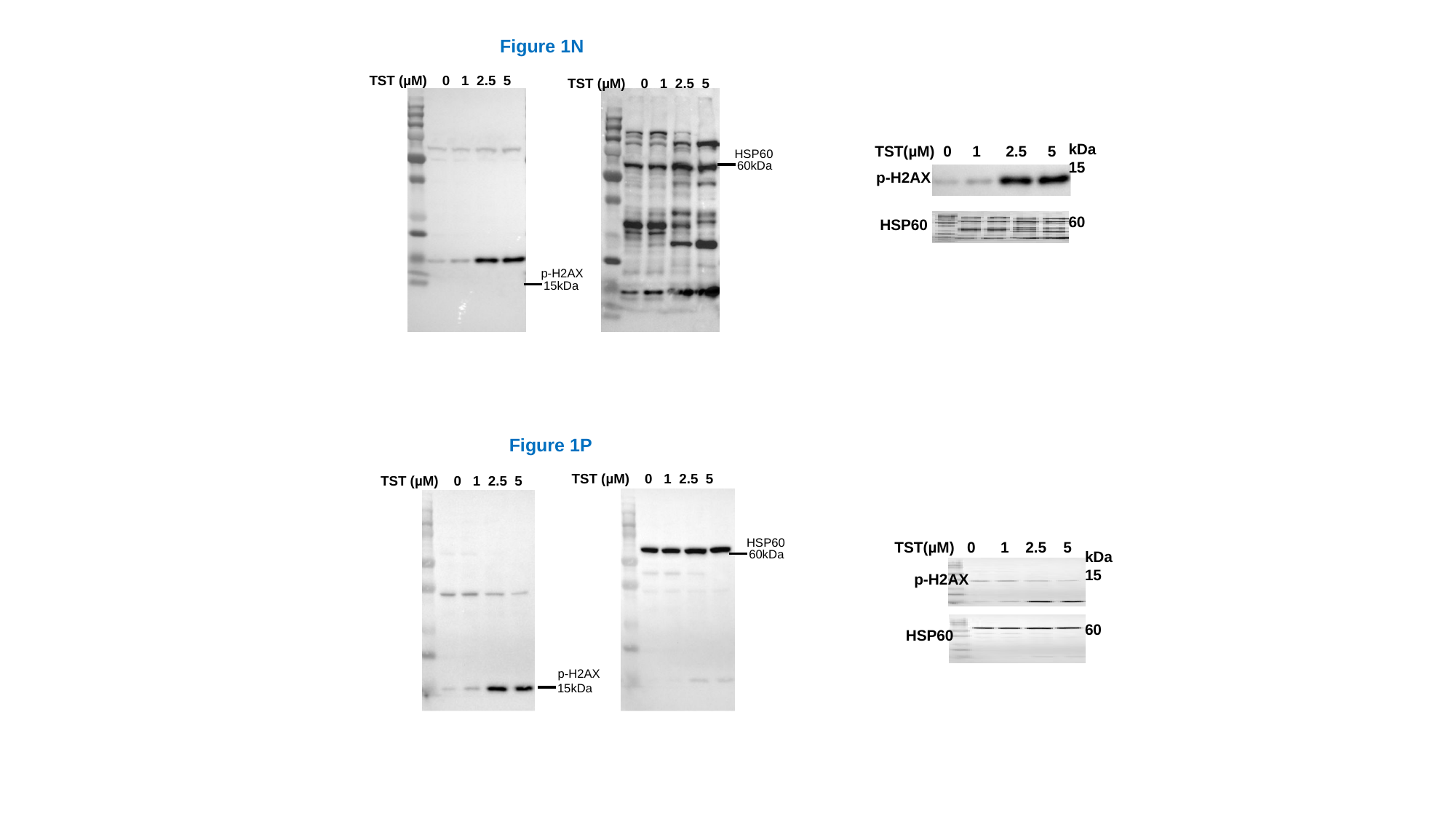

Figure 1N
TST (µM) 0 1 2.5 5
p-H2AX
15kDa
TST (µM) 0 1 2.5 5
HSP60
60kDa
kDa
15
60
TST(µM) 0 1 2.5 5
p-H2AX
HSP60
Figure 1P
TST (µM) 0 1 2.5 5
HSP60
60kDa
TST (µM) 0 1 2.5 5
p-H2AX
15kDa
TST(µM) 0 1 2.5 5
kDa
15
60
p-H2AX
HSP60

## Slide 2
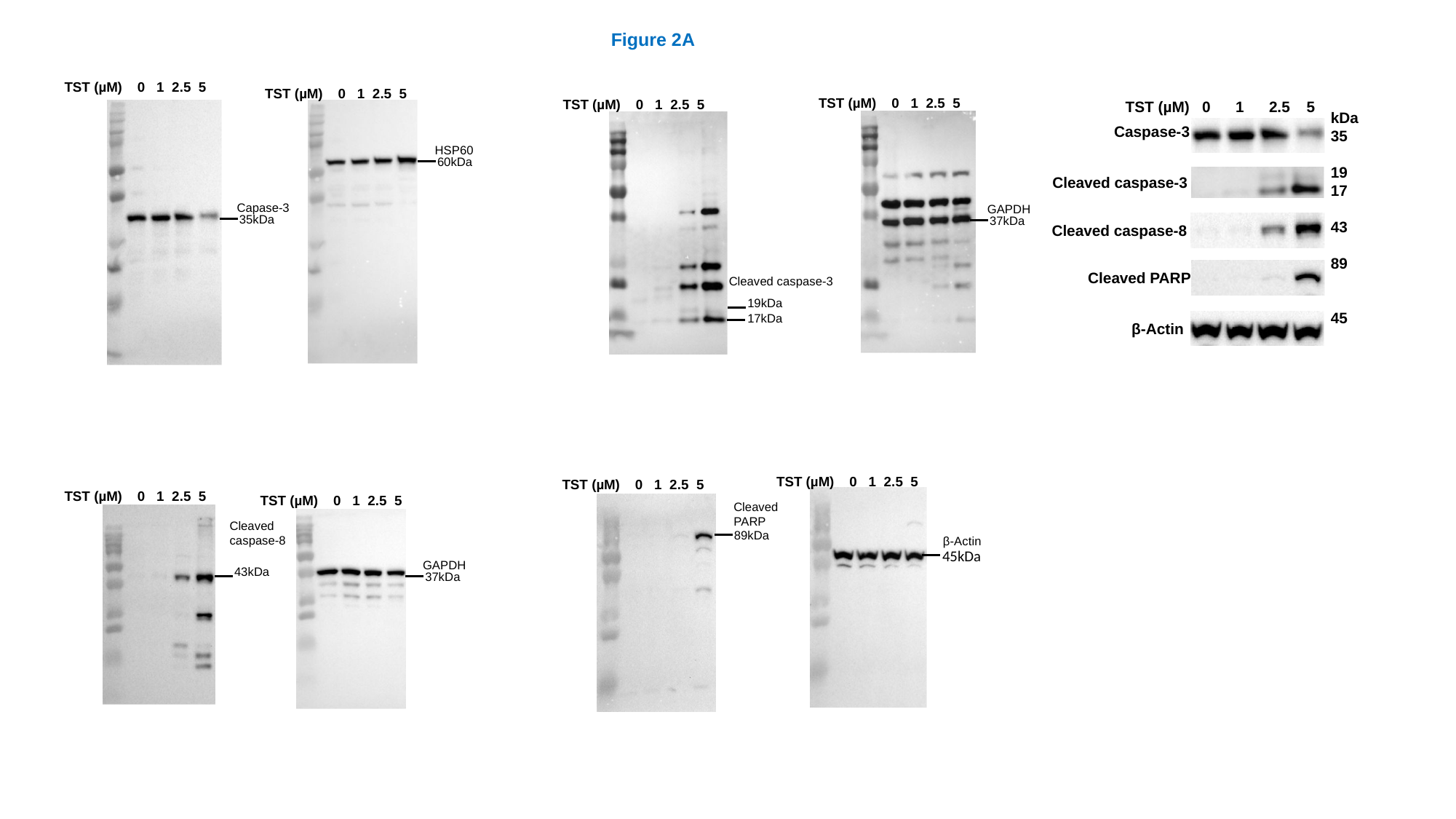

Figure 2A
TST (µM) 0 1 2.5 5
Capase-3
35kDa
TST (µM) 0 1 2.5 5
HSP60
60kDa
TST (µM) 0 1 2.5 5
GAPDH
37kDa
TST (µM) 0 1 2.5 5
Cleaved caspase-3
19kDa
17kDa
TST (µM) 0 1 2.5 5
kDa
35
19
17
43
89
45
Caspase-3
Cleaved caspase-3
Cleaved caspase-8
Cleaved PARP
β-Actin
TST (µM) 0 1 2.5 5
β-Actin
45kDa
TST (µM) 0 1 2.5 5
Cleaved PARP
89kDa
TST (µM) 0 1 2.5 5
Cleaved caspase-8
43kDa
TST (µM) 0 1 2.5 5
GAPDH
37kDa

## Slide 3
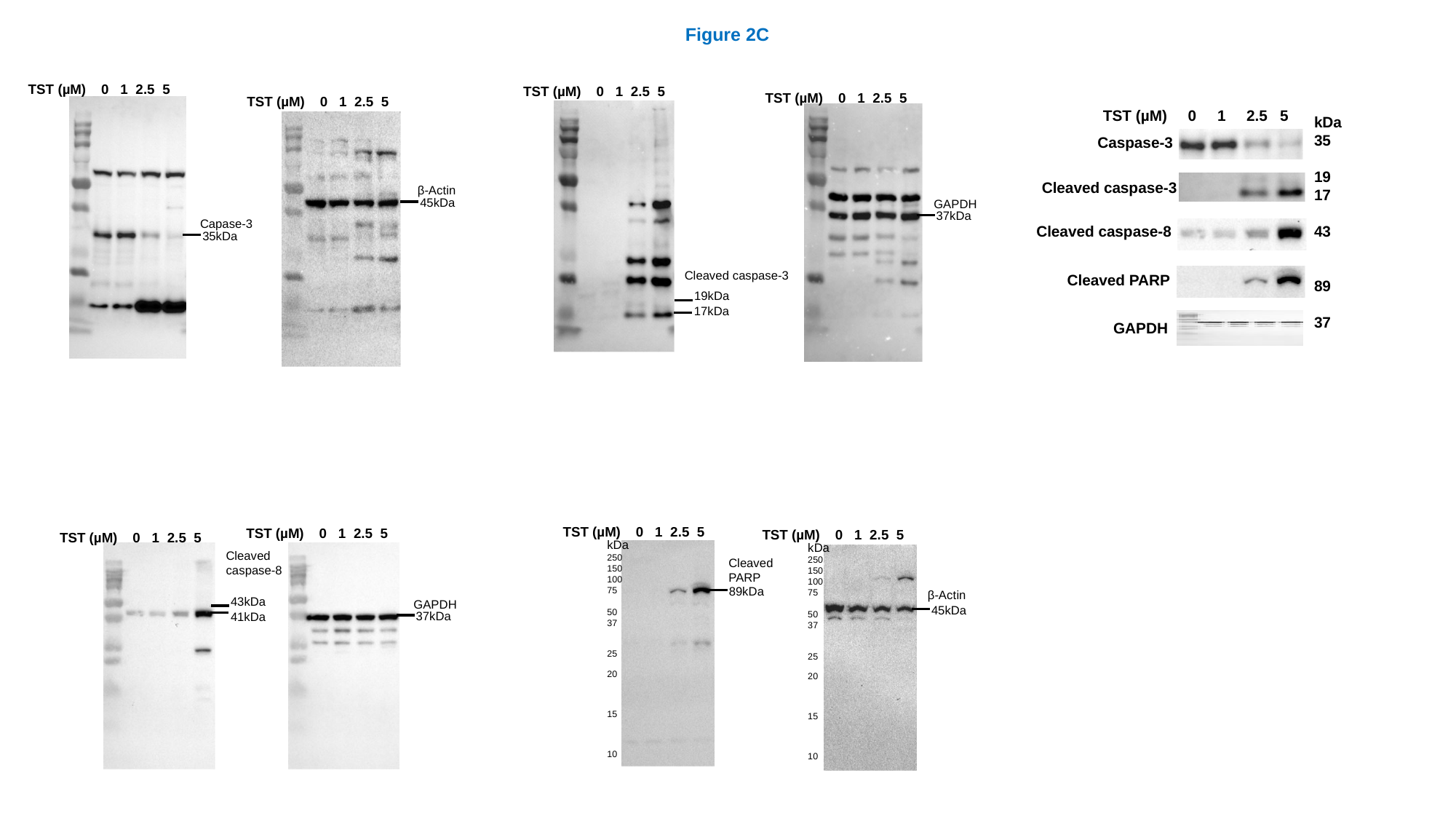

Figure 2C
TST (µM) 0 1 2.5 5
Capase-3
35kDa
TST (µM) 0 1 2.5 5
Cleaved caspase-3
19kDa
17kDa
TST (µM) 0 1 2.5 5
GAPDH
37kDa
TST (µM) 0 1 2.5 5
β-Actin
45kDa
TST (µM) 0 1 2.5 5
kDa
35
19
17
43
89
37
Caspase-3
Cleaved caspase-3
Cleaved caspase-8
Cleaved PARP
GAPDH
TST (µM) 0 1 2.5 5
kDa
250
150
100
75
50
37
25
20
15
10
Cleaved PARP
89kDa
TST (µM) 0 1 2.5 5
kDa
250
150
100
75
50
37
25
20
15
10
β-Actin
45kDa
TST (µM) 0 1 2.5 5
GAPDH
37kDa
TST (µM) 0 1 2.5 5
Cleaved caspase-8
43kDa
41kDa

## Slide 4
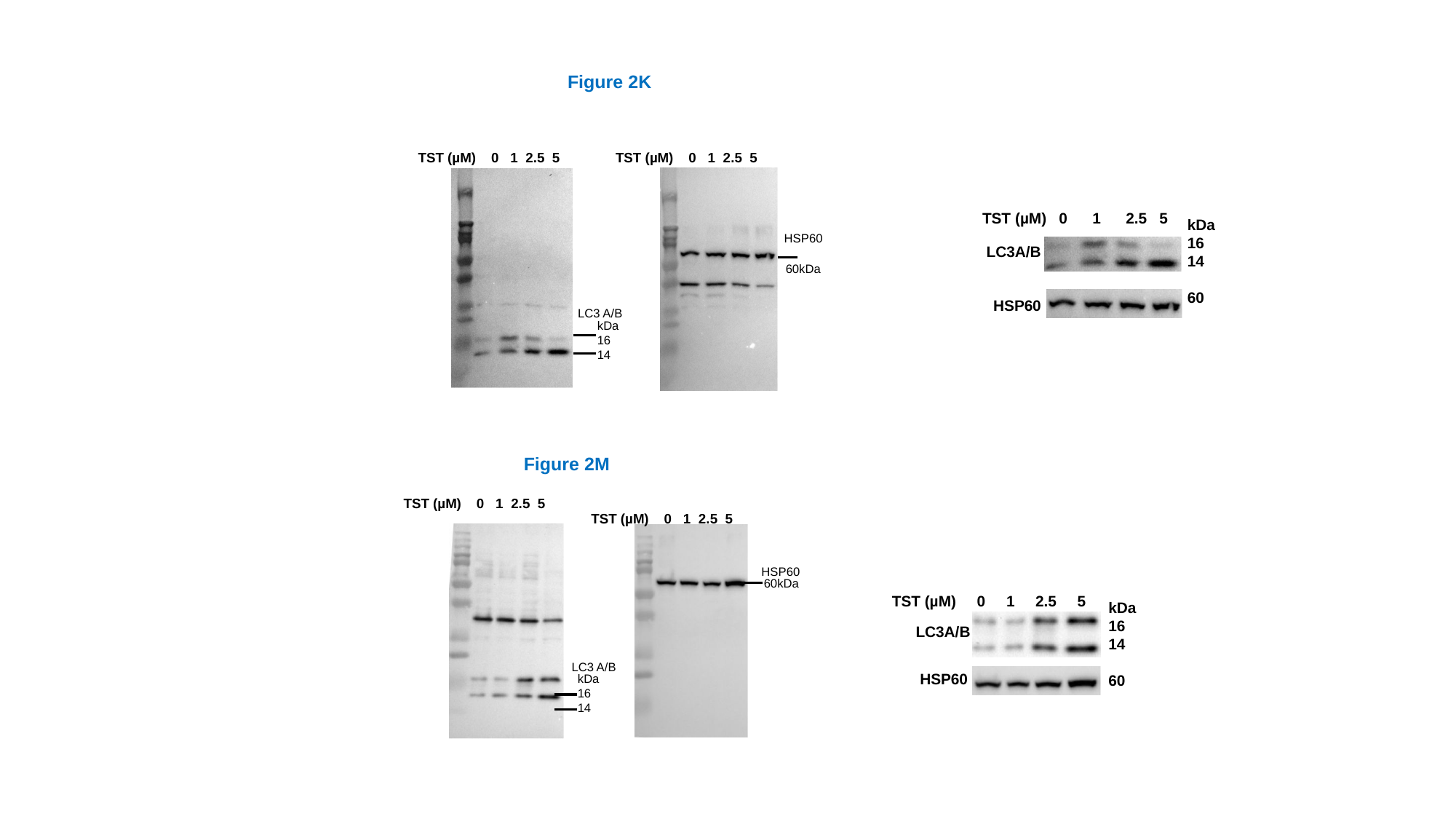

Figure 2K
TST (µM) 0 1 2.5 5
LC3 A/B
kDa
16
14
TST (µM) 0 1 2.5 5
HSP60
60kDa
TST (µM) 0 1 2.5 5
kDa
16
14
60
HSP60
LC3A/B
Figure 2M
TST (µM) 0 1 2.5 5
LC3 A/B
kDa
16
14
TST (µM) 0 1 2.5 5
HSP60
60kDa
TST (µM) 0 1 2.5 5
kDa
16
14
60
LC3A/B
HSP60

## Slide 5
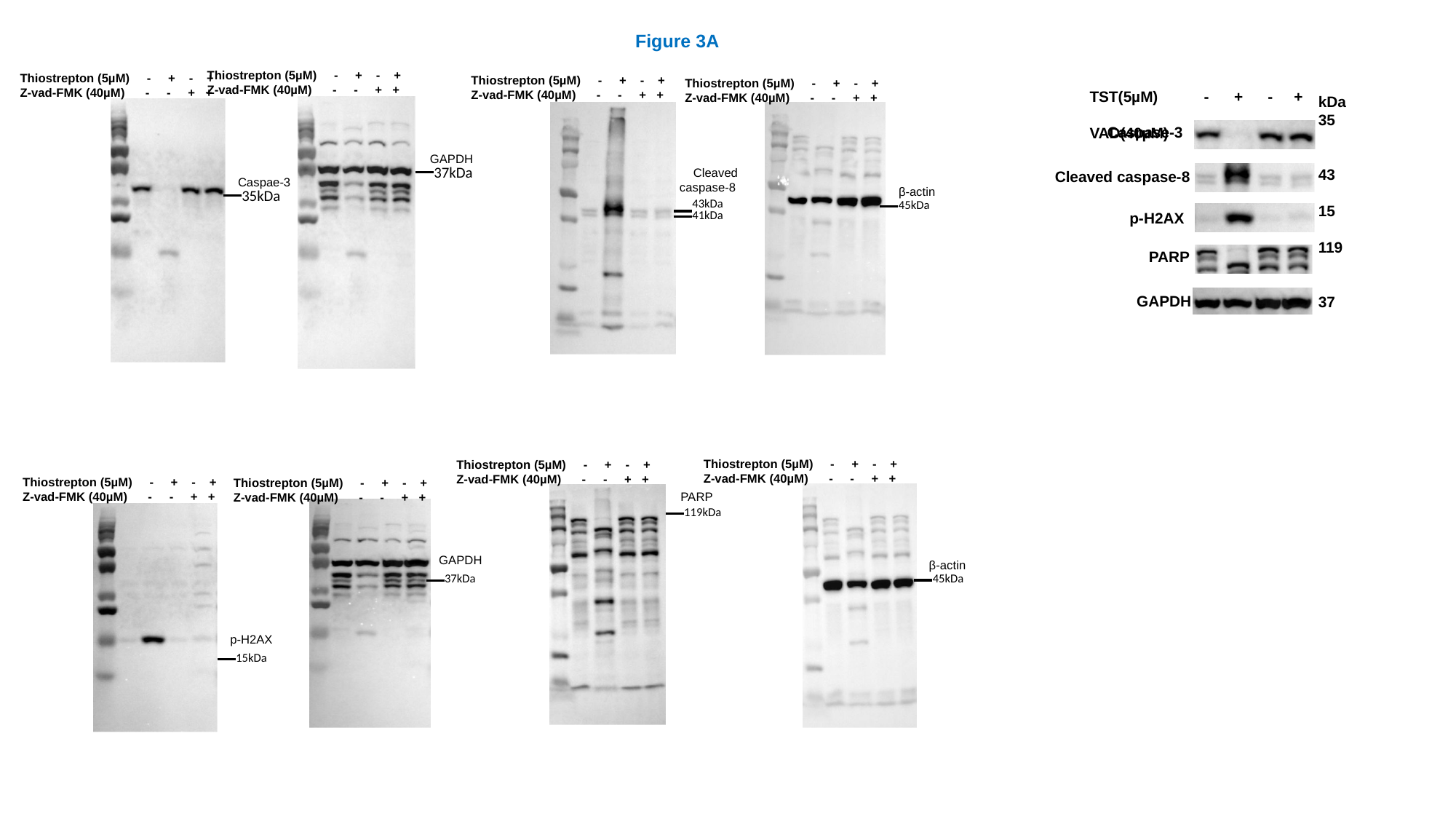

Figure 3A
Thiostrepton (5µM) - + - +
Z-vad-FMK (40µM) - - + +
Thiostrepton (5µM) - + - +
Z-vad-FMK (40µM) - - + +
GAPDH
37kDa
Caspae-3
35kDa
Thiostrepton (5µM) - + - +
Z-vad-FMK (40µM) - - + +
Thiostrepton (5µM) - + - +
Z-vad-FMK (40µM) - - + +
 Cleaved
caspase-8
43kDa
41kDa
β-actin
45kDa
TST(5µM) - + - +
VAD(40µM) - - + +
kDa
35
43
15
119
37
Caspase-3
Cleaved caspase-8
p-H2AX
PARP
GAPDH
Thiostrepton (5µM) - + - +
Z-vad-FMK (40µM) - - + +
β-actin
45kDa
Thiostrepton (5µM) - + - +
Z-vad-FMK (40µM) - - + +
PARP
119kDa
Thiostrepton (5µM) - + - +
Z-vad-FMK (40µM) - - + +
p-H2AX
15kDa
Thiostrepton (5µM) - + - +
Z-vad-FMK (40µM) - - + +
GAPDH
37kDa

## Slide 6
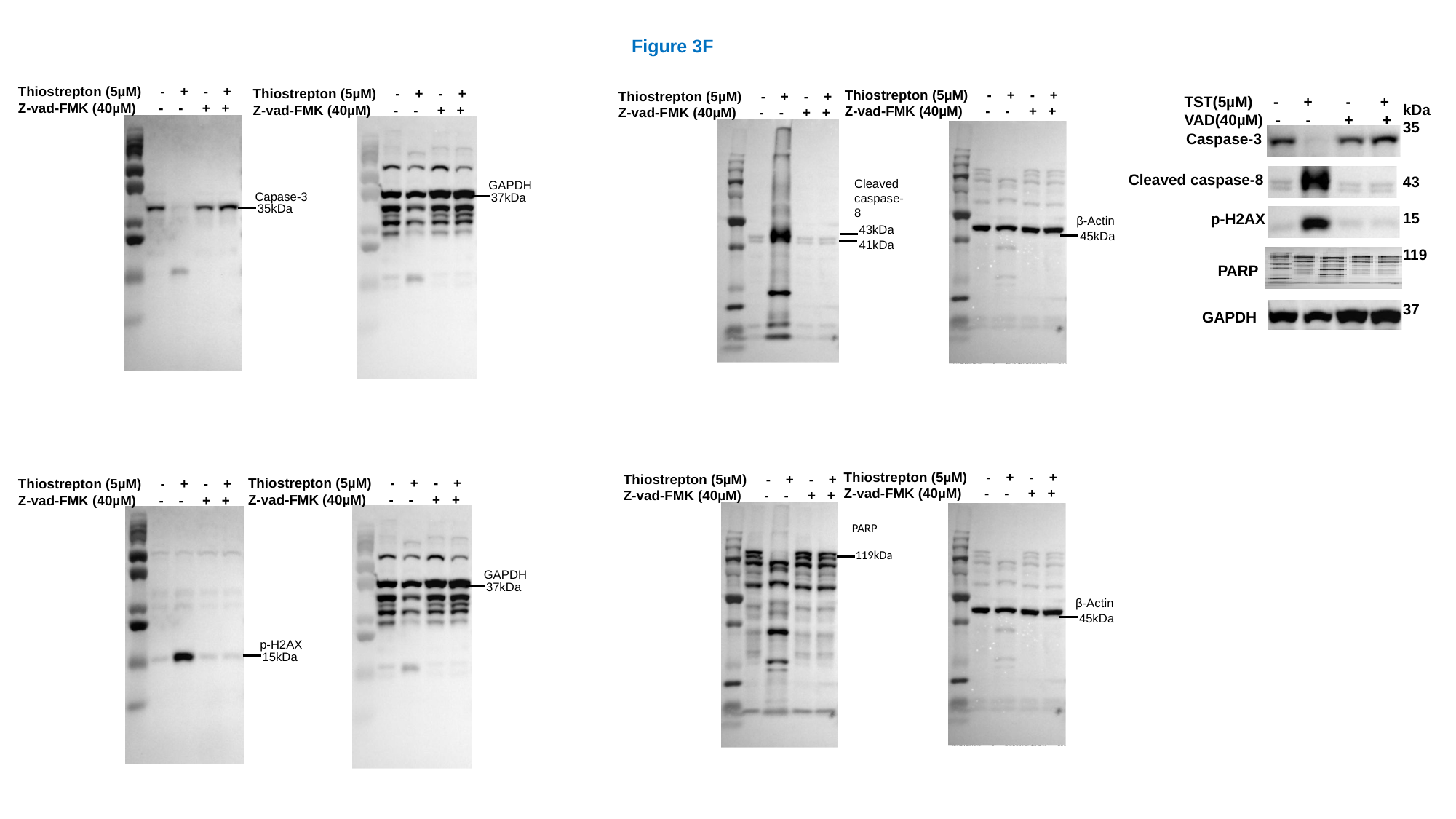

Figure 3F
Thiostrepton (5µM) - + - +
Z-vad-FMK (40µM) - - + +
Capase-3
35kDa
Thiostrepton (5µM) - + - +
Z-vad-FMK (40µM) - - + +
GAPDH
37kDa
Thiostrepton (5µM) - + - +
Z-vad-FMK (40µM) - - + +
β-Actin
45kDa
Thiostrepton (5µM) - + - +
Z-vad-FMK (40µM) - - + +
Cleaved caspase-8
43kDa
41kDa
TST(5µM) - + - +
VAD(40µM) - - + +
kDa
35
43
15
119
37
Caspase-3
Cleaved caspase-8
p-H2AX
PARP
GAPDH
Thiostrepton (5µM) - + - +
Z-vad-FMK (40µM) - - + +
β-Actin
45kDa
Thiostrepton (5µM) - + - +
Z-vad-FMK (40µM) - - + +
Thiostrepton (5µM) - + - +
Z-vad-FMK (40µM) - - + +
GAPDH
37kDa
Thiostrepton (5µM) - + - +
Z-vad-FMK (40µM) - - + +
p-H2AX
15kDa
PARP
119kDa

## Slide 7
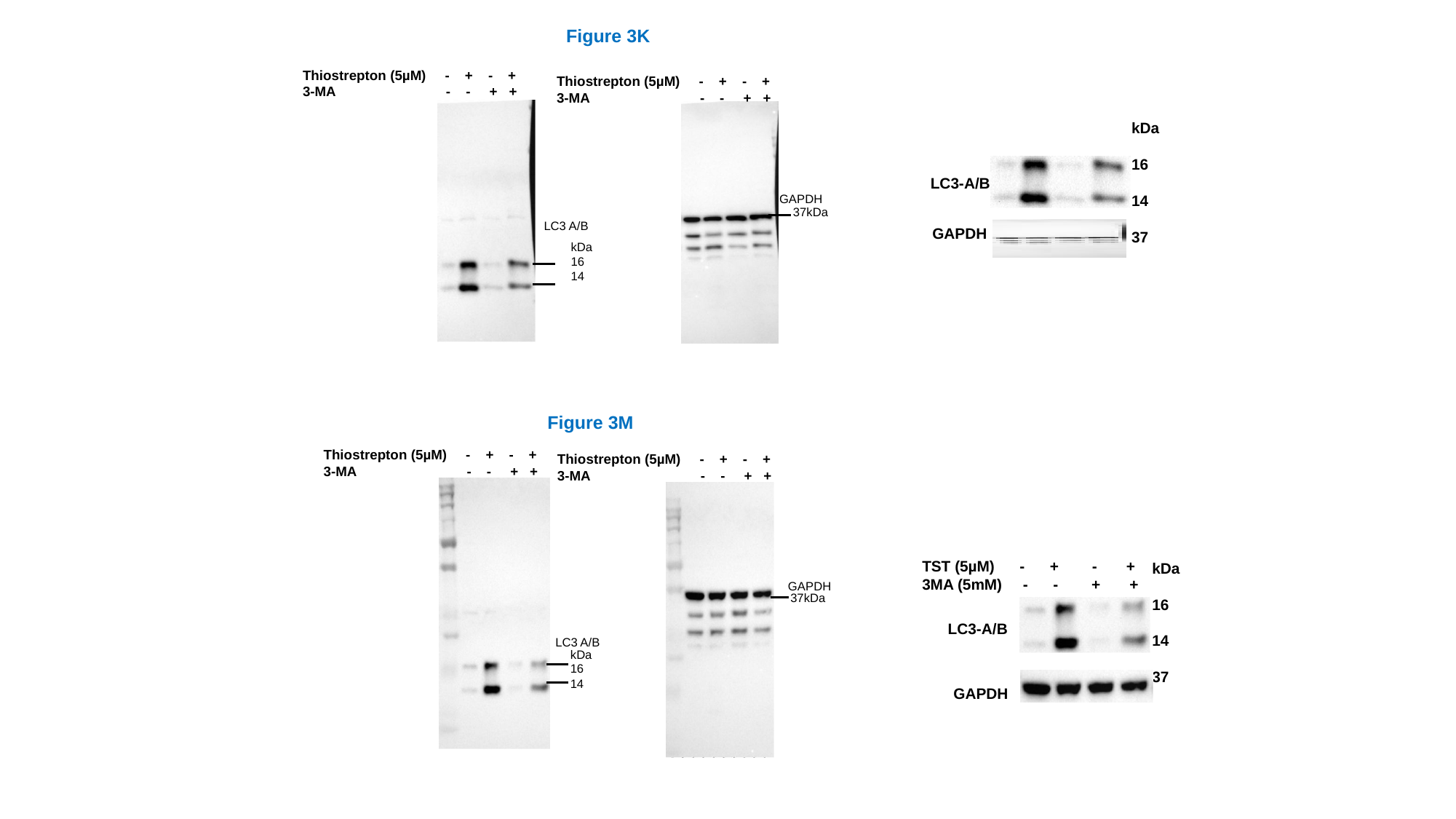

Figure 3K
Thiostrepton (5µM) - + - +
3-MA - - + +
Thiostrepton (5µM) - + - +
3-MA - - + +
LC3 A/B
kDa
16
14
GAPDH
37kDa
kDa
16
14
37
LC3-A/B
GAPDH
Figure 3M
Thiostrepton (5µM) - + - +
3-MA - - + +
LC3 A/B
kDa
16
14
Thiostrepton (5µM) - + - +
3-MA - - + +
GAPDH
37kDa
TST (5µM) - + - +
3MA (5mM) - - + +
kDa
16
14
37
LC3-A/B
GAPDH

## Slide 8
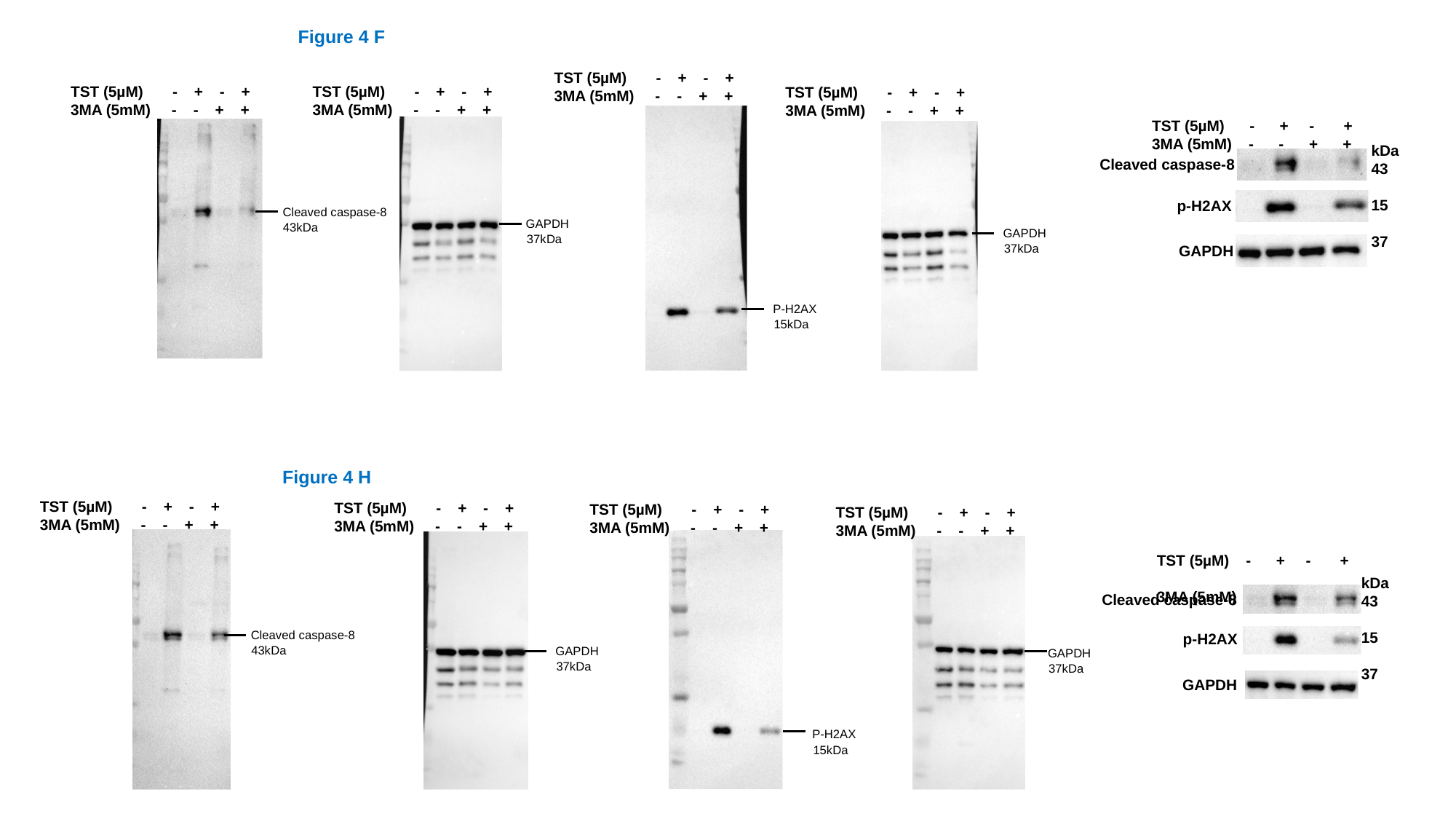

Figure 4 F
TST (5µM) - + - +
3MA (5mM) - - + +
P-H2AX
15kDa
TST (5µM) - + - +
3MA (5mM) - - + +
GAPDH
37kDa
TST (5µM) - + - +
3MA (5mM) - - + +
Cleaved caspase-8
43kDa
TST (5µM) - + - +
3MA (5mM) - - + +
GAPDH
37kDa
TST (5µM) - + - +
3MA (5mM) - - + +
kDa
43
15
37
Cleaved caspase-8
p-H2AX
GAPDH
Figure 4 H
TST (5µM) - + - +
3MA (5mM) - - + +
Cleaved caspase-8
43kDa
TST (5µM) - + - +
3MA (5mM) - - + +
GAPDH
37kDa
TST (5µM) - + - +
3MA (5mM) - - + +
P-H2AX
15kDa
TST (5µM) - + - +
3MA (5mM) - - + +
GAPDH
37kDa
TST (5µM) - + - +
3MA (5mM) - - + +
kDa
43
15
37
Cleaved caspase-8
p-H2AX
GAPDH

## Slide 9
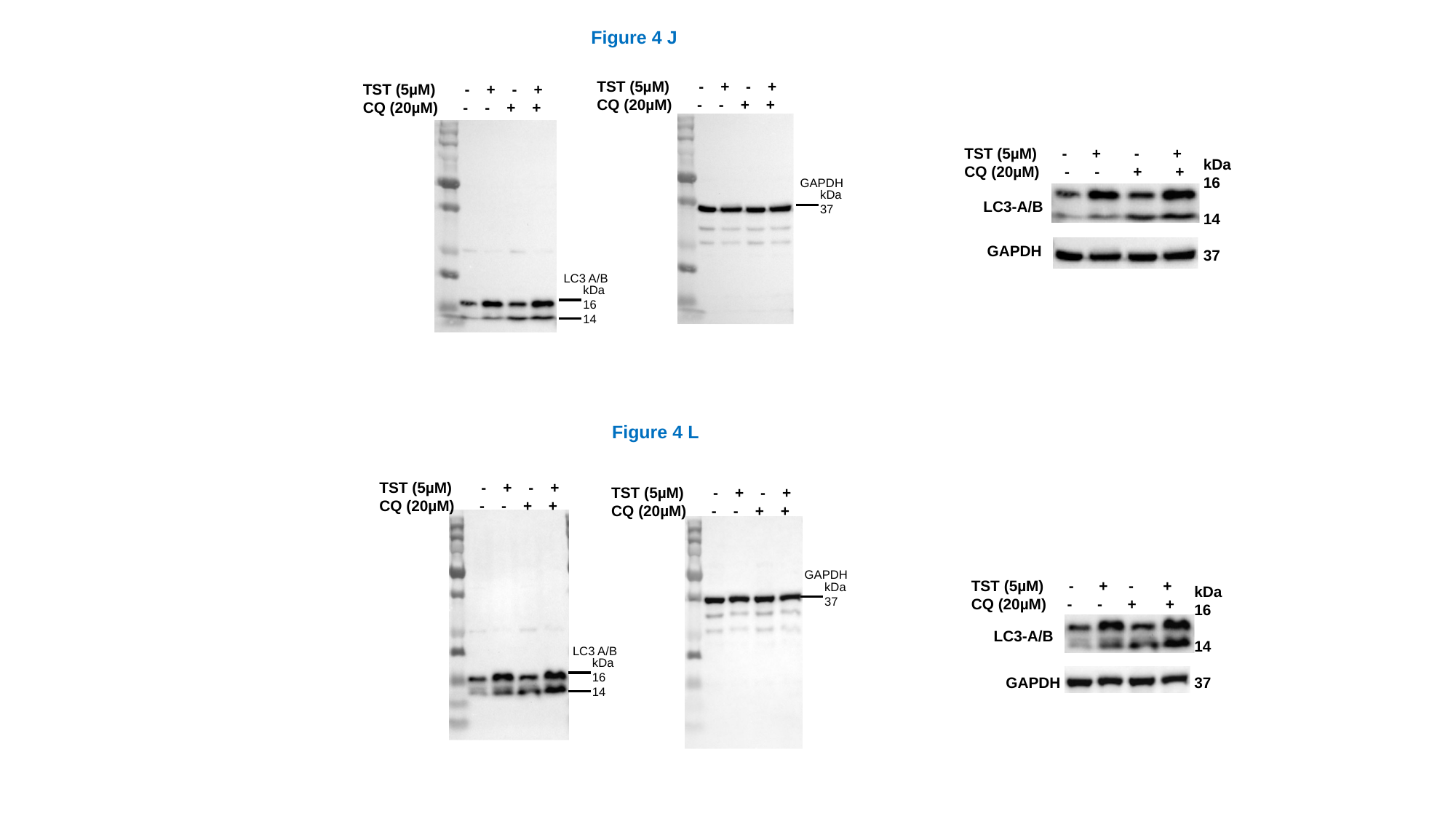

Figure 4 J
TST (5µM) - + - +
CQ (20µM) - - + +
GAPDH
kDa
37
TST (5µM) - + - +
CQ (20µM) - - + +
LC3 A/B
kDa
16
14
TST (5µM) - + - +
CQ (20µM) - - + +
kDa
16
14
37
LC3-A/B
GAPDH
Figure 4 L
TST (5µM) - + - +
CQ (20µM) - - + +
LC3 A/B
kDa
16
14
TST (5µM) - + - +
CQ (20µM) - - + +
GAPDH
kDa
37
TST (5µM) - + - +
CQ (20µM) - - + +
kDa
16
14
37
LC3-A/B
GAPDH

## Slide 10
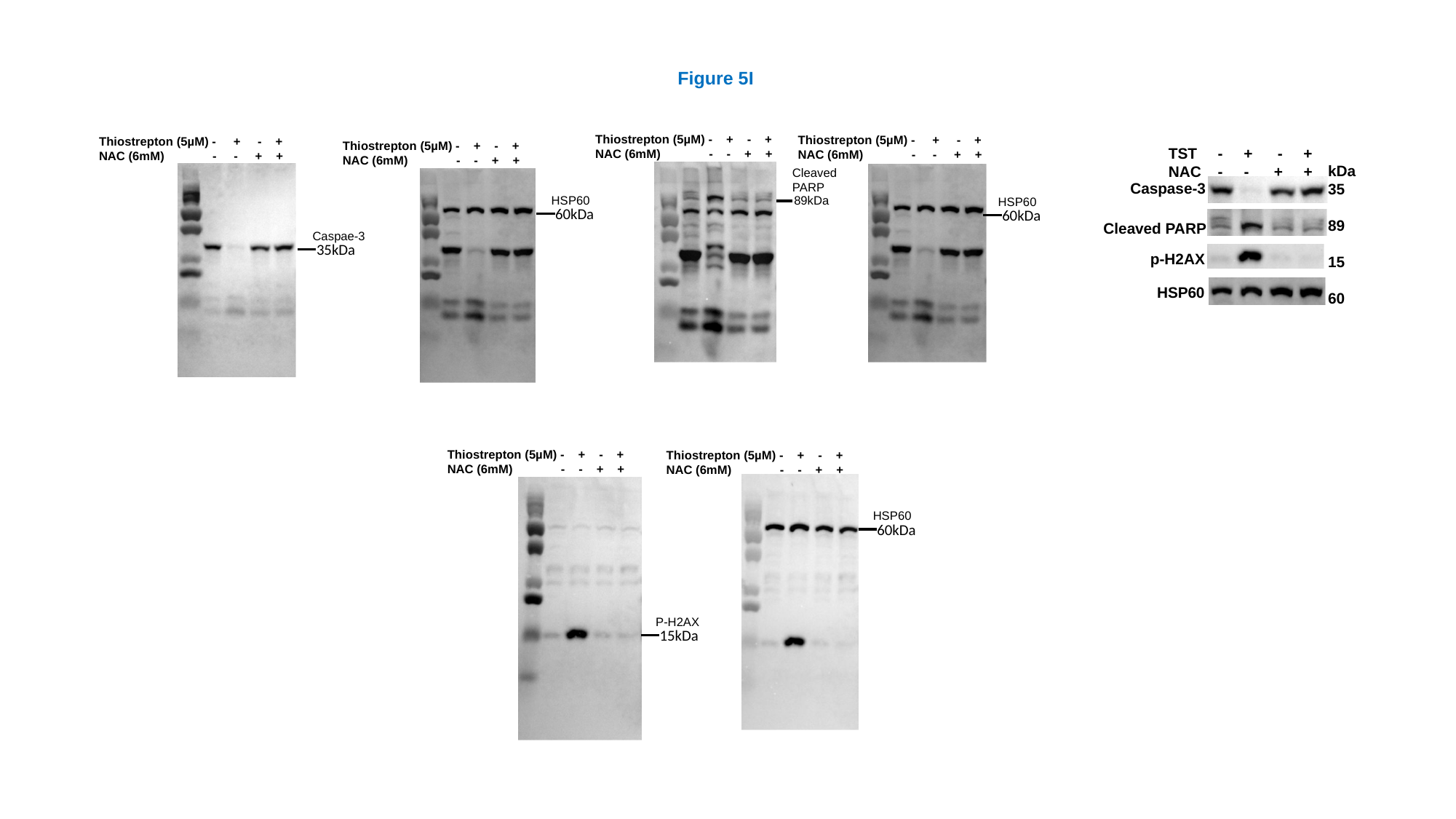

Figure 5I
Thiostrepton (5µM) - + - +
NAC (6mM) - - + +
Cleaved
PARP
89kDa
Thiostrepton (5µM) - + - +
NAC (6mM) - - + +
HSP60
60kDa
Thiostrepton (5µM) - + - +
NAC (6mM) - - + +
Caspae-3
35kDa
Thiostrepton (5µM) - + - +
NAC (6mM) - - + +
HSP60
60kDa
TST - + - +
NAC - - + +
kDa
35
89
15
60
Caspase-3
Cleaved PARP
p-H2AX
HSP60
Thiostrepton (5µM) - + - +
NAC (6mM) - - + +
P-H2AX
15kDa
Thiostrepton (5µM) - + - +
NAC (6mM) - - + +
HSP60
60kDa

## Slide 11
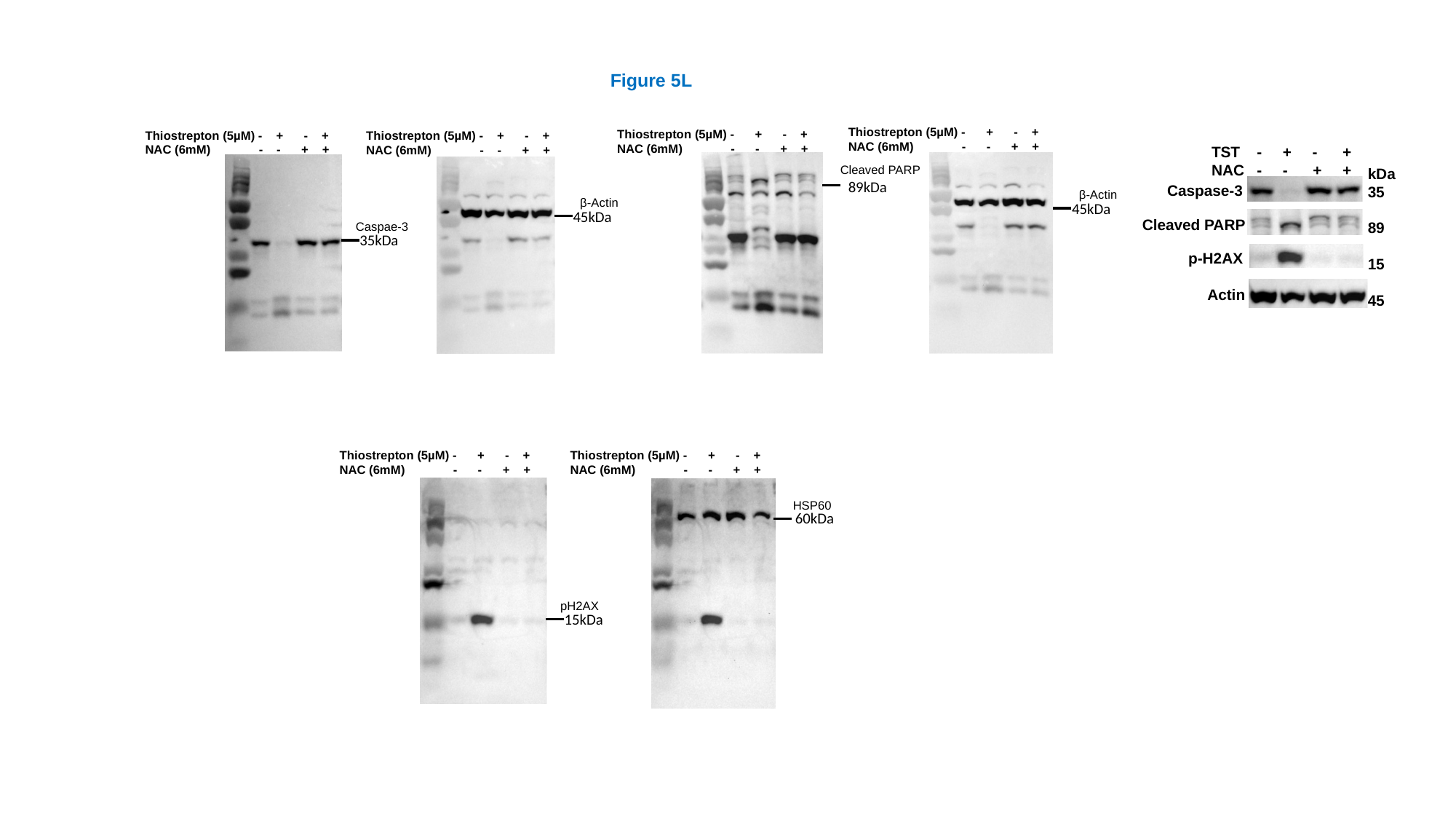

Figure 5L
Thiostrepton (5µM) - + - +
NAC (6mM) - - + +
β-Actin
45kDa
Thiostrepton (5µM) - + - +
NAC (6mM) - - + +
Cleaved PARP
89kDa
Thiostrepton (5µM) - + - +
NAC (6mM) - - + +
Caspae-3
35kDa
Thiostrepton (5µM) - + - +
NAC (6mM) - - + +
β-Actin
45kDa
TST - + - +
NAC - - + +
kDa
35
89
15
45
Caspase-3
Cleaved PARP
p-H2AX
Actin
Thiostrepton (5µM) - + - +
NAC (6mM) - - + +
pH2AX
15kDa
Thiostrepton (5µM) - + - +
NAC (6mM) - - + +
HSP60
60kDa

## Slide 12
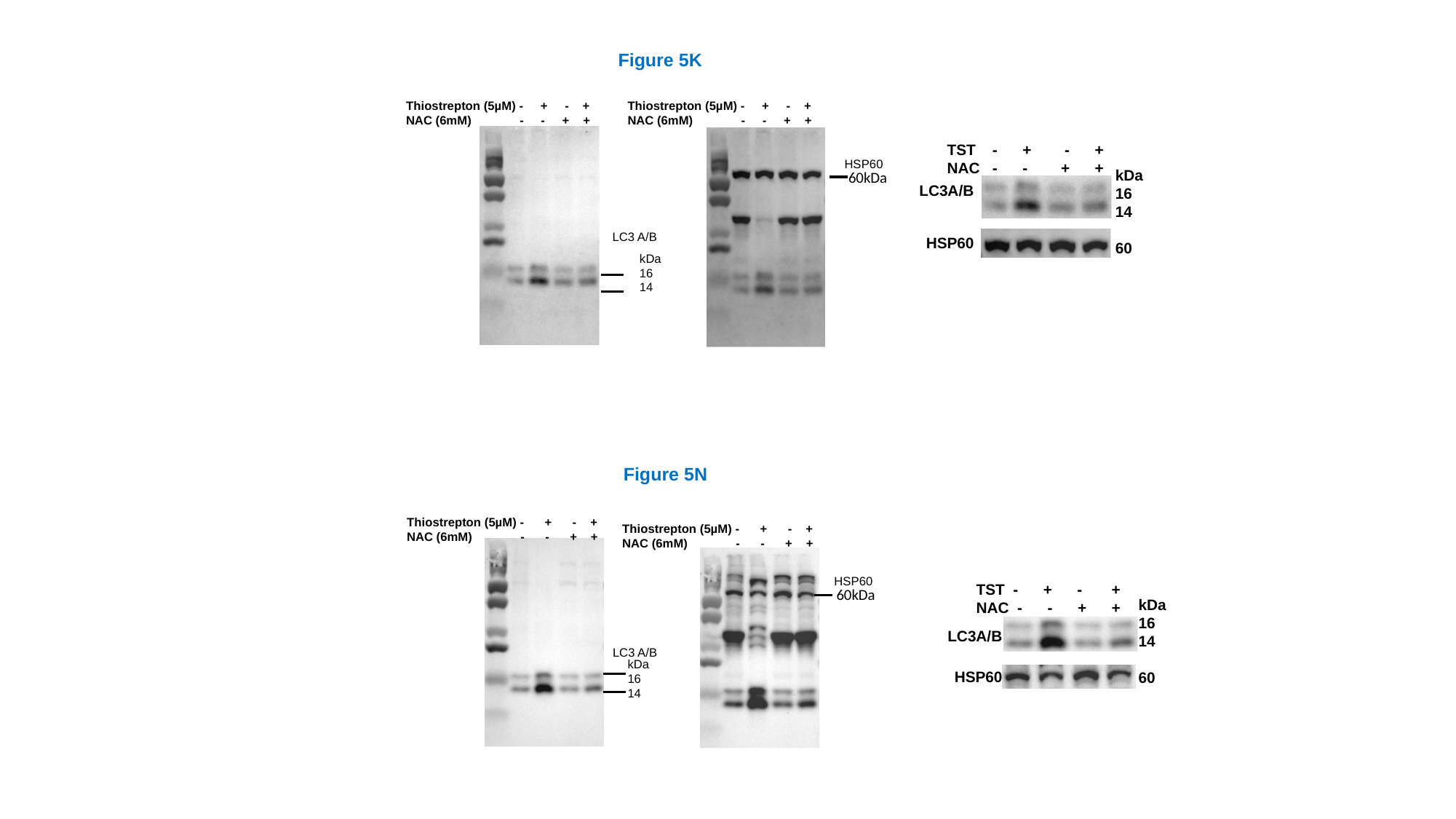

Figure 5K
Thiostrepton (5µM) - + - +
NAC (6mM) - - + +
LC3 A/B
kDa
16
14
Thiostrepton (5µM) - + - +
NAC (6mM) - - + +
HSP60
60kDa
TST - + - +
NAC - - + +
kDa
16
14
60
LC3A/B
HSP60
Figure 5N
Thiostrepton (5µM) - + - +
NAC (6mM) - - + +
LC3 A/B
kDa
16
14
Thiostrepton (5µM) - + - +
NAC (6mM) - - + +
HSP60
60kDa
TST - + - +
NAC - - + +
kDa
16
14
60
LC3A/B
HSP60

## Slide 13
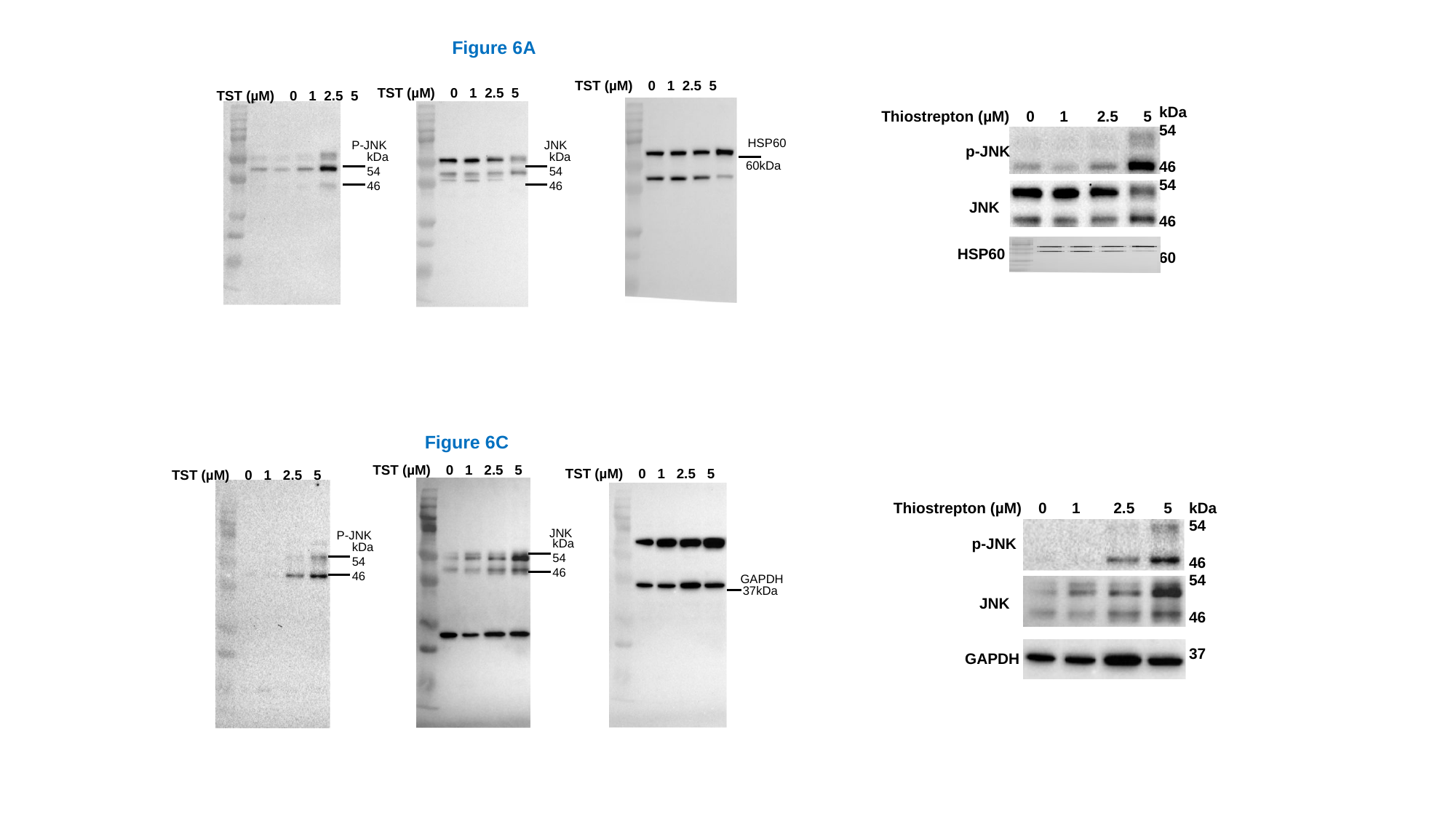

Figure 6A
TST (µM) 0 1 2.5 5
 JNK
kDa
54
46
TST (µM) 0 1 2.5 5
P-JNK
kDa
54
46
TST (µM) 0 1 2.5 5
HSP60
60kDa
kDa
54
46
54
46
60
Thiostrepton (µM) 0 1 2.5 5
p-JNK
JNK
HSP60
Figure 6C
TST (µM) 0 1 2.5 5
JNK
kDa
54
46
TST (µM) 0 1 2.5 5
GAPDH
37kDa
TST (µM) 0 1 2.5 5
P-JNK
kDa
54
46
Thiostrepton (µM) 0 1 2.5 5
kDa
54
46
54
46
37
p-JNK
JNK
GAPDH

## Slide 14
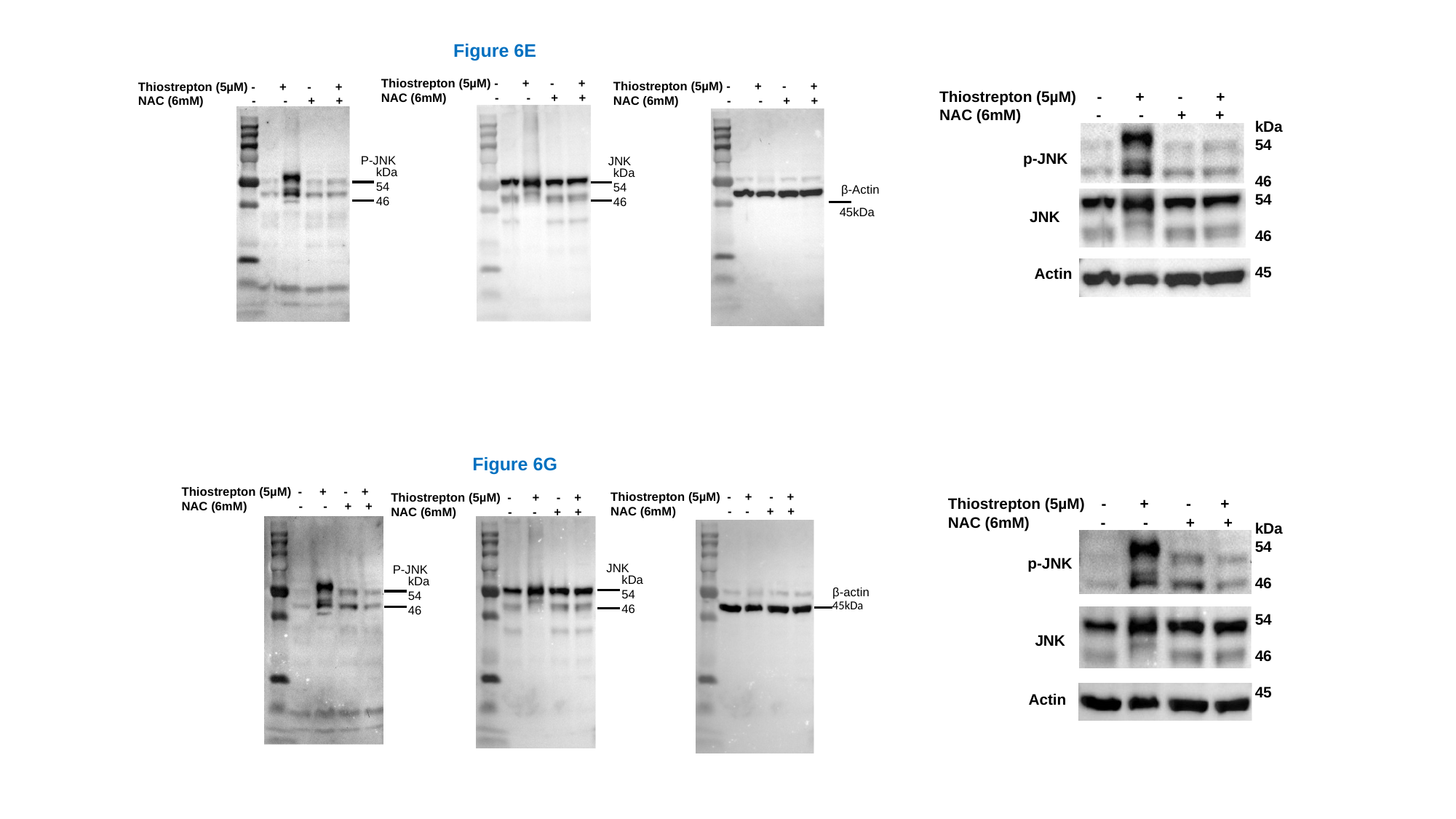

Figure 6E
Thiostrepton (5µM) - + - +
NAC (6mM) - - + +
 JNK
kDa
54
46
Thiostrepton (5µM) - + - +
NAC (6mM) - - + +
β-Actin
45kDa
Thiostrepton (5µM) - + - +
NAC (6mM) - - + +
P-JNK
kDa
54
46
Thiostrepton (5µM) - + - +
NAC (6mM) - - + +
kDa
54
46
54
46
45
p-JNK
JNK
Actin
Figure 6G
Thiostrepton (5µM) - + - +
NAC (6mM) - - + +
P-JNK
kDa
54
46
Thiostrepton (5µM) - + - +
NAC (6mM) - - + +
β-actin
45kDa
Thiostrepton (5µM) - + - +
NAC (6mM) - - + +
JNK
kDa
54
46
Thiostrepton (5µM) - + - +
NAC (6mM) - - + +
kDa
54
46
54
46
45
p-JNK
JNK
Actin

## Slide 15
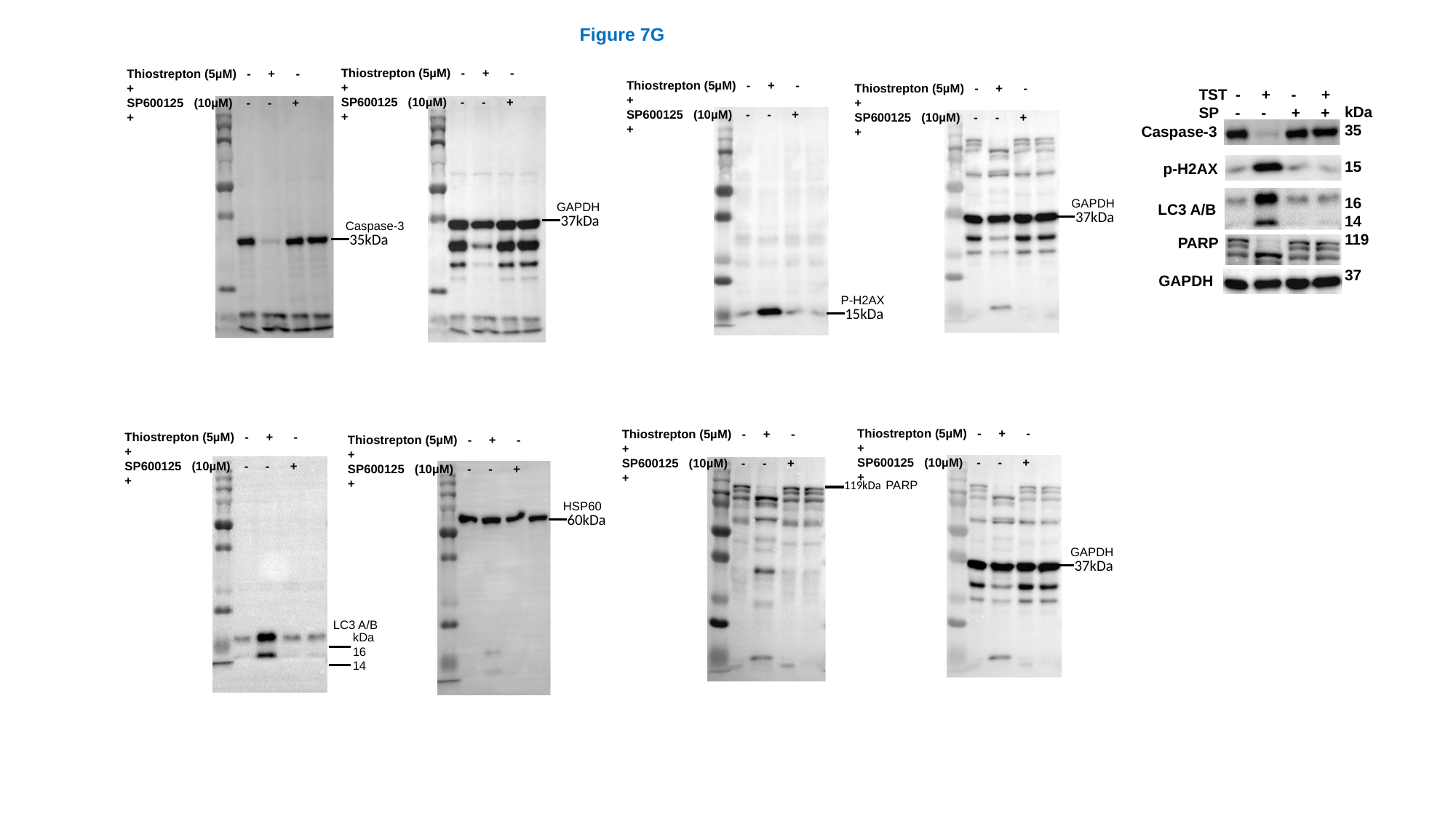

Figure 7G
Thiostrepton (5µM) - + - +
SP600125 (10µM) - - + +
GAPDH
37kDa
Thiostrepton (5µM) - + - +
SP600125 (10µM) - - + +
Caspase-3
35kDa
Thiostrepton (5µM) - + - +
SP600125 (10µM) - - + +
P-H2AX
15kDa
Thiostrepton (5µM) - + - +
SP600125 (10µM) - - + +
GAPDH
37kDa
Thiostrepton (5µM) - + - +
SP600125 (10µM) - - + +
GAPDH
37kDa
Thiostrepton (5µM) - + - +
SP600125 (10µM) - - + +
119kDa
PARP
Thiostrepton (5µM) - + - +
SP600125 (10µM) - - + +
LC3 A/B
kDa
16
14
Thiostrepton (5µM) - + - +
SP600125 (10µM) - - + +
HSP60
60kDa
kDa
35
15
16
14
119
37
TST - + - +
SP - - + +
Caspase-3
p-H2AX
LC3 A/B
PARP
GAPDH

## Slide 16
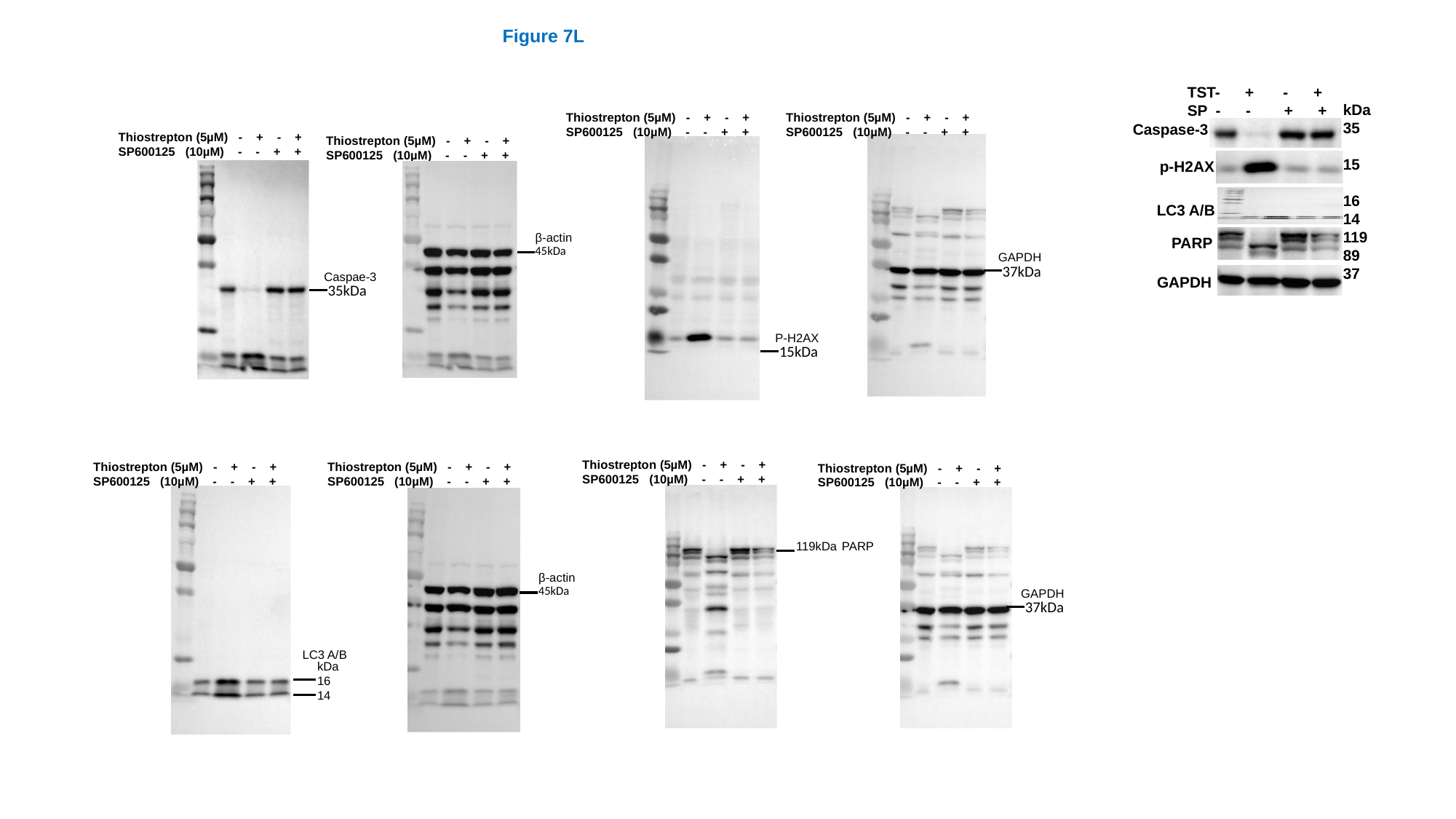

Figure 7L
kDa
35
15
16
14
119
89
37
TST- + - +
SP - - + +
Caspase-3
p-H2AX
PARP
GAPDH
LC3 A/B
Thiostrepton (5µM) - + - +
SP600125 (10µM) - - + +
P-H2AX
15kDa
Thiostrepton (5µM) - + - +
SP600125 (10µM) - - + +
GAPDH
37kDa
Thiostrepton (5µM) - + - +
SP600125 (10µM) - - + +
Caspae-3
35kDa
Thiostrepton (5µM) - + - +
SP600125 (10µM) - - + +
β-actin
45kDa
Thiostrepton (5µM) - + - +
SP600125 (10µM) - - + +
119kDa
PARP
Thiostrepton (5µM) - + - +
SP600125 (10µM) - - + +
GAPDH
37kDa
Thiostrepton (5µM) - + - +
SP600125 (10µM) - - + +
β-actin
45kDa
Thiostrepton (5µM) - + - +
SP600125 (10µM) - - + +
LC3 A/B
kDa
16
14

## Slide 17
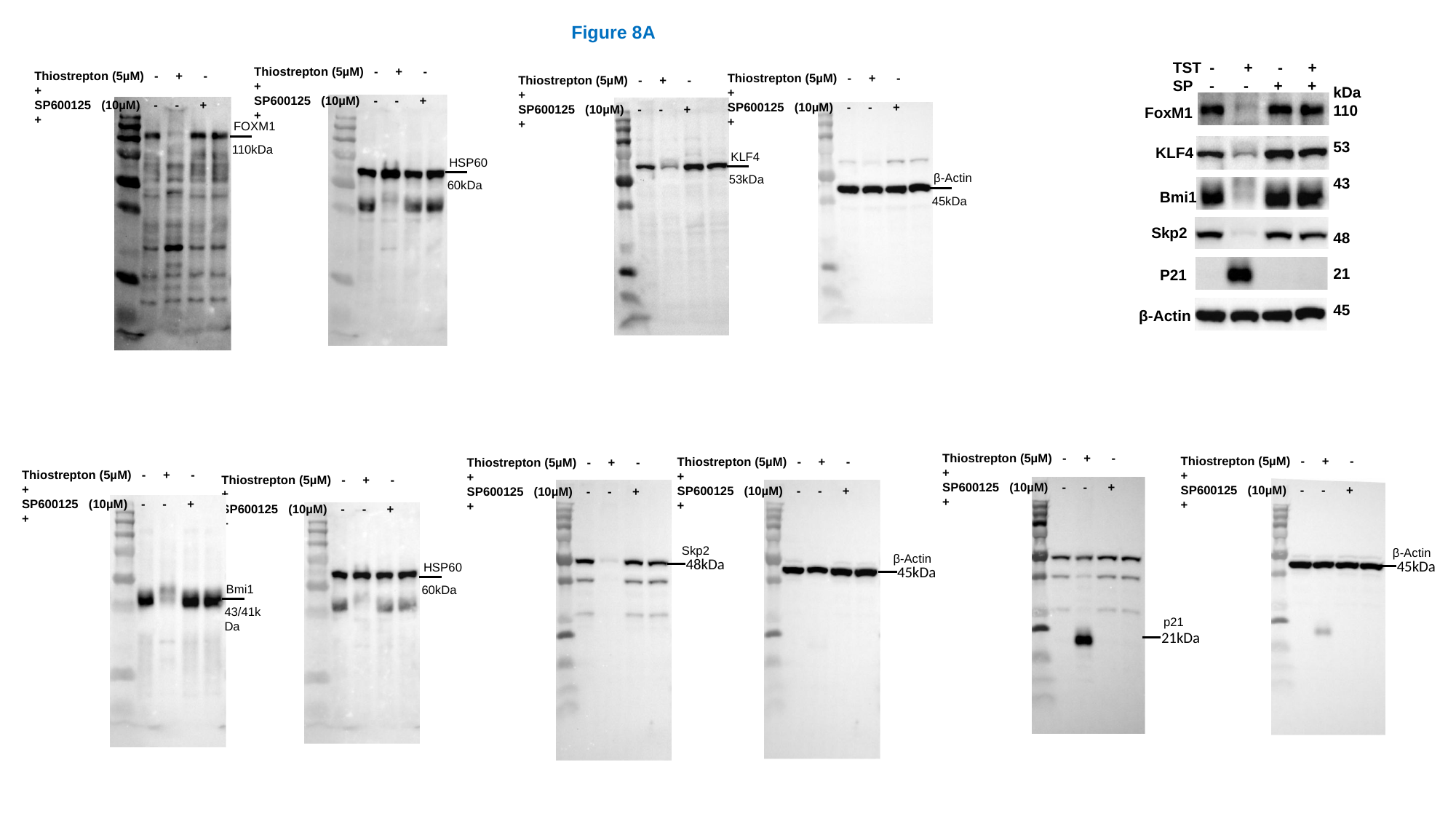

Figure 8A
TST - + - +
SP - - + +
kDa
110
53
43
48
21
45
FoxM1
KLF4
Bmi1
Skp2
P21
β-Actin
Thiostrepton (5µM) - + - +
SP600125 (10µM) - - + +
HSP60
60kDa
Thiostrepton (5µM) - + - +
SP600125 (10µM) - - + +
FOXM1
110kDa
Thiostrepton (5µM) - + - +
SP600125 (10µM) - - + +
β-Actin
45kDa
Thiostrepton (5µM) - + - +
SP600125 (10µM) - - + +
KLF4
53kDa
Thiostrepton (5µM) - + - +
SP600125 (10µM) - - + +
p21
21kDa
β-Actin
45kDa
Thiostrepton (5µM) - + - +
SP600125 (10µM) - - + +
Thiostrepton (5µM) - + - +
SP600125 (10µM) - - + +
Thiostrepton (5µM) - + - +
SP600125 (10µM) - - + +
Skp2
48kDa
β-Actin
45kDa
Thiostrepton (5µM) - + - +
SP600125 (10µM) - - + +
Bmi1
43/41kDa
Thiostrepton (5µM) - + - +
SP600125 (10µM) - - + +
HSP60
60kDa

## Slide 18
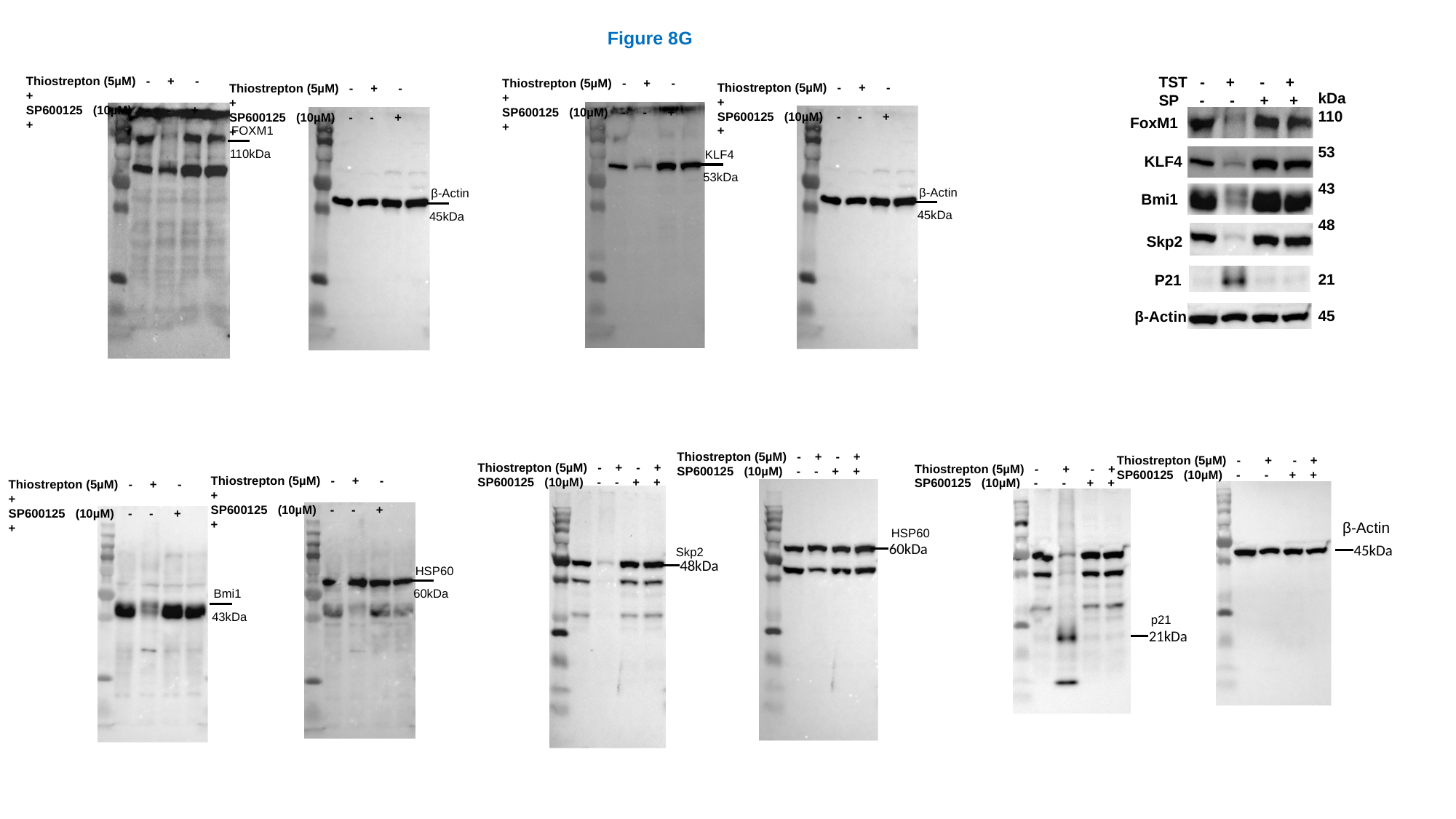

Figure 8G
TST - + - +
SP - - + +
kDa
110
53
43
48
21
45
FoxM1
KLF4
Bmi1
Skp2
P21
β-Actin
Thiostrepton (5µM) - + - +
SP600125 (10µM) - - + +
FOXM1
110kDa
Thiostrepton (5µM) - + - +
SP600125 (10µM) - - + +
β-Actin
45kDa
Thiostrepton (5µM) - + - +
SP600125 (10µM) - - + +
KLF4
53kDa
Thiostrepton (5µM) - + - +
SP600125 (10µM) - - + +
β-Actin
45kDa
Thiostrepton (5µM) - + - +
SP600125 (10µM) - - + +
HSP60
60kDa
Thiostrepton (5µM) - + - +
SP600125 (10µM) - - + +
Skp2
48kDa
Thiostrepton (5µM) - + - +
SP600125 (10µM) - - + +
β-Actin
45kDa
Thiostrepton (5µM) - + - +
SP600125 (10µM) - - + +
p21
21kDa
Thiostrepton (5µM) - + - +
SP600125 (10µM) - - + +
HSP60
60kDa
Thiostrepton (5µM) - + - +
SP600125 (10µM) - - + +
Bmi1
43kDa

## Slide 19
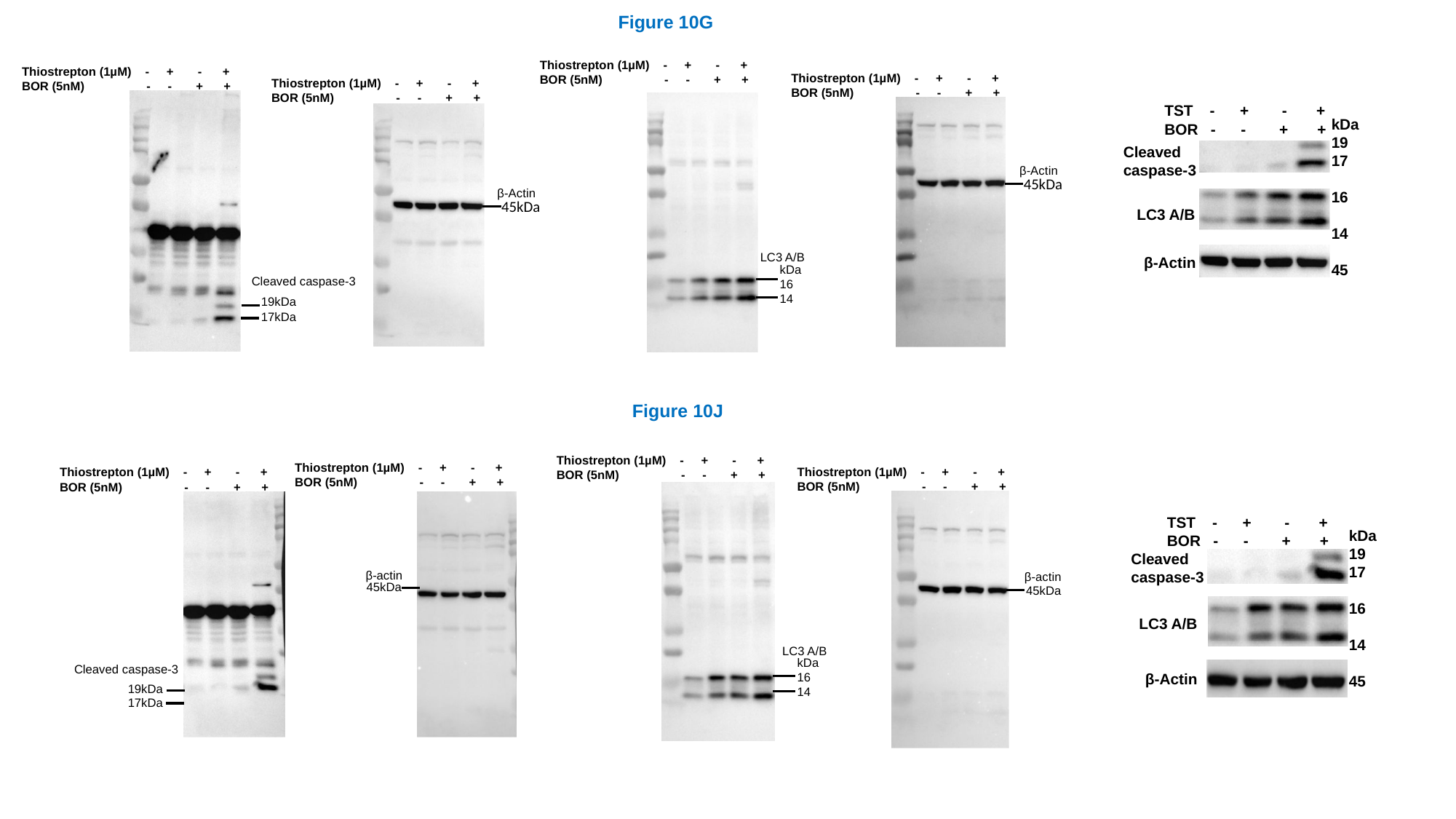

Figure 10G
Thiostrepton (1µM) - + - +
BOR (5nM) - - + +
LC3 A/B
kDa
16
14
Thiostrepton (1µM) - + - +
BOR (5nM) - - + +
β-Actin
45kDa
Thiostrepton (1µM) - + - +
BOR (5nM) - - + +
Cleaved caspase-3
19kDa
17kDa
Thiostrepton (1µM) - + - +
BOR (5nM) - - + +
β-Actin
45kDa
TST - + - +
BOR - - + +
kDa
19
17
16
14
45
Cleaved caspase-3
LC3 A/B
β-Actin
Figure 10J
Thiostrepton (1µM) - + - +
BOR (5nM) - - + +
LC3 A/B
kDa
16
14
Thiostrepton (1µM) - + - +
BOR (5nM) - - + +
β-actin
45kDa
Thiostrepton (1µM) - + - +
BOR (5nM) - - + +
β-actin
45kDa
Thiostrepton (1µM) - + - +
BOR (5nM) - - + +
Cleaved caspase-3
19kDa
17kDa
TST - + - +
BOR - - + +
kDa
19
17
16
14
45
LC3 A/B
β-Actin
Cleaved caspase-3

## Slide 20
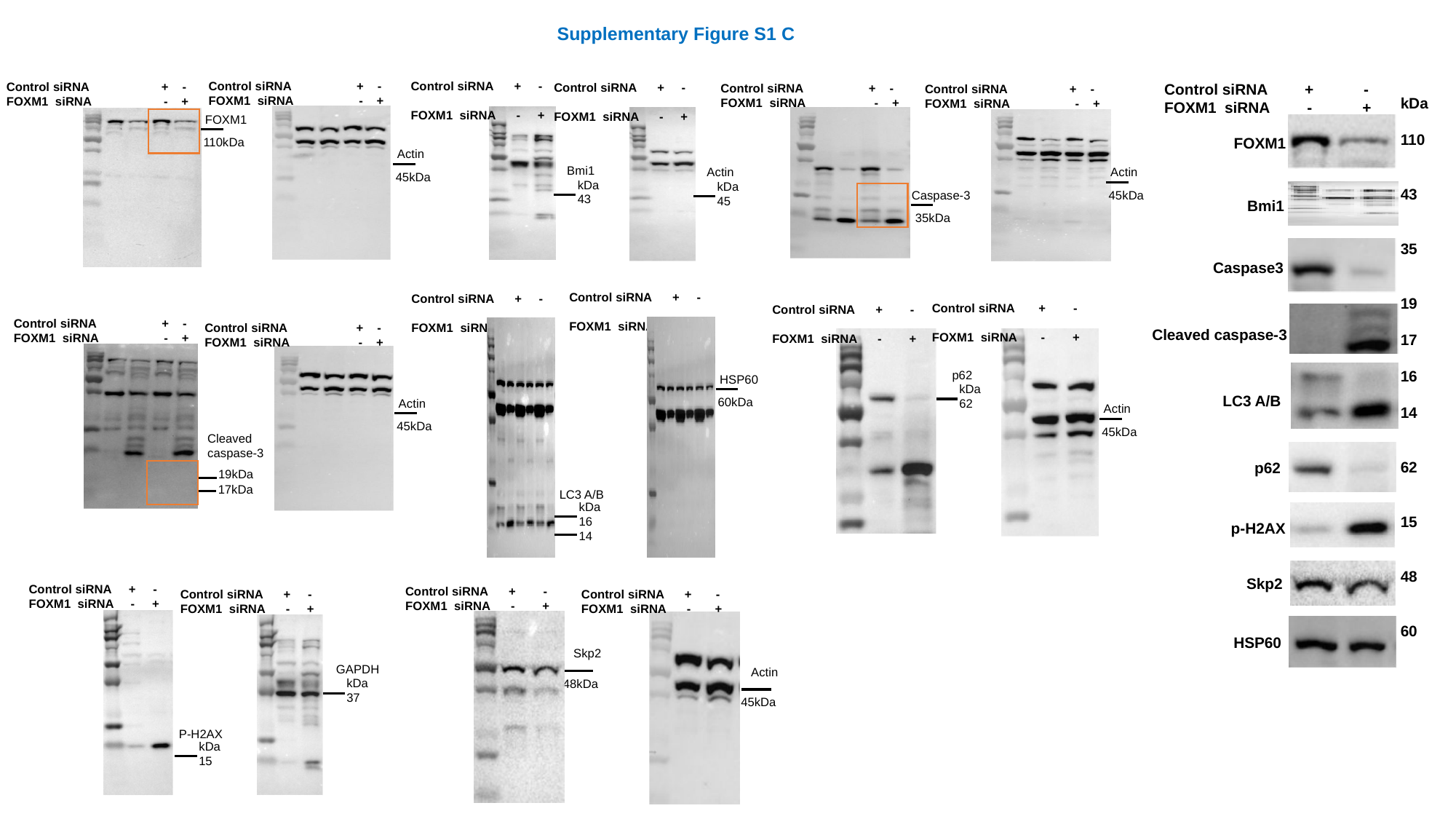

Supplementary Figure S1 C
kDa
110
43
35
19
17
16
14
62
15
48
60
Control siRNA + -
FOXM1 siRNA - +
FOXM1
Bmi1
Caspase3
Cleaved caspase-3
LC3 A/B
p62
p-H2AX
Skp2
HSP60
Control siRNA + -
FOXM1 siRNA - +
Actin
45kDa
Control siRNA + -
FOXM1 siRNA - +
FOXM1
110kDa
Control siRNA + -
FOXM1 siRNA - +
Bmi1
kDa
43
Control siRNA + -
FOXM1 siRNA - +
Actin
kDa
45
Control siRNA + -
FOXM1 siRNA - +
Control siRNA + -
FOXM1 siRNA - +
Caspase-3
35kDa
Actin
45kDa
Control siRNA + -
FOXM1 siRNA - +
HSP60
60kDa
Control siRNA + -
FOXM1 siRNA - +
LC3 A/B
kDa
16
14
Control siRNA + -
FOXM1 siRNA - +
Actin
45kDa
Control siRNA + -
FOXM1 siRNA - +
p62
kDa
62
Control siRNA + -
FOXM1 siRNA - +
Cleaved
caspase-3
19kDa
17kDa
Control siRNA + -
FOXM1 siRNA - +
Actin
45kDa
Control siRNA + -
FOXM1 siRNA - +
P-H2AX
kDa
15
Control siRNA + -
FOXM1 siRNA - +
GAPDH
kDa
37
Control siRNA + -
FOXM1 siRNA - +
Skp2
48kDa
Control siRNA + -
FOXM1 siRNA - +
Actin
45kDa
