# Supplementary material for: Targeting FOXM1 regulates metabolic signatures through ROS-dependent JNK/Bmi1/Skp2 axis in human cutaneous T-cell lymphoma
Source: Cell Death Dis. 2026 Jan 7;17(1):170. doi: 10.1038/s41419-025-08389-z (PMC12876963; doi:10.1038/s41419-025-08389-z)
Supplement: Supplementary file 2 — Supplementary Figures [file 41419_2025_8389_MOESM2_ESM.docx]

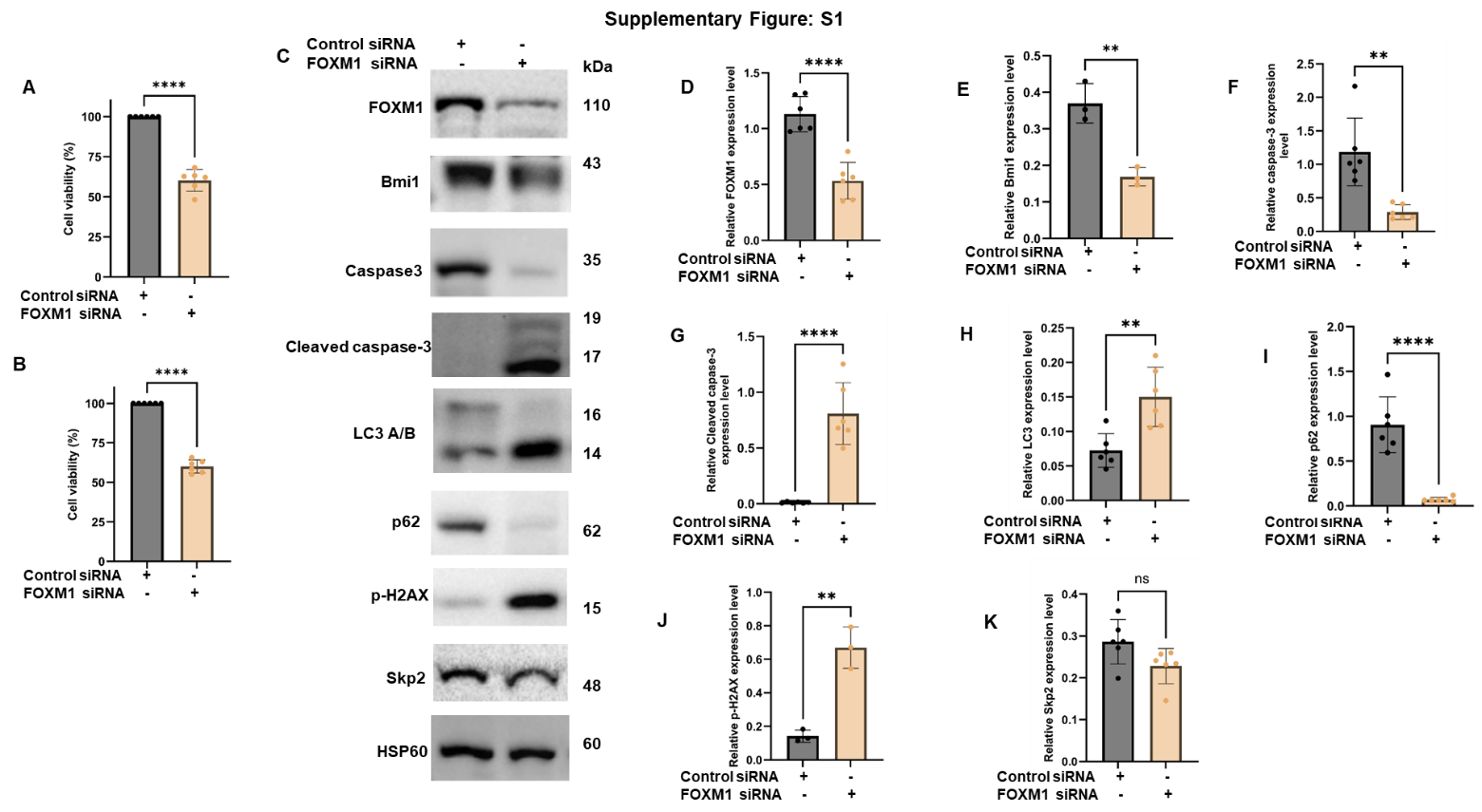


S1. siRNA-mediated FOXM1 knockdown inhibits the proliferation of CTCL cells through programmed cell death. (A, B) Cell viability analysis of FOXM1 knockdown HH and H9 cells compared to the control group. Data is presented as mean ± SD (n=6). (C) FOXM1 knockdown cells (H9) were lysed, and cell lysates were prepared. Western blot analysis of different proteins (FOXM1, Bmi1, caspase-3, cleaved caspase-3, LC3 A/B, p62, p-H2AX, and Skp2) was performed. (D-K) Relative quantification of the band intensities of FOXM1, Bmi1, caspase-3, cleaved caspase-3, LC3, p62, p-H2AX, and Skp2 presented as mean ± SD (n=3, n=6). The band intensities were normalized with the respective loading controls and quantified using Image Lab software. *P < 0.05, **P<0.01, and ****P < 0.0001 shows level of significance between groups.


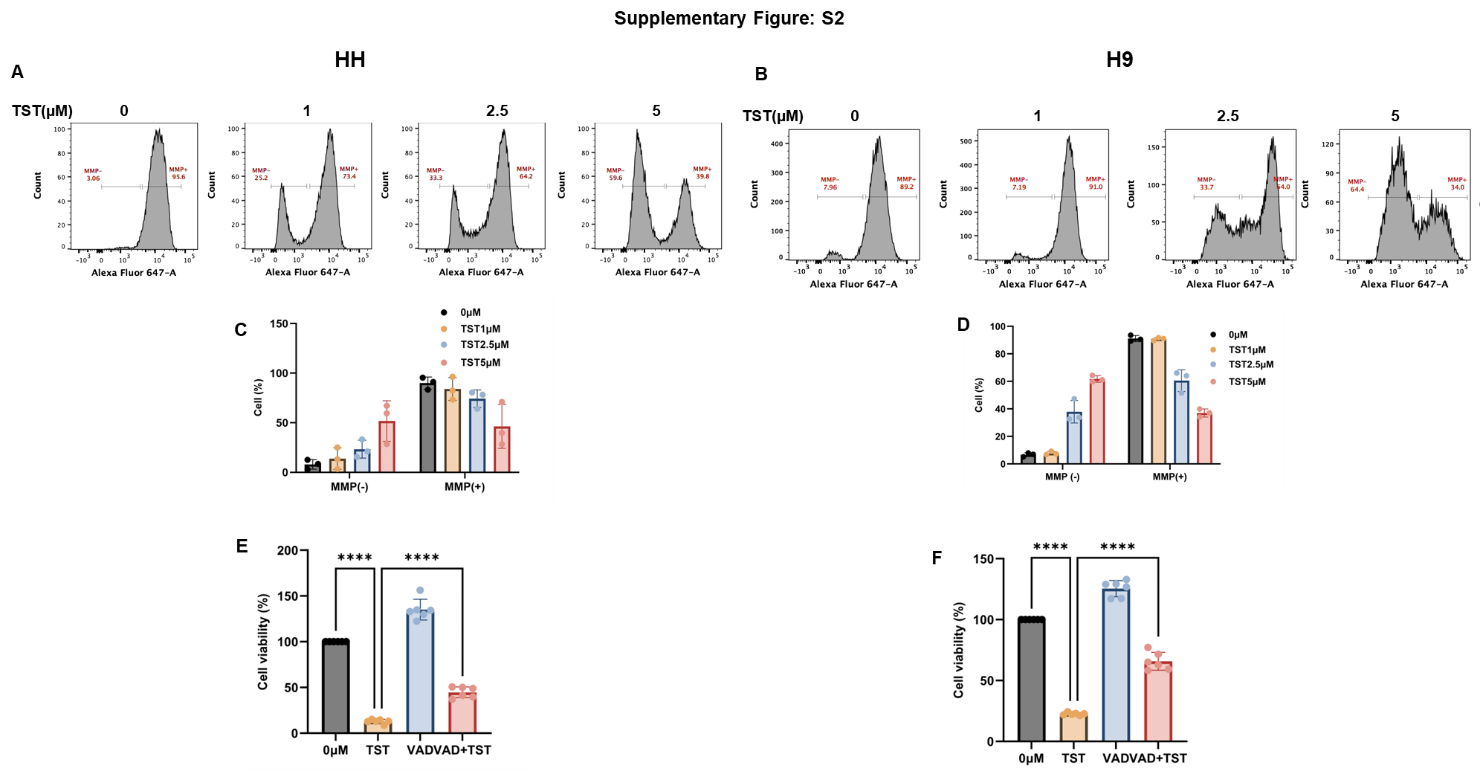


S2. Thiostrepton (TST) causes mitochondrial membrane potential (MMP) depletion in CTCL cells. (A, C; B, D) HH and H9 cells were treated with the indicated concentrations of TST for 24 hours and analyzed by flow cytometry. The graphs show a noticeable increase in MMP loss in TST-treated cells, presented as mean ± SD (n = 3). (E, F) HH and H9 cells were treated with TST (5μM) and pan caspase inhibitor z-VAD-FMK(40μM), alone or in combination. Cell viability was determined by using a CCK-8 kit assay. Data is presented as mean ± SD (n=6).


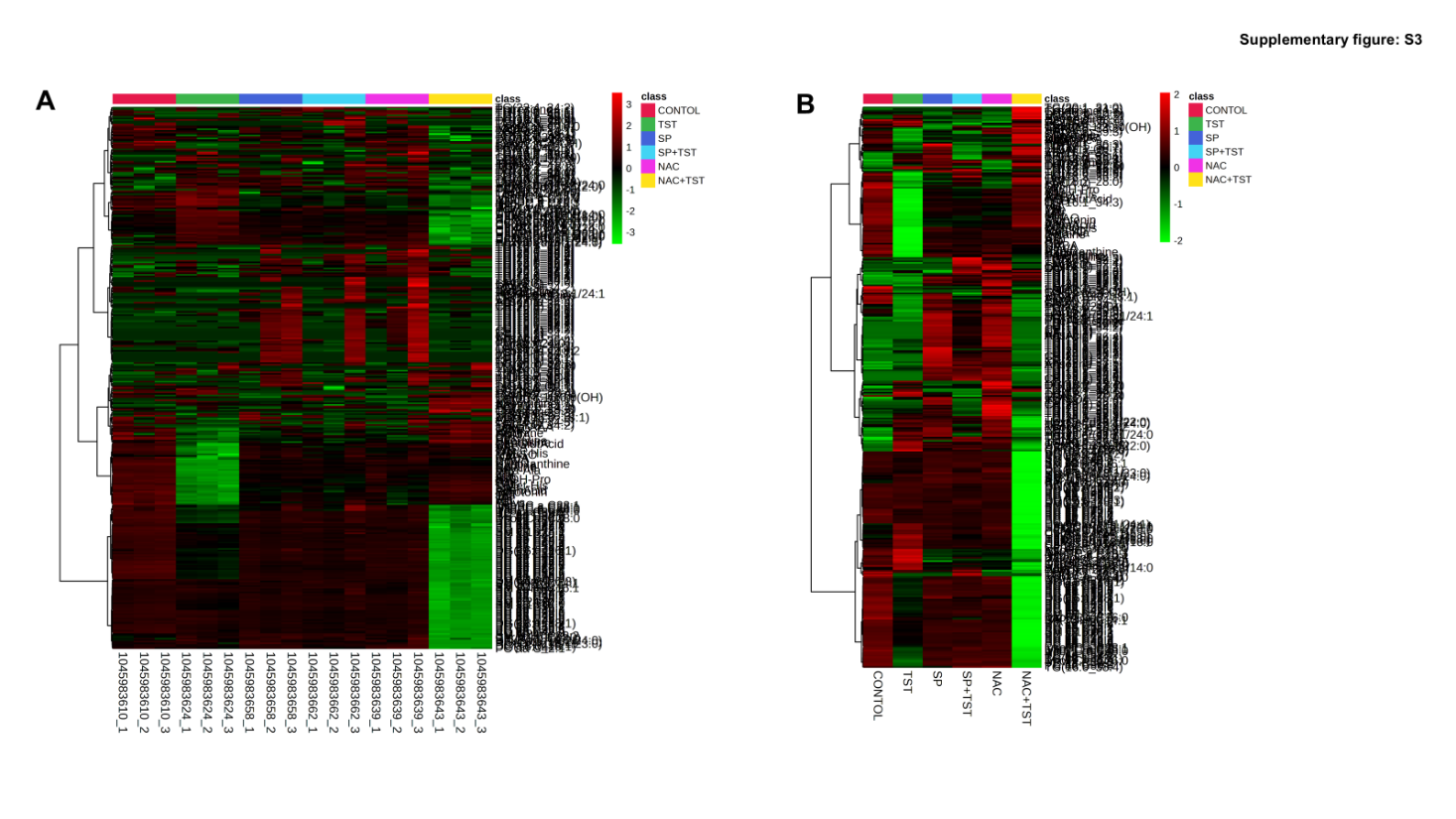


S3 (A, B,) Heatmap with hierarchical clustering showing metabolite abundance for individual samples (A) and group-wise averages (B). Red indicates increased metabolite levels and green indicates decreased levels, with samples shown in columns and metabolites in rows. Cells were treated with TST (5µM), SP600125 (10µM), and NAC (6mM), alone or in combination, followed by metabolomics analysis as described in materials and methods. The metabolomics data analysis and visualization were performed using MetaboAnalyst 6.0 (https://www.metaboanalyst.ca/).


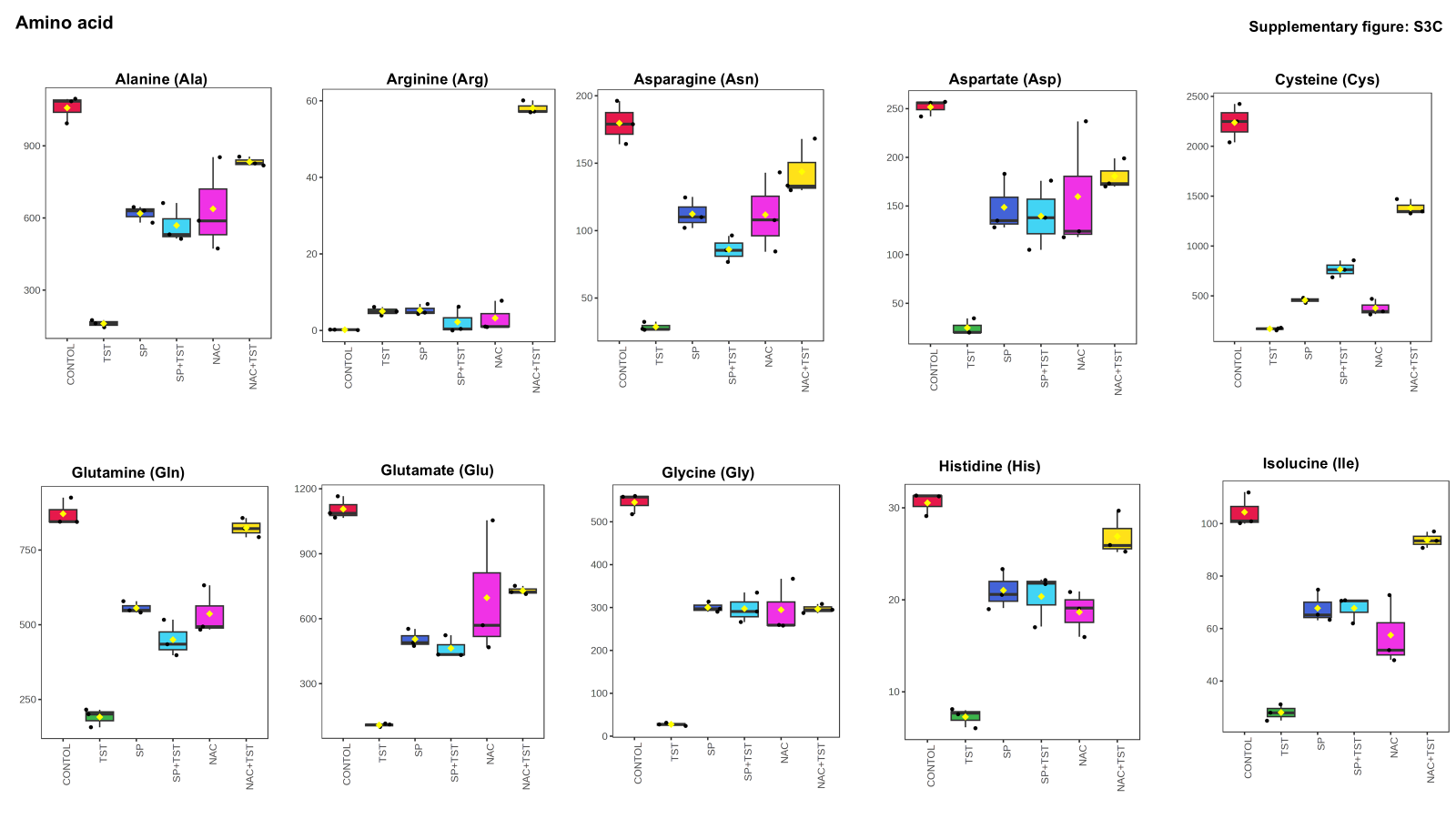


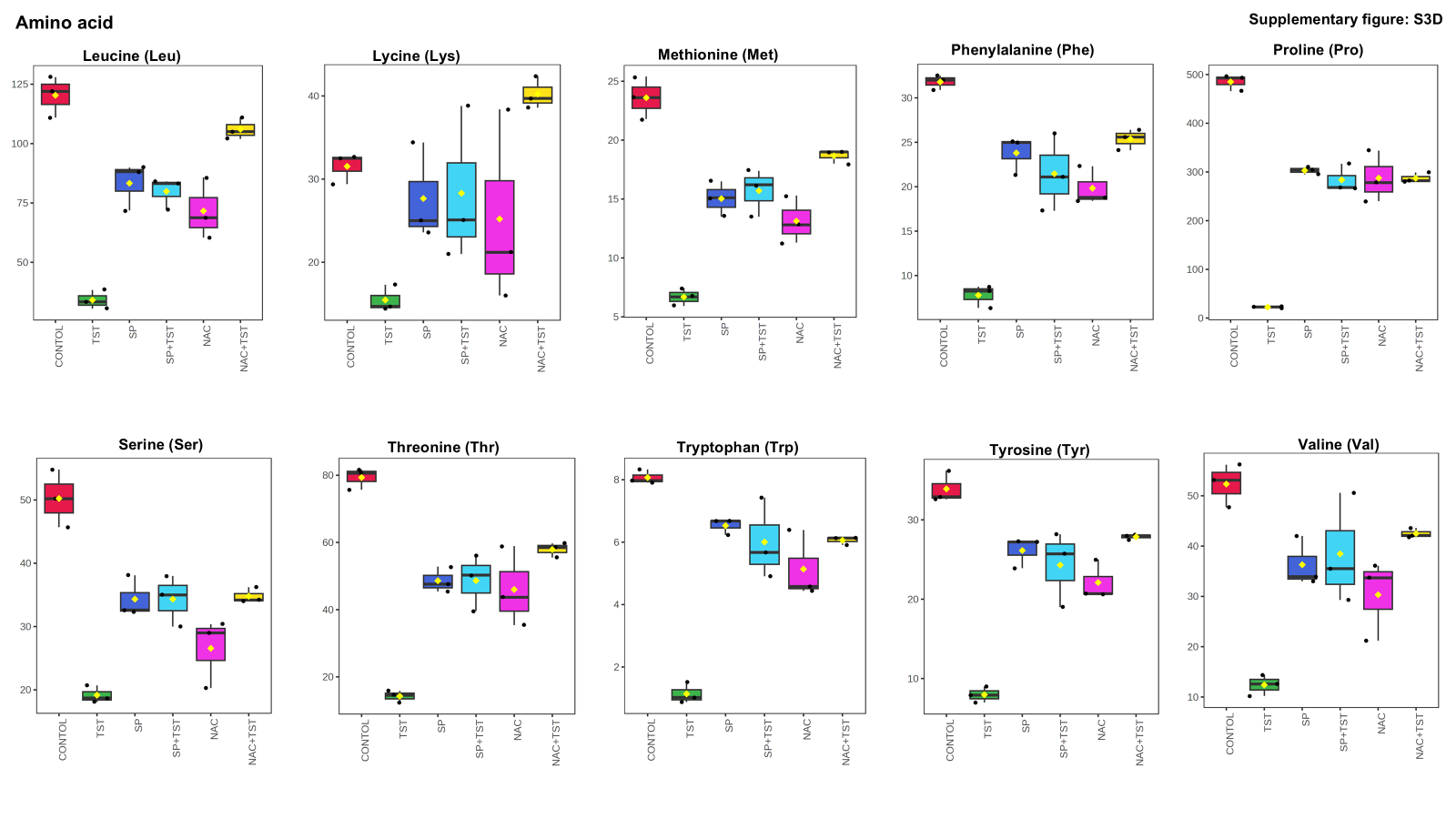


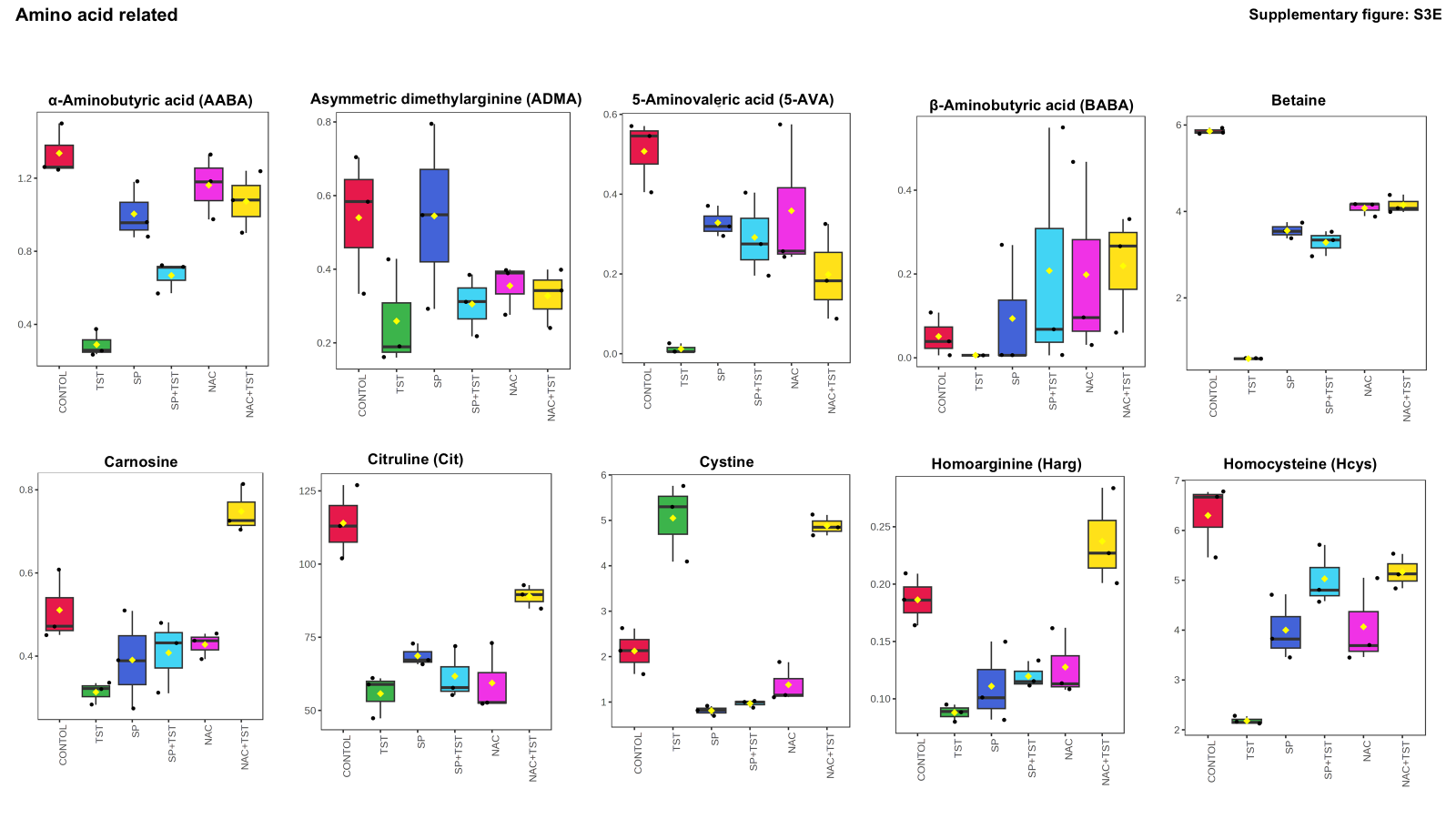


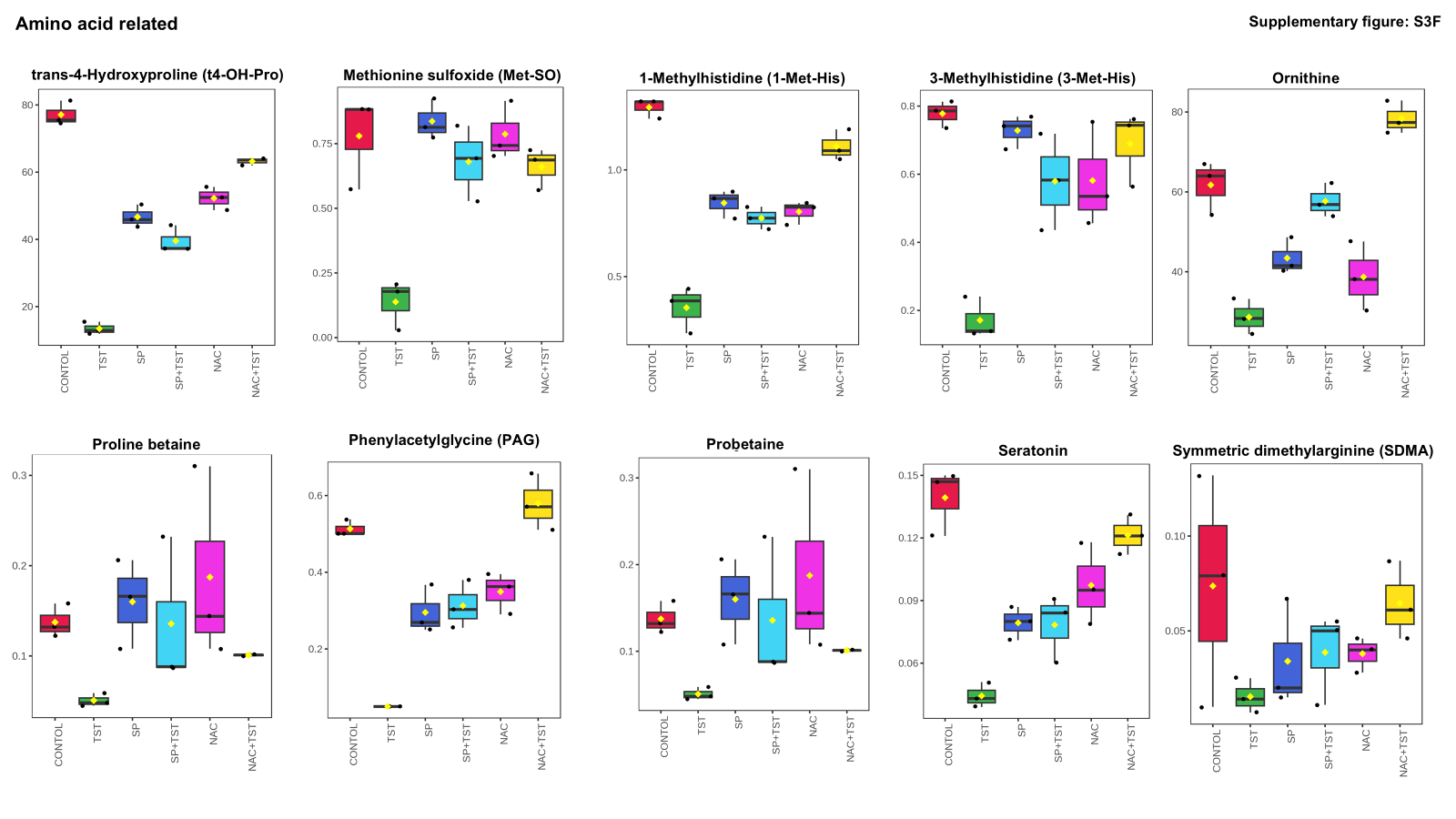

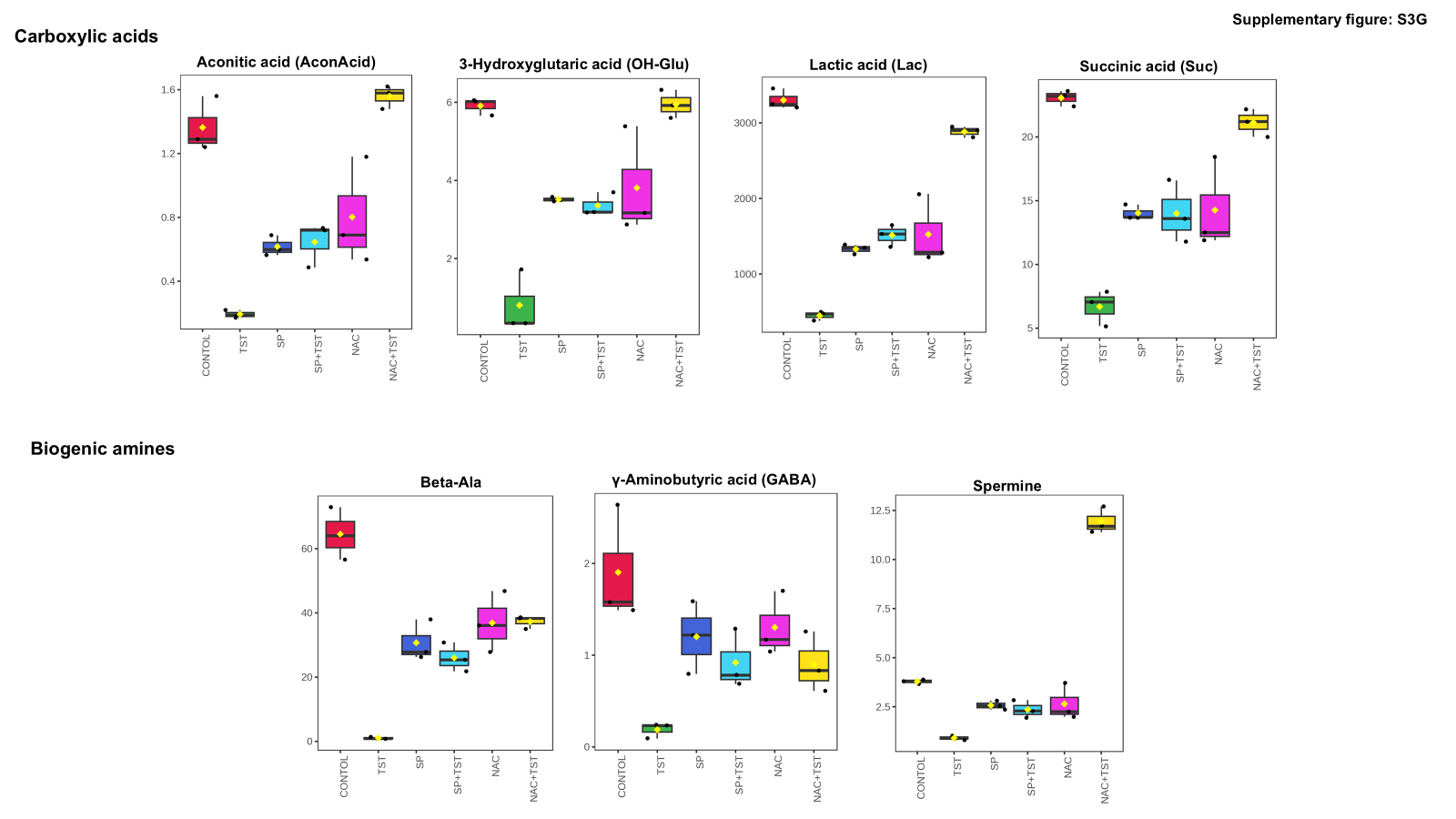


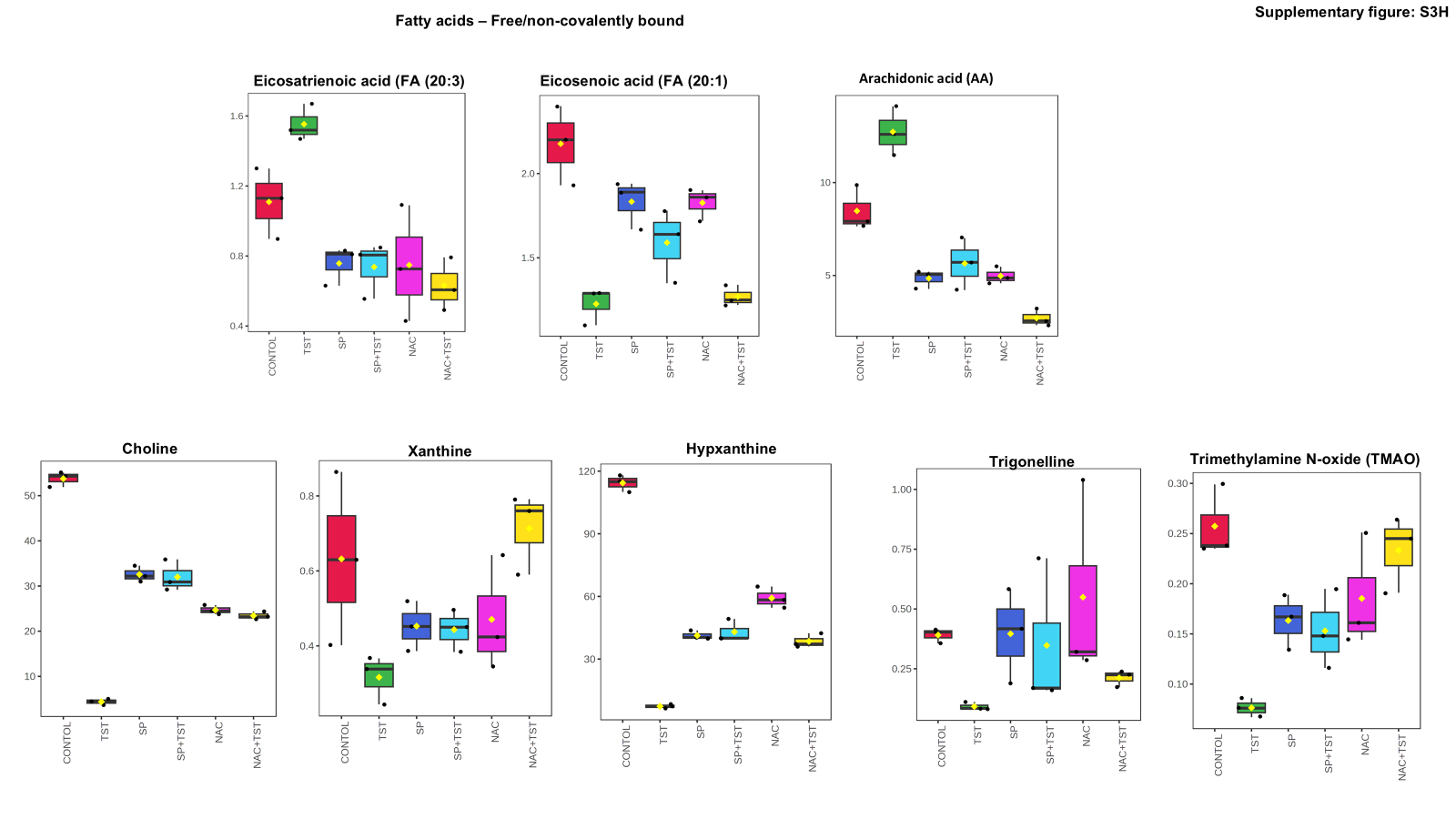


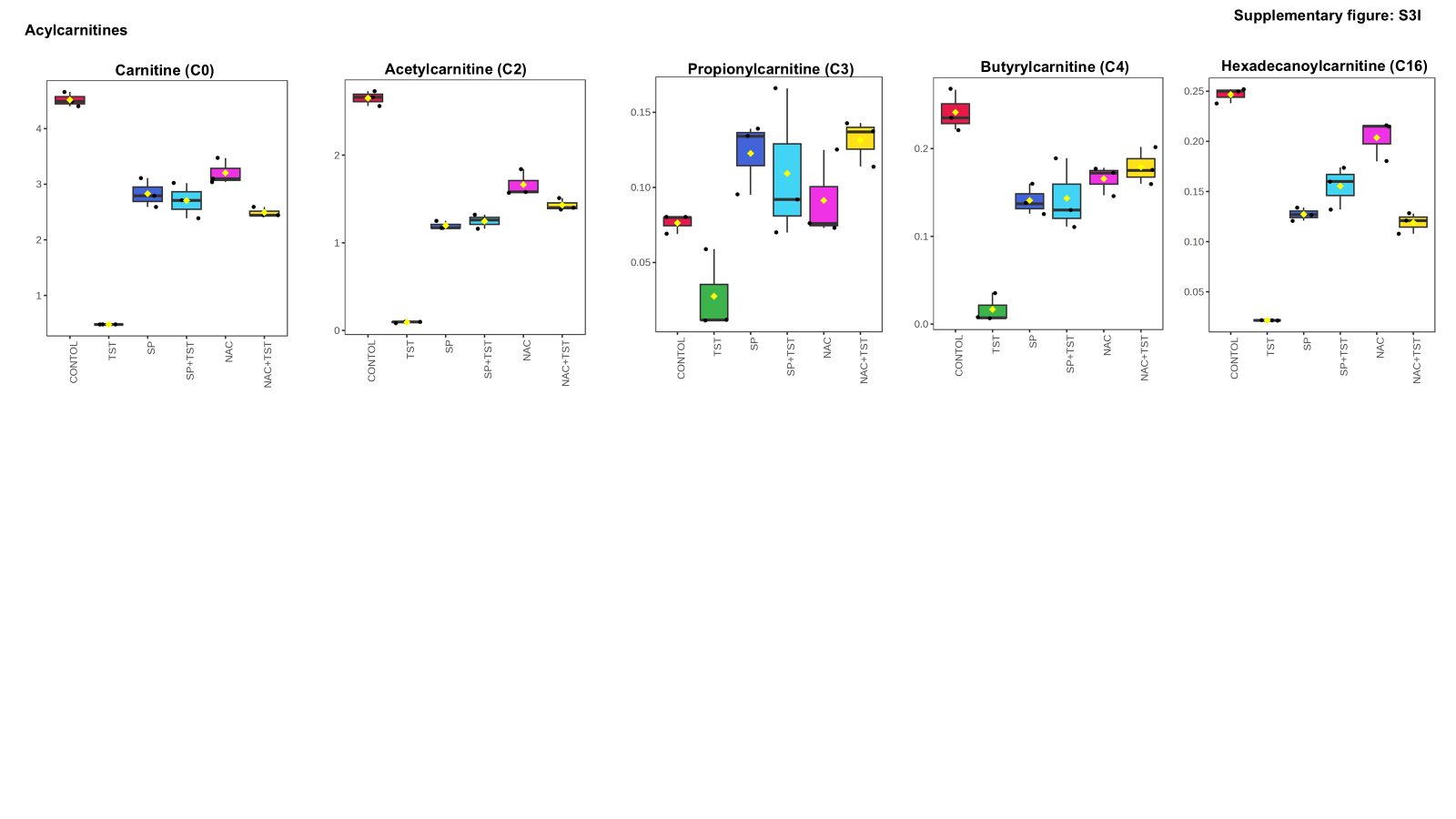


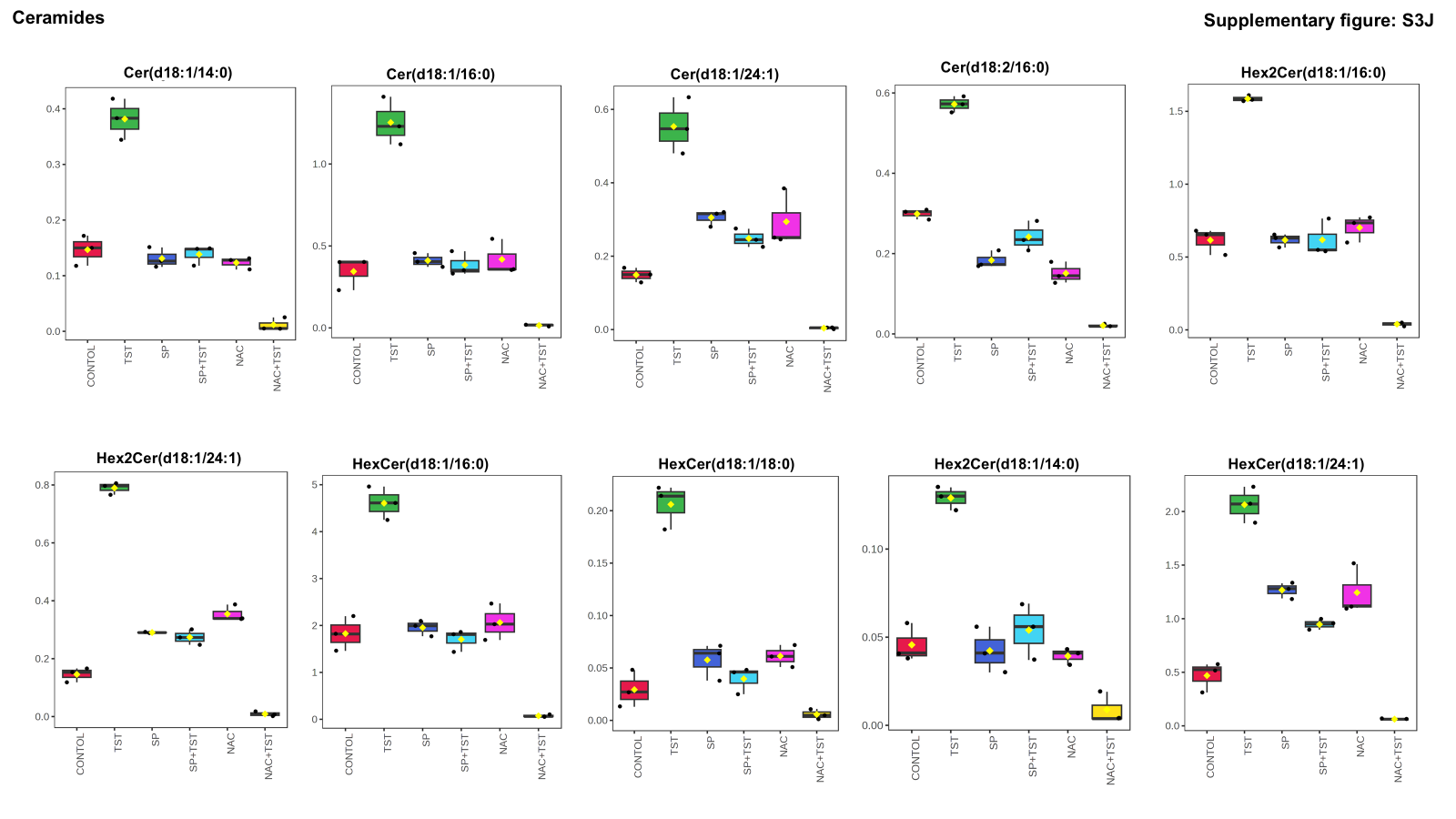


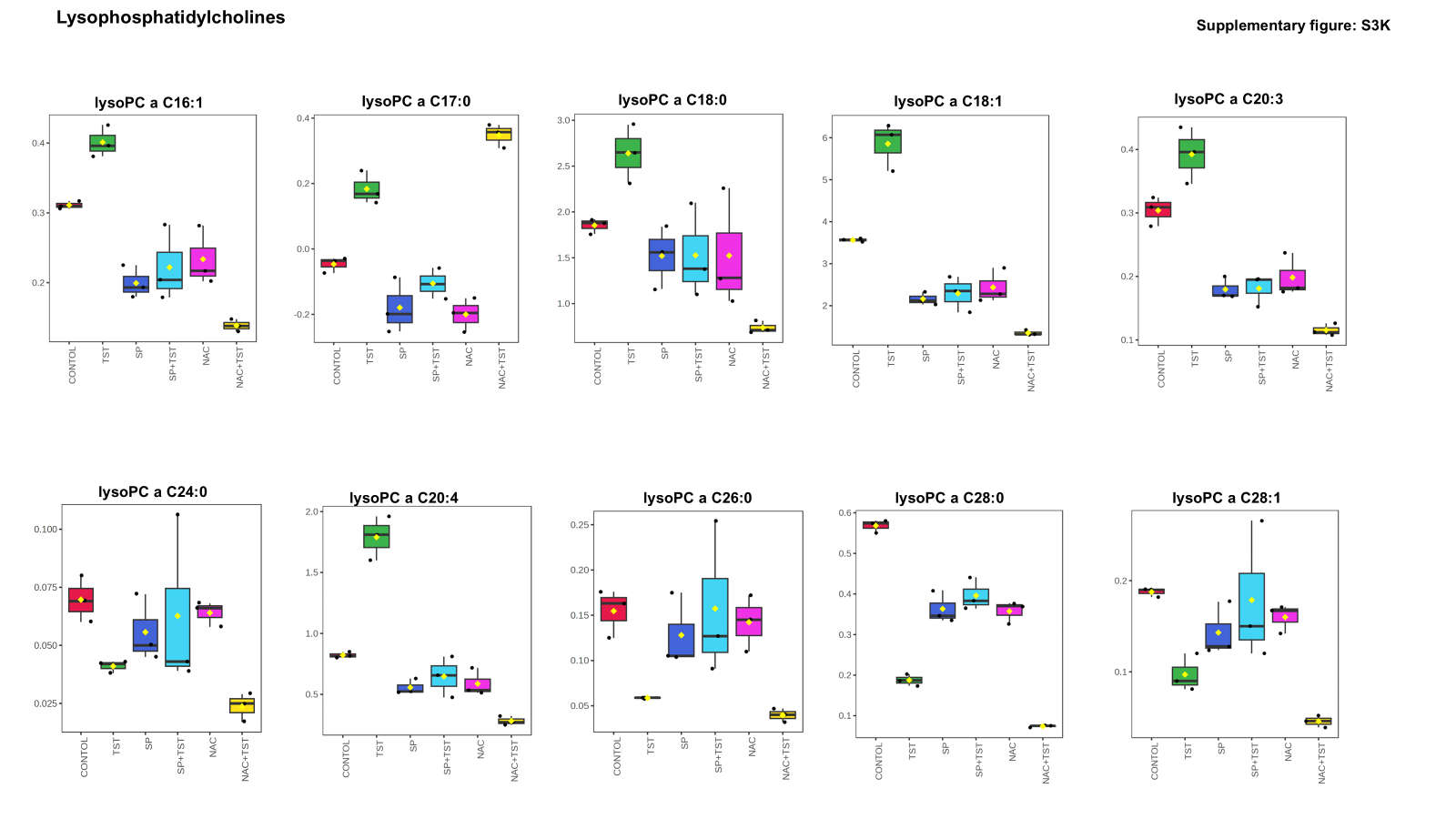


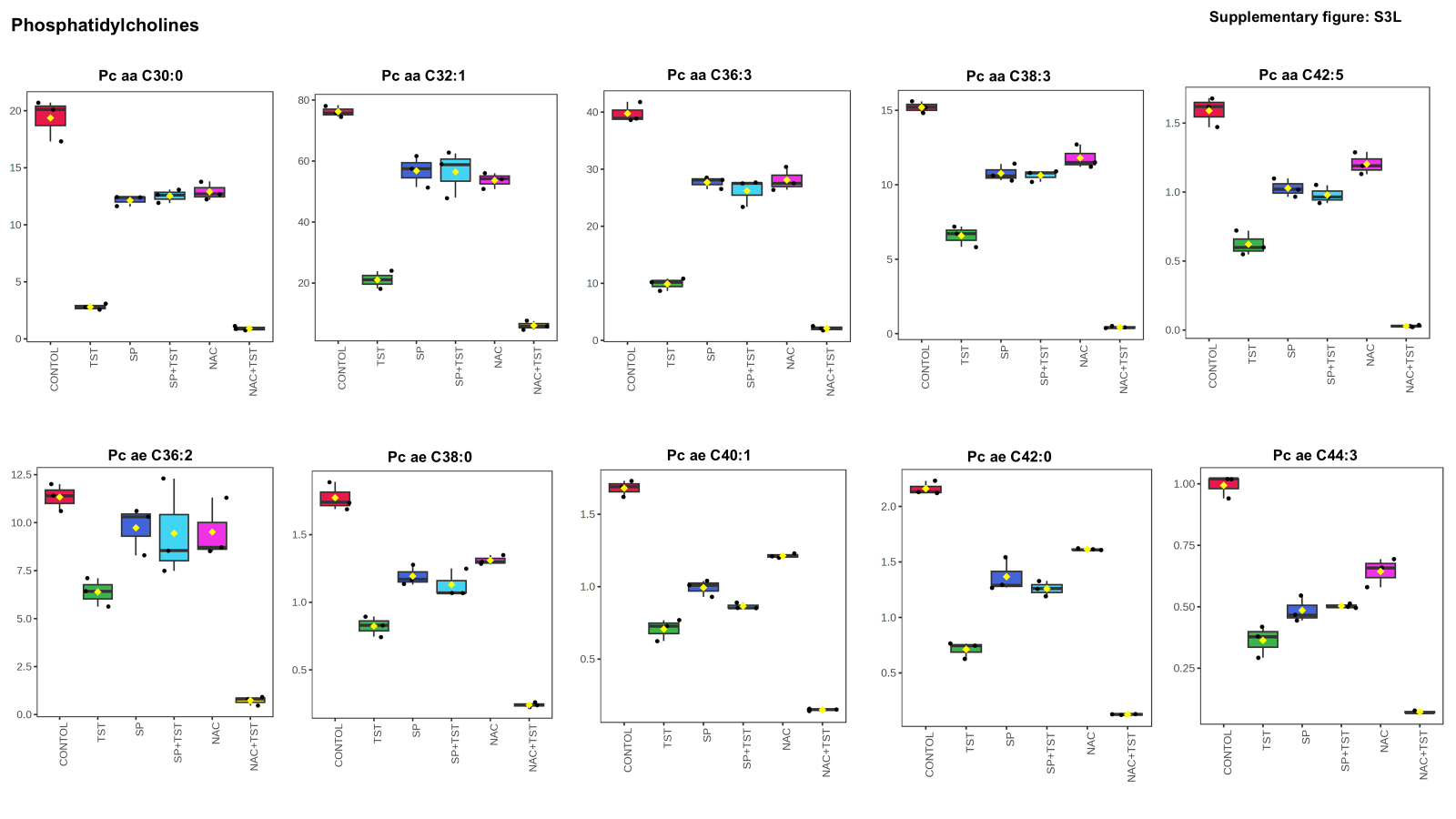

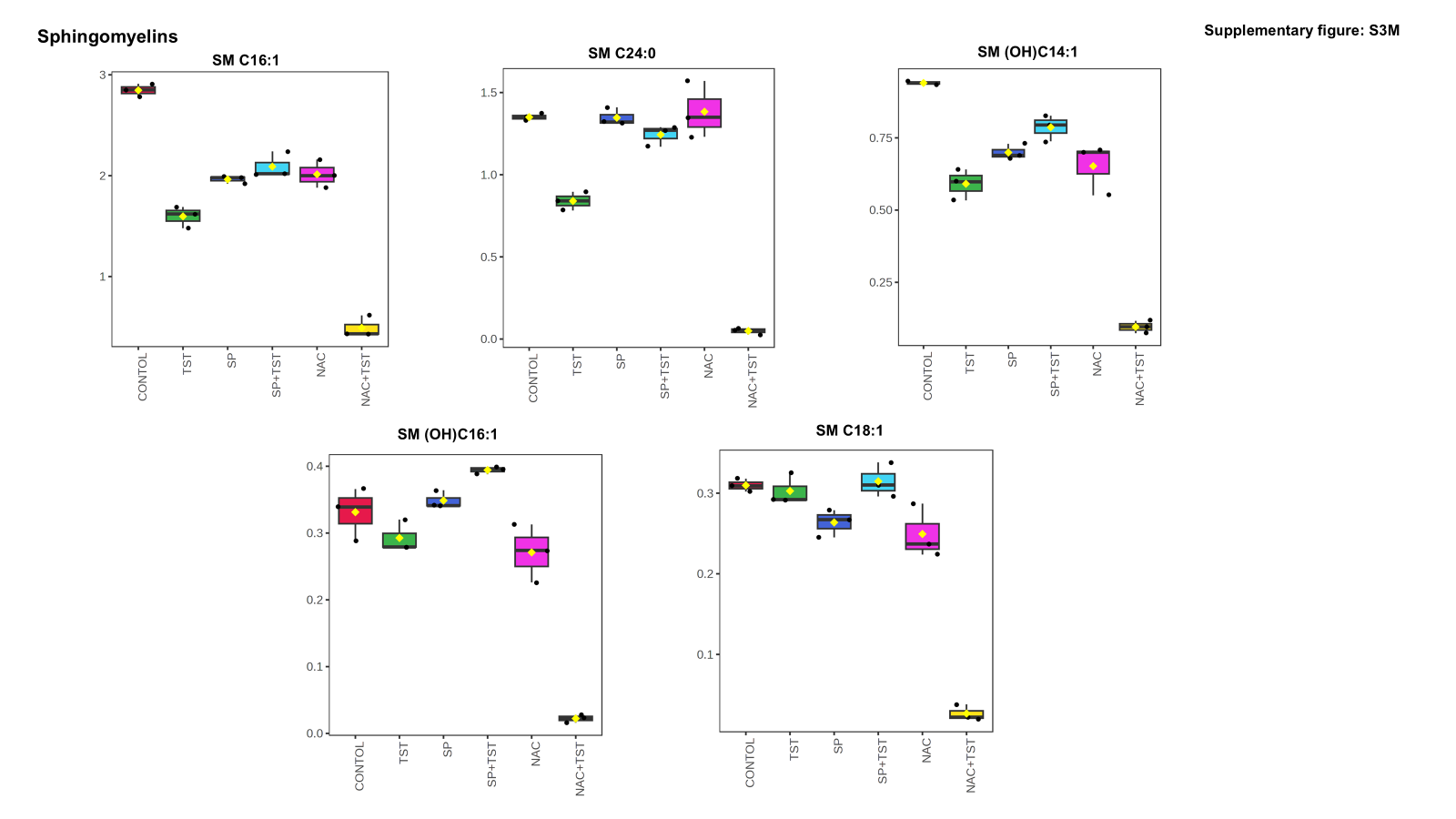
(S3C-S3M) Box plot analysis showing the relative abundance of metabolites in CTCL cells. Cells were treated with TST (5µM), SP600125 (10µM), and NAC (6mM), alone or in combination, followed by metabolomics analysis as described in materials and methods. The metabolomics data analysis and visualization were performed using MetaboAnalyst 6.0 (https://www.metaboanalyst.ca/).


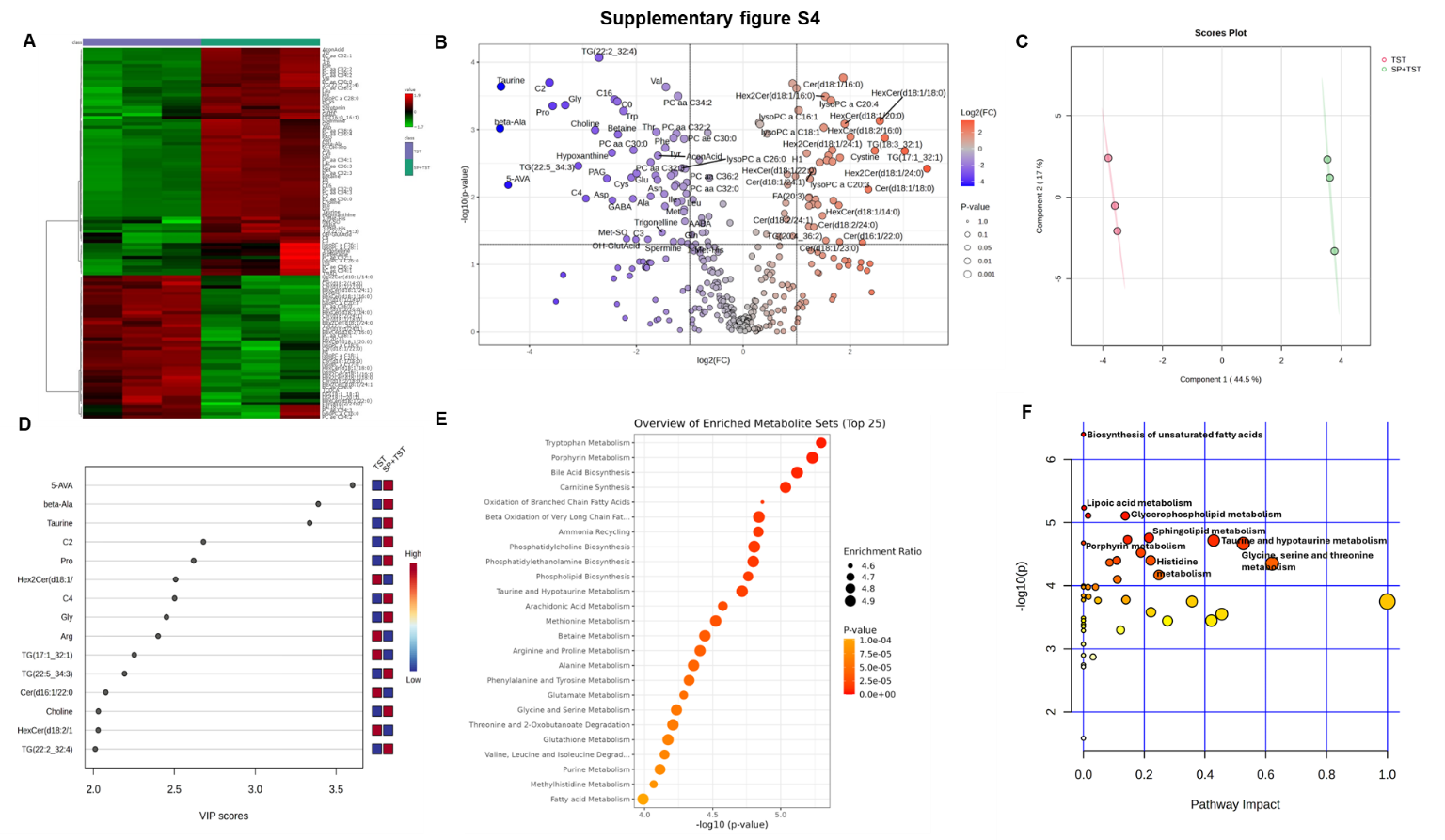


S4. Metabolomic profiling of TST vs SP+TST-treated cells. (A) Heatmap with hierarchical clustering of significantly altered metabolites (p<0.05) identified between TST and SP+TST-treated cells. Red indicates an increased level, and green represents a low level, with samples in columns and metabolites in rows. (B) Volcano plot showing differentially expressed metabolites. Red-labelled dots depict up-regulated metabolites, while blue-labeled dots represent down-regulated metabolites. The fold change and the raw p-value cutoffs were set at 2.0 and 0.05, respectively. (C) PLS-DA of the metabolomics data from TST and SP+TST-treated cells. (D) Variable importance in projection (VIP) score plot of the top 15 important features based on the PLS-DA model. The colored scale on the right (red and blue) indicates increased and decreased metabolite levels in the TST and SP+TST group. (E) Functional analysis of the significant features in TST and SP+TST-treated cells using MetaboAnalyst 6.0 (https://www.metaboanalyst.ca/). Quantitative enrichment analysis (QEA) overview of the top 25 metabolic pathways based on SMPDB. (F) Pathway analysis based on the KEGG database.


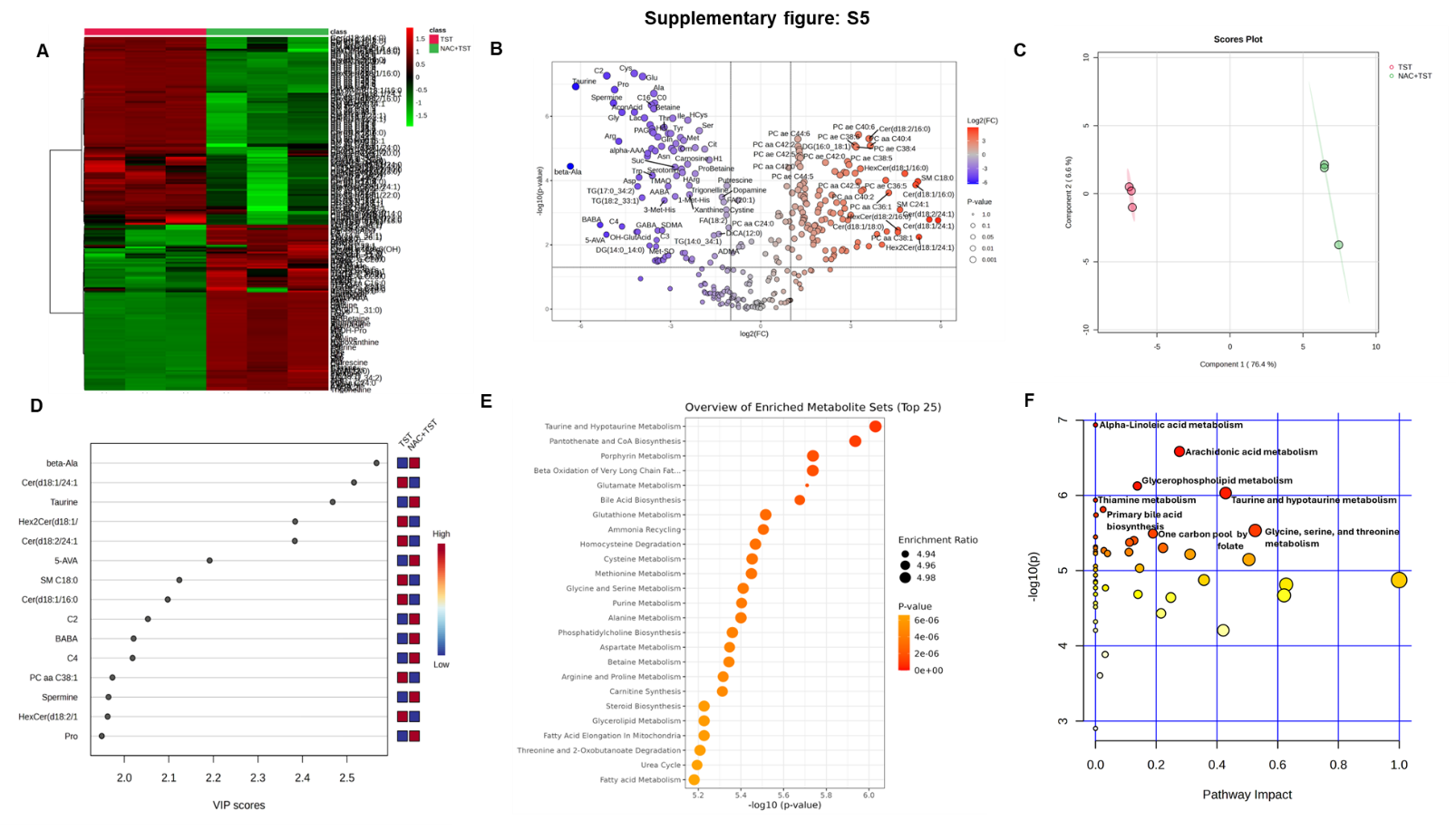


S5. Metabolomic profiling of TST vs NAC+TST-treated cells. (A) Heatmap with hierarchical clustering of the significantly altered metabolites (p<0.05) identified between TST and NAC+TST-treated cells. Red indicates an increased level and green represents a decreased level, with samples in columns and metabolites in rows. (B) Volcano plot showing differentially expressed metabolites. Red-labeled dots depict up-regulated metabolites, and blue-labeled dots represent down-regulated metabolites. The fold change and the raw p-value cutoffs were set at 2.0 and 0.05, respectively. (C) PLS-DA of the metabolomics data from TST and NAC+TST-treated cells. (D) Variable importance in projection (VIP) score plot showing the top 15 important features based PLS-DA model. The colored scale on the right (red and blue) indicates increased and decreased metabolite levels in the TST and NAC+TST groups. (E) Functional analysis of the significant features in TST and NAC+TST-treated cells using MetaboAnalyst 6.0 (https://www.metaboanalyst.ca/). Quantitative enrichment analysis (QEA) overview showing the top 25 metabolic pathways based on SMPDB. (F) Pathway analysis based on the KEGG database.
