# Supplementary material for: Targeting FOXM1 regulates metabolic signatures through ROS-dependent JNK/Bmi1/Skp2 axis in human cutaneous T-cell lymphoma
Source: Cell Death Dis. 2026 Jan 7;17(1):170. doi: 10.1038/s41419-025-08389-z (PMC12876963; doi:10.1038/s41419-025-08389-z)
Supplement: Supplementary file 3 — Supplementary Tables [file 41419_2025_8389_MOESM3_ESM.pdf]

Metabolite	f.value	p.value	p-value (-log10)	FDR
PC aa C40:1	1170	1.08E-15	14.968	3.77E-13
C2	940.08	3.99E-15	14.4	6.97E-13
PC aa C40:4	834.23	8.14E-15	14.089	9.50E-13
Betaine	792.17	1.11E-14	13.955	9.71E-13
PC ae C42:0	726	1.87E-14	13.729	1.31E-12
PC ae C40:6	672.03	2.96E-14	13.528	1.69E-12
PC ae C38:4	654.57	3.47E-14	13.46	1.69E-12
PC ae C40:1	643.14	3.85E-14	13.414	1.69E-12
PC aa C40:6	595.11	6.13E-14	13.213	2.16E-12
PC aa C38:3	593.48	6.23E-14	13.206	2.16E-12
PC ae C38:6	584.85	6.80E-14	13.168	2.16E-12
PC aa C38:4	525.67	1.28E-13	12.891	3.75E-12
PC aa C40:5	516.92	1.42E-13	12.848	3.82E-12
PC aa C38:6	461.54	2.79E-13	12.554	6.92E-12
PC ae C42:2	456.89	2.97E-13	12.528	6.92E-12
PC aa C42:5	441.29	3.65E-13	12.438	7.98E-12
PC ae C38:5	425.07	4.56E-13	12.341	9.15E-12
PC ae C40:5	422.85	4.70E-13	12.328	9.15E-12
PC ae C40:4	412.41	5.46E-13	12.263	9.78E-12
PC aa C38:5	410.8	5.59E-13	12.253	9.78E-12
DG(16:0_18:1)	407.22	5.89E-13	12.23	9.81E-12
Pro	383.27	8.45E-13	12.073	1.32E-11
C0	381.05	8.74E-13	12.058	1.32E-11
PC ae C42:1	379.05	9.02E-13	12.045	1.32E-11
PC ae C36:4	372.14	1.01E-12	11.997	1.37E-11
PC aa C36:6	371.45	1.02E-12	11.992	1.37E-11
PC aa C36:4	338.13	1.78E-12	11.749	2.31E-11
PC aa C36:3	327.41	2.16E-12	11.666	2.62E-11
C16	326.1	2.21E-12	11.656	2.62E-11
PC aa C30:0	325.22	2.24E-12	11.649	2.62E-11
PC ae C38:0	317.68	2.58E-12	11.588	2.91E-11
Choline	310.91	2.93E-12	11.533	3.21E-11
PC aa C34:1	303.14	3.41E-12	11.467	3.62E-11
PC ae C42:5	298	3.77E-12	11.423	3.89E-11
Hypoxanthine	292.22	4.24E-12	11.373	4.24E-11
PC aa C36:0	280.84	5.37E-12	11.27	5.22E-11
PC aa C38:0	279.26	5.55E-12	11.256	5.25E-11
lysoPC a C28:0	275.87	5.97E-12	11.224	5.45E-11
PC ae C44:6	275.04	6.08E-12	11.216	5.45E-11
PC aa C32:3	264.65	7.64E-12	11.117	6.68E-11
Cer(d18:2/16:0)	263.54	7.83E-12	11.106	6.68E-11
PC aa C42:0	258.93	8.69E-12	11.061	7.25E-11

Gly	256.49	9.20E-12	11.036	7.48E-11
PC ae C44:5	248.16	1.12E-11	10.951	8.77E-11
PC aa C36:5	247.84	1.13E-11	10.948	8.77E-11
PC ae C40:2	242.66	1.28E-11	10.894	9.66E-11
PC aa C36:1	242.05	1.30E-11	10.887	9.66E-11
PC ae C44:3	238.77	1.41E-11	10.852	1.03E-10
PC ae C38:3	237.52	1.45E-11	10.838	1.04E-10
PC aa C40:2	231.55	1.69E-11	10.773	1.18E-10
PC aa C34:4	213.84	2.70E-11	10.568	1.85E-10
Cys	212.15	2.83E-11	10.548	1.91E-10
PC aa C40:3	211.01	2.92E-11	10.534	1.93E-10
PC aa C34:3	204.97	3.47E-11	10.46	2.25E-10
PC ae C38:1	200.04	4.01E-11	10.397	2.55E-10
PC ae C40:3	193.85	4.83E-11	10.316	2.99E-10
PC ae C36:5	193.62	4.86E-11	10.313	2.99E-10
SM C18:0	189.08	5.59E-11	10.252	3.38E-10
HexCer(d18:1/24:1)	187.48	5.88E-11	10.23	3.49E-10
PC ae C36:1	185.02	6.36E-11	10.197	3.71E-10
PC aa C32:0	182.4	6.92E-11	10.16	3.97E-10
PC ae C38:2	174.51	8.98E-11	10.047	5.07E-10
t4-OH-Pro	171.13	1.01E-10	9.9966	5.55E-10
SM (OH) C16:1	170.93	1.01E-10	9.9936	5.55E-10
HexCer(d18:1/16:0)	166.63	1.18E-10	9.9282	6.28E-10
PC aa C32:1	166.5	1.18E-10	9.9263	6.28E-10
SM C16:0	162.57	1.36E-10	9.8651	7.13E-10
beta-Ala	159.2	1.54E-10	9.8115	7.94E-10
PC aa C42:2	152.24	2.01E-10	9.6972	1.02E-09
Cer(d18:2/18:0)	151.31	2.08E-10	9.6814	1.04E-09
SM (OH) C14:1	150.83	2.12E-10	9.6734	1.05E-09
PC ae C42:3	143.86	2.80E-10	9.5525	1.36E-09
Hex2Cer(d18:1/16:0)	135.69	3.95E-10	9.4033	1.89E-09
SM C24:1	131.66	4.72E-10	9.3263	2.23E-09
SM C24:0	130.88	4.88E-10	9.3112	2.28E-09
SM C16:1	121.84	7.43E-10	9.129	3.42E-09
PC ae C30:0	117.68	9.11E-10	9.0407	4.13E-09
SM C18:1	117.47	9.21E-10	9.036	4.13E-09
PC ae C34:0	114.99	1.04E-09	8.9818	4.62E-09
PC ae C34:1	113.71	1.11E-09	8.9533	4.87E-09
PAG	113.01	1.15E-09	8.9378	4.99E-09
PC ae C42:4	109.68	1.37E-09	8.8617	5.87E-09
DG(16:0_20:3)	107.6	1.54E-09	8.8131	6.49E-09
Cer(d18:1/16:0)	103.97	1.88E-09	8.726	7.83E-09
DG(18:1_18:1)	102.59	2.03E-09	8.6924	8.31E-09

SM (OH) C22:2	102.5	2.04E-09	8.6899	8.31E-09
Cer(d18:0/24:1)	101.21	2.20E-09	8.658	8.84E-09
PC aa C28:1	97.602	2.72E-09	8.5661	1.08E-08
PC ae C32:2	91.229	4.02E-09	8.3954	1.58E-08
PC ae C36:3	88.371	4.84E-09	8.3151	1.88E-08
PC aa C36:2	83.868	6.56E-09	8.1833	2.52E-08
Lac	79.415	8.99E-09	8.0461	3.42E-08
PC ae C36:2	79.186	9.14E-09	8.0388	3.43E-08
Cer(d18:1/24:0)	79.076	9.22E-09	8.0353	3.43E-08
PC ae C36:0	78.384	9.70E-09	8.0133	3.57E-08
PC ae C30:1	78.228	9.81E-09	8.0083	3.58E-08
Gln	74.687	1.28E-08	7.892	4.63E-08
Spermine	73.905	1.36E-08	7.8656	4.87E-08
PC aa C34:2	65.44	2.74E-08	7.5615	9.70E-08
PC aa C42:1	65.167	2.81E-08	7.5511	9.84E-08
Ala	62.891	3.45E-08	7.4625	1.19E-07
PC aa C32:2	61.506	3.92E-08	7.4071	1.34E-07
Tyr	59.493	4.74E-08	7.3244	1.61E-07
Cer(d18:1/24:1)	58.902	5.02E-08	7.2996	1.69E-07
His	58.381	5.28E-08	7.2775	1.76E-07
DG(16:0_16:1)	58.206	5.37E-08	7.2701	1.77E-07
Cer(d18:2/24:1)	57.485	5.77E-08	7.2392	1.89E-07
Cystine	56.22	6.55E-08	7.184	2.12E-07
Trp	55.631	6.95E-08	7.158	2.19E-07
lysoPC a C18:1	55.607	6.97E-08	7.1569	2.19E-07
Asn	55.586	6.98E-08	7.156	2.19E-07
Glu	55.566	7.00E-08	7.1551	2.19E-07
Hex2Cer(d18:1/24:0)	52.646	9.51E-08	7.0217	2.95E-07
Thr	51.284	1.10E-07	6.957	3.39E-07
PC ae C32:1	49.826	1.30E-07	6.886	3.96E-07
Ile	49.449	1.36E-07	6.8673	4.10E-07
PC aa C42:4	48.518	1.51E-07	6.8206	4.52E-07
Leu	47.542	1.70E-07	6.7707	5.03E-07
Met	47.214	1.76E-07	6.7537	5.19E-07
Phe	46.787	1.86E-07	6.7314	5.41E-07
Hex2Cer(d18:1/18:0)	46.348	1.96E-07	6.7083	5.66E-07
PC ae C34:2	45.52	2.17E-07	6.6642	6.22E-07
lysoPC a C20:4	44.787	2.37E-07	6.6245	6.76E-07
PC aa C42:6	44.541	2.45E-07	6.611	6.91E-07
PC ae C30:2	44.316	2.52E-07	6.5986	7.06E-07
PC ae C34:3	43.21	2.90E-07	6.5369	8.07E-07
DG(16:0_18:2)	41.348	3.72E-07	6.4296	1.02E-06
lysoPC a C20:3	40.905	3.95E-07	6.4035	1.08E-06

AconAcid	38.607	5.46E-07	6.2631	1.48E-06
AABA	36.92	7.00E-07	6.1552	1.88E-06
AA	35.538	8.64E-07	6.0632	2.31E-06
Asp	33.89	1.12E-06	5.9492	2.98E-06
Spermidine	31.827	1.59E-06	5.7991	4.18E-06
Hex2Cer(d18:1/24:1)	31.601	1.65E-06	5.7821	4.31E-06
HexCer(d18:1/23:0)	31.555	1.66E-06	5.7786	4.32E-06
Cer(d18:1/14:0)	30.913	1.86E-06	5.7297	4.80E-06
HexCer(d18:1/24:0)	29.273	2.51E-06	5.6004	6.41E-06
1-Met-His	28.883	2.70E-06	5.5686	6.85E-06
PC aa C38:1	28.353	2.99E-06	5.5249	7.52E-06
H1	27.376	3.61E-06	5.4424	9.03E-06
HexCer(d18:2/24:0)	26.04	4.73E-06	5.325	1.17E-05
Suc	25.615	5.17E-06	5.2865	1.27E-05
HCys	24.165	7.07E-06	5.1508	1.73E-05
5-AVA	23.222	8.74E-06	5.0587	2.12E-05
Orn	23.094	9.00E-06	5.0459	2.17E-05
lysoPC a C16:1	22.717	9.82E-06	5.008	2.35E-05
3-Met-His	22.199	1.11E-05	4.9549	2.64E-05
Ser	21.796	1.22E-05	4.9128	2.89E-05
C4	21.622	1.28E-05	4.8945	3.00E-05
Serotonin	21.432	1.34E-05	4.8743	3.11E-05
Val	21.414	1.34E-05	4.8723	3.11E-05
PC ae C44:4	20.817	1.56E-05	4.8077	3.59E-05
HexCer(d18:1/22:0)	20.659	1.62E-05	4.7904	3.71E-05
Putrescine	18.453	2.92E-05	4.5351	6.63E-05
lysoPC a C28:1	18.226	3.11E-05	4.5073	7.02E-05
FA(20:1)	17.45	3.89E-05	4.4103	8.72E-05
Hex2Cer(d18:1/22:0)	15.831	6.38E-05	4.1954	0.00014217
Cit	15.242	7.72E-05	4.1125	0.00017097
Hex2Cer(d18:1/14:0)	15.118	8.04E-05	4.0948	0.00017696
GABA	14.827	8.86E-05	4.0526	0.0001938
HexCer(d18:1/20:0)	14.618	9.51E-05	4.0218	0.00020673
lysoPC a C17:0	14.169	0.00011103	3.9546	0.00023987
HexCer(d18:1/18:0)	14.118	0.00011302	3.9468	0.00024269
TG(20:4_34:1)	14.087	0.00011428	3.942	0.00024389
TMAO	13.487	0.00014156	3.8491	0.00030028
OH-GlutAcid	13.259	0.00015392	3.8127	0.00032454
lysoPC a C26:0	12.134	0.00023648	3.6262	0.00049562
SM (OH) C22:1	12.097	0.00024004	3.6197	0.00050007
HArg	11.645	0.00028791	3.5407	0.00059626
TG(20:1_31:0)	11.453	0.0003116	3.5064	0.00064152
C18	10.906	0.00039252	3.4061	0.00080341

TG(22:5_34:3)	10.452	0.00047873	3.3199	0.00097415
lysoPC a C18:0	8.9081	0.00099201	3.0035	0.002007
Cer(d18:2/22:0)	8.82	0.0010369	2.9842	0.0020858
Met-SO	8.79	0.0010528	2.9776	0.0021056
DHA	8.5399	0.0011964	2.9221	0.0023793
Carnosine	8.3175	0.0013437	2.8717	0.002657
Arg	7.8532	0.0017246	2.7633	0.003391
DG(16:0_20:4)	7.3106	0.0023397	2.6308	0.0045749
lysoPC a C26:1	7.2632	0.0024048	2.6189	0.0046759
C3	7.0498	0.0027244	2.5647	0.0052682
TG(16:1_34:1)	6.5077	0.0037856	2.4219	0.0072801
lysoPC a C24:0	6.2139	0.0045588	2.3412	0.008719
Cer(d16:1/18:0)	6.1941	0.0046173	2.3356	0.0087828
PC aa C30:2	6.0733	0.0049931	2.3016	0.0094464
TG(22:5_34:1)	6.0394	0.0051047	2.292	0.0096056
FA(20:3)	5.5618	0.0070325	2.1529	0.013162
Hex2Cer(d18:1/20:0)	5.5205	0.0072358	2.1405	0.013471
TG(18:1_36:2)	5.2923	0.0084896	2.0711	0.015721
DG(18:2_20:4)	5.1873	0.0091497	2.0386	0.016855
Lys	5.179	0.0092043	2.036	0.016866
Cer(d18:2/20:0)	5.1627	0.0093126	2.0309	0.016976
TG(20:1_24:3)	4.9365	0.010982	1.9593	0.019916
TG(22:6_34:1)	4.8819	0.011436	1.9417	0.020633
DG(14:0_14:0)	4.5854	0.014312	1.8443	0.02564
ProBetaine	4.5812	0.014358	1.8429	0.02564
C3-DC (C4-OH)	4.4133	0.016361	1.7862	0.029068
lysoPC a C16:0	4.3627	0.017027	1.7689	0.030099
TG(20:4_36:2)	4.1426	0.020313	1.6922	0.035726
FA(20:2)	4.126	0.020589	1.6864	0.036031
Xanthine	4.0596	0.021738	1.6628	0.037852
Creatinine	3.9955	0.022918	1.6398	0.039709
DG(18:1_20:1)	3.9497	0.023805	1.6233	0.041043
Trigonelline	3.8383	0.026136	1.5828	0.044841
TG(18:2_33:1)	3.7982	0.027039	1.568	0.046163

Table S1

Metabolite	t.stat	p.value	p-value (-log10)	FDR
C2	67.534	2.88E-07	6.5406	9.10E-05
PC ae C42:1	56.961	5.69E-07	6.2451	9.10E-05
Pro	44.562	1.52E-06	5.8192	0.000128
Hypoxanthine	43.998	1.60E-06	5.7971	0.000128
Gly	37.194	3.12E-06	5.5058	0.00019
PC aa C30:0	35.428	3.79E-06	5.4216	0.00019
PC ae C38:2	34.595	4.17E-06	5.3803	0.00019
TG(20:4_36:2)	-31.278	6.23E-06	5.2058	0.000222
Choline	31.24	6.26E-06	5.2037	0.000222
Cer(d18:1/24:1)	-28.095	9.55E-06	5.02	0.000279
PC aa C36:3	28.054	9.60E-06	5.0175	0.000279
AconAcid	27.292	1.07E-05	4.9698	0.000286
Cys	26.533	1.20E-05	4.9211	0.000295
Cer(d16:1/18:0)	-25.794	1.34E-05	4.8723	0.0003
Glu	25.486	1.41E-05	4.8515	0.0003
Ala	24.071	1.77E-05	4.7528	0.000343
H1	-23.88	1.82E-05	4.7391	0.000343
Thr	23.003	2.12E-05	4.6744	0.000346
Tyr	22.894	2.16E-05	4.6662	0.000346
PC aa C40:1	22.418	2.34E-05	4.63	0.000346
t4-OH-Pro	22.313	2.39E-05	4.6219	0.000346
C16	22.232	2.42E-05	4.6156	0.000346
PC aa C38:5	22.087	2.49E-05	4.6043	0.000346
Lac	21.723	2.66E-05	4.5756	0.000354
PC aa C34:1	20.747	3.19E-05	4.4964	0.000402
Hex2Cer(d18:1/24:1)	-20.598	3.28E-05	4.484	0.000402
PC ae C42:0	20.307	3.47E-05	4.4594	0.000402
Gln	20.242	3.52E-05	4.4539	0.000402
PC aa C42:6	19.93	3.74E-05	4.4271	0.000404
PC aa C32:3	19.873	3.78E-05	4.4222	0.000404
CE(20:4)	-19.607	3.99E-05	4.399	0.000412
Betaine	18.781	4.73E-05	4.3249	0.000473
PC aa C36:2	18.432	5.10E-05	4.2926	0.000492
PC aa C34:2	18.315	5.23E-05	4.2816	0.000492
Asn	18.116	5.46E-05	4.2629	0.000499
PC aa C32:2	17.854	5.78E-05	4.2378	0.000514
PAG	17.599	6.12E-05	4.2131	0.000529
His	17.421	6.37E-05	4.1956	0.000534
Hex2Cer(d18:1/16:0)	-17.331	6.50E-05	4.1868	0.000534
HexCer(d18:1/16:0)	-16.945	7.11E-05	4.1481	0.000569
beta-Ala	16.521	7.86E-05	4.1045	0.0006

PC aa C32:1	16.475	7.95E-05	4.0998	0.0006
PC ae C34:2	-16.396	8.10E-05	4.0915	0.0006
lysoPC a C20:4	-16.285	8.32E-05	4.0798	0.0006
PC ae C42:3	16.225	8.44E-05	4.0735	0.0006
Met	15.999	8.92E-05	4.0495	0.000608
C0	15.995	8.93E-05	4.0491	0.000608
PC aa C36:5	15.453	0.00010235	3.9899	0.000657
PC aa C36:4	15.45	0.00010243	3.9896	0.000657
Phe	15.358	0.00010488	3.9793	0.000657
lysoPC a C18:1	-15.291	0.00010669	3.9719	0.000657
Spermine	15.286	0.00010682	3.9713	0.000657
PC ae C42:2	15.126	0.00011137	3.9532	0.000672
PC aa C38:0	-15.051	0.00011356	3.9448	0.000673
Leu	14.983	0.00011559	3.9371	0.000673
PC aa C38:4	14.838	0.00012011	3.9204	0.000686
PC aa C38:6	14.631	0.00012696	3.8963	0.000713
TMAO	14.515	0.00013101	3.8827	0.000723
PC aa C34:3	14.188	0.0001433	3.8437	0.000777
PC ae C38:3	13.978	0.00015193	3.8184	0.000805
lysoPC a C28:0	13.945	0.00015337	3.8142	0.000805
PC aa C36:0	-13.739	0.00016263	3.7888	0.000816
PC ae C38:0	13.729	0.00016308	3.7876	0.000816
C3-DC (C4-OH)	13.728	0.00016311	3.7875	0.000816
Cer(d18:1/14:0)	-13.552	0.00017159	3.7655	0.000845
PC ae C30:0	13.435	0.00017753	3.7507	0.000861
PC ae C44:4	13.152	0.00019301	3.7144	0.000922
Cer(d18:2/18:0)	-13.066	0.00019807	3.7032	0.000932
Cer(d18:2/16:0)	-12.953	0.00020491	3.6884	0.00095
Hex2Cer(d18:1/14:0)	-12.517	0.00023436	3.6301	0.001071
AA	-12.472	0.00023767	3.624	0.001071
Cer(d18:2/24:1)	-11.826	0.00029269	3.5336	0.001301
PC aa C38:3	11.706	0.00030453	3.5164	0.001319
lysoPC a C18:2	-11.701	0.00030512	3.5155	0.001319
HexCer(d18:1/24:1)	-11.619	0.0003136	3.5036	0.001336
Trp	11.557	0.00032016	3.4946	0.001336
Arg	-11.545	0.00032147	3.4929	0.001336
HexCer(d18:2/16:0)	-11.385	0.00033944	3.4692	0.001393
Asp	11.262	0.00035413	3.4508	0.001435
Val	11.174	0.00036515	3.4375	0.001461
Ile	10.814	0.00041485	3.3821	0.001621
SM C18:0	-10.81	0.00041537	3.3816	0.001621
lysoPC a C16:1	-10.736	0.00042664	3.3699	0.001628
Cer(d18:1/16:0)	-10.732	0.00042734	3.3692	0.001628

PC ae C40:1	10.51	0.00046345	3.334	0.001745
PC ae C32:2	-10.399	0.00048298	3.3161	0.001797
PC ae C34:3	-10.36	0.00049001	3.3098	0.001802
AABA	10.047	0.0005519	3.2581	0.002007
PC aa C36:6	9.9249	0.00057865	3.2376	0.002081
PC ae C36:5	-9.8293	0.00060072	3.2213	0.002116
PC aa C40:6	9.8251	0.00060171	3.2206	0.002116
PC aa C42:5	9.7373	0.00062295	3.2055	0.002149
PC ae C34:1	9.7281	0.00062524	3.204	0.002149
lysoPC a C18:0	-9.7035	0.00063139	3.1997	0.002149
SM (OH) C22:2	-9.6	0.00065808	3.1817	0.002217
PC ae C44:3	9.5701	0.00066606	3.1765	0.00222
lysoPC a C20:3	-9.4101	0.00071082	3.1482	0.002345
Suc	9.3773	0.00072045	3.1424	0.002353
PC aa C32:0	9.3231	0.00073674	3.1327	0.002381
HexCer(d18:1/20:0)	-9.0296	0.00083323	3.0792	0.002666
PC aa C34:4	8.9185	0.00087385	3.0586	0.002769
PC ae C36:1	8.8911	0.00088421	3.0534	0.002774
Cer(d18:1/18:0)	-8.8138	0.00091436	3.0389	0.002841
DG(16:0_18:1)	-8.7061	0.00095848	3.0184	0.002949
Trigonelline	8.6096	0.0010003	2.9999	0.003049
Serotonin	8.4318	0.0010834	2.9652	0.003271
TCDCA	-8.3843	0.0011071	2.9558	0.003311
HCys	8.2578	0.0011732	2.9306	0.003476
Putrescine	-8.1391	0.0012398	2.9066	0.00364
Hex2Cer(d18:1/18:0)	-8.1097	0.001257	2.9007	0.003657
HexCer(d18:1/14:0)	-7.7546	0.0014901	2.8268	0.004296
PC aa C40:5	7.6397	0.0015769	2.8022	0.004505
TG(22:6_32:1)	-7.5432	0.0016546	2.7813	0.004686
SM C24:1	-7.3685	0.0018077	2.7429	0.005074
Hex2Cer(d18:1/24:0)	-7.3235	0.0018498	2.7329	0.005147
PC ae C38:6	-7.0203	0.0021685	2.6638	0.005982
PC aa C42:2	-6.9543	0.0022466	2.6485	0.00611
Cystine	-6.9489	0.0022531	2.6472	0.00611
TLCA	-6.8184	0.0024187	2.6164	0.006504
PC ae C42:5	-6.6782	0.0026136	2.5828	0.00697
Ser	6.6454	0.0026619	2.5748	0.00704
3-Met-His	6.6142	0.0027089	2.5672	0.007105
HexCer(d18:1/18:0)	-6.5696	0.0027779	2.5563	0.007227
Cer(d18:0/24:1)	-6.4184	0.0030286	2.5188	0.007816
DG(18:1_18:1)	-6.3492	0.0031526	2.5013	0.008071
Spermidine	-6.2396	0.0033619	2.4734	0.008538
5-AVA	6.2171	0.0034069	2.4676	0.008584

PC ae C36:2	6.112	0.0036276	2.4404	0.009011
1-Met-His	6.1098	0.0036326	2.4398	0.009011
HexCer(d18:1/22:0)	-5.9976	0.0038883	2.4102	0.009571
PC ae C36:0	5.8194	0.0043416	2.3624	0.010581
PC ae C40:6	-5.8109	0.0043647	2.36	0.010581
PC aa C42:4	5.6625	0.0047953	2.3192	0.011538
Cer(d18:1/24:0)	-5.6179	0.0049346	2.3067	0.011784
HArg	5.5608	0.0051207	2.2907	0.012138
PC ae C36:4	5.547	0.005167	2.2868	0.012158
GABA	5.4918	0.0053569	2.2711	0.012512
PC aa C40:4	5.3586	0.0058519	2.2327	0.01357
Dopamine	-5.2663	0.0062277	2.2057	0.014337
SM C16:0	5.1889	0.0065661	2.1827	0.015008
TG(22:5_34:1)	-5.1136	0.0069168	2.1601	0.015698
C4	5.0048	0.0074648	2.127	0.016822
Cer(d18:2/14:0)	-4.9809	0.0075923	2.1196	0.01699
PC aa C28:1	4.9004	0.0080416	2.0947	0.01787
lysoPC a C26:0	4.7343	0.0090752	2.0421	0.019944
Lys	4.7307	0.0090997	2.041	0.019944
PC ae C44:6	-4.6914	0.009368	2.0284	0.020393
Cer(d18:2/22:0)	-4.6207	0.0098763	2.0054	0.021326
PC ae C32:1	-4.6134	0.0099301	2.003	0.021326
lysoPC a C17:0	-4.5891	0.010114	1.9951	0.021576
Cer(d18:2/20:0)	-4.555	0.010378	1.9839	0.021993
PC aa C40:3	4.4595	0.011165	1.9521	0.023505
TrpBetaine	4.4444	0.011296	1.9471	0.023625
ProBetaine	4.3746	0.011925	1.9235	0.02478
Cer(d16:1/22:0)	-4.2355	0.013312	1.8758	0.027482
Orn	4.1957	0.013743	1.8619	0.02819
PC ae C44:5	4.0526	0.015442	1.8113	0.031475
TDCA	-4.0011	0.016116	1.7927	0.03264
PC aa C24:0	3.9776	0.016434	1.7843	0.033075
SM C18:1	-3.9691	0.016551	1.7812	0.033101
Cer(d18:1/22:0)	-3.9541	0.016761	1.7757	0.033313
HexCer(d18:1/24:0)	-3.8443	0.018392	1.7354	0.03633
FA(20:3)	-3.8241	0.018712	1.7279	0.036736
OH-GlutAcid	3.7831	0.019383	1.7126	0.037821
SM C16:1	3.7291	0.020314	1.6922	0.0391
Cit	3.7192	0.020489	1.6885	0.0391
PC aa C38:1	-3.7188	0.020497	1.6883	0.0391
PC ae C40:3	3.717	0.020527	1.6877	0.0391
GDCA	-3.6955	0.020917	1.6795	0.039606
TG(20:4_34:1)	-3.6732	0.021331	1.671	0.040152

FA(18:1)	-3.6218	0.022322	1.6513	0.041773
DiCA(12:0)	-3.5892	0.022977	1.6387	0.042749
Cer(d18:2/24:0)	-3.5067	0.024747	1.6065	0.045774
PC ae C38:5	-3.4416	0.026256	1.5808	0.048286
Cer(d18:1/20:0)	-3.3808	0.027764	1.5565	0.050768
HexCer(d18:2/24:0)	-3.1678	0.03393	1.4694	0.06169
DG(16:0_20:3)	-3.1172	0.035622	1.4483	0.064401
HexCer(d18:2/18:0)	-2.9556	0.041737	1.3795	0.075033
lysoPC a C26:1	2.9129	0.043552	1.361	0.077858
HexCer(d18:1/26:1)	-2.8833	0.044866	1.3481	0.079762
SM (OH) C16:1	-2.8496	0.046417	1.3333	0.082063
Met-SO	2.8256	0.047555	1.3228	0.083485
Cer(d18:1/23:0)	-2.8203	0.047814	1.3204	0.083485
Hex2Cer(d18:1/22:0)	-2.8164	0.048004	1.3187	0.083485

Table S2

Metabolite	FC	log2(FC)	raw.pval	p-value (-log10)
C2	20.387	4.3496	2.88E-07	6.5406
PC ae C42:1	2.6249	1.3922	5.69E-07	6.2451
Pro	15.535	3.9575	1.52E-06	5.8192
Hypoxanthine	11.149	3.4789	1.60E-06	5.7971
Gly	14.256	3.8335	3.12E-06	5.5058
PC aa C30:0	4.945	2.306	3.79E-06	5.4216
TG(20:4_36:2)	0.11916	-3.069	6.23E-06	5.2058
Choline	8.8375	3.1436	6.26E-06	5.2037
Cer(d18:1/24:1)	0.19221	-2.3793	9.55E-06	5.02
PC aa C36:3	2.8841	1.5281	9.60E-06	5.0175
AconAcid	5.0489	2.336	1.07E-05	4.9698
Cys	9.477	3.2444	1.20E-05	4.9211
Cer(d16:1/18:0)	0.1147	-3.124	1.34E-05	4.8723
Glu	7.3189	2.8716	1.41E-05	4.8515
Ala	4.7176	2.2381	1.77E-05	4.7528
H1	0.30823	-1.6979	1.82E-05	4.7391
Thr	4.0059	2.0021	2.12E-05	4.6744
Tyr	3.0518	1.6097	2.16E-05	4.6662
PC aa C40:1	3.6638	1.8734	2.34E-05	4.63
t4-OH-Pro	4.1125	2.04	2.39E-05	4.6219
C16	3.6978	1.8867	2.42E-05	4.6156
PC aa C38:5	2.1827	1.1261	2.49E-05	4.6043
Lac	5.2924	2.4039	2.66E-05	4.5756
PC aa C34:1	2.2434	1.1657	3.19E-05	4.4964
Hex2Cer(d18:1/24:1)	0.13084	-2.9341	3.28E-05	4.484
PC ae C42:0	2.1748	1.1209	3.47E-05	4.4594
Gln	3.2741	1.7111	3.52E-05	4.4539
PC aa C32:3	2.7242	1.4458	3.78E-05	4.4222
CE(20:4)	0.10637	-3.2328	3.99E-05	4.399
Betaine	3.608	1.8512	4.73E-05	4.3249
PC aa C36:2	2.2552	1.1732	5.10E-05	4.2926
PC aa C34:2	2.4878	1.3149	5.23E-05	4.2816
Asn	4.4873	2.1659	5.46E-05	4.2629
PC aa C32:2	2.4174	1.2735	5.78E-05	4.2378
PAG	3.6618	1.8726	6.12E-05	4.2131
His	3.0219	1.5954	6.37E-05	4.1956
Hex2Cer(d18:1/16:0)	0.27575	-1.8586	6.50E-05	4.1868
HexCer(d18:1/16:0)	0.28118	-1.8304	7.11E-05	4.1481
beta-Ala	43.48	5.4423	7.86E-05	4.1045
PC aa C32:1	2.598	1.3774	7.95E-05	4.0998
lysoPC a C20:4	0.33023	-1.5984	8.32E-05	4.0798

Met	2.5301	1.3392	8.92E-05	4.0495
C0	3.6741	1.8774	8.93E-05	4.0491
PC aa C36:5	2.0759	1.0537	0.00010235	3.9899
PC aa C36:4	2.1822	1.1258	0.00010243	3.9896
Phe	2.9446	1.5581	0.00010488	3.9793
lysoPC a C18:1	0.43725	-1.1935	0.00010669	3.9719
Spermine	2.9957	1.5829	0.00010682	3.9713
PC ae C42:2	2.0738	1.0523	0.00011137	3.9532
Leu	2.5322	1.3404	0.00011559	3.9371
PC aa C38:6	2.3051	1.2048	0.00012696	3.8963
TMAO	2.4065	1.2669	0.00013101	3.8827
PC aa C34:3	2.5559	1.3538	0.0001433	3.8437
lysoPC a C28:0	2.1708	1.1182	0.00015337	3.8142
C3-DC (C4-OH)	3.8968	1.9623	0.00016311	3.7875
Cer(d18:1/14:0)	0.27432	-1.8661	0.00017159	3.7655
PC ae C30:0	2.2257	1.1542	0.00017753	3.7507
Cer(d18:2/18:0)	0.14838	-2.7526	0.00019807	3.7032
Cer(d18:2/16:0)	0.37499	-1.4151	0.00020491	3.6884
Hex2Cer(d18:1/14:0)	0.24931	-2.004	0.00023436	3.6301
AA	0.47456	-1.0753	0.00023767	3.624
Cer(d18:2/24:1)	0.22329	-2.163	0.00029269	3.5336
HexCer(d18:1/24:1)	0.16108	-2.6341	0.0003136	3.5036
Trp	5.1112	2.3537	0.00032016	3.4946
Arg	0.027078	-5.2067	0.00032147	3.4929
HexCer(d18:2/16:0)	0.27563	-1.8592	0.00033944	3.4692
Asp	7.3053	2.8689	0.00035413	3.4508
Val	3.0461	1.607	0.00036515	3.4375
Ile	2.6817	1.4232	0.00041485	3.3821
SM C18:0	0.49312	-1.02	0.00041537	3.3816
Cer(d18:1/16:0)	0.19462	-2.3613	0.00042734	3.3692
AABA	3.3106	1.7271	0.0005519	3.2581
Suc	2.4909	1.3167	0.00072045	3.1424
HexCer(d18:1/20:0)	0.17713	-2.4971	0.00083323	3.0792
Cer(d18:1/18:0)	0.11898	-3.0712	0.00091436	3.0389
Trigonelline	3.0481	1.6079	0.0010003	2.9999
Serotonin	2.235	1.1603	0.0010834	2.9652
HCys	2.067	1.0475	0.0011732	2.9306
Putrescine	0.49618	-1.0111	0.0012398	2.9066
Hex2Cer(d18:1/18:0)	0.21235	-2.2355	0.001257	2.9007
HexCer(d18:1/14:0)	0.36964	-1.4358	0.0014901	2.8268
TG(22:6_32:1)	0.070157	-3.8333	0.0016546	2.7813
Hex2Cer(d18:1/24:0)	0.070692	-3.8223	0.0018498	2.7329
Cystine	0.30507	-1.7128	0.0022531	2.6472

TLCA	0.49027	-1.0283	0.0024187	2.6164
3-Met-His	3.279	1.7133	0.0027089	2.5672
HexCer(d18:1/18:0)	0.1003	-3.3177	0.0027779	2.5563
Cer(d18:0/24:1)	0.44036	-1.1832	0.0030286	2.5188
5-AVA	28.249	4.8201	0.0034069	2.4676
1-Met-His	2.6536	1.4079	0.0036326	2.4398
HexCer(d18:1/22:0)	0.15768	-2.6649	0.0038883	2.4102
Cer(d18:1/24:0)	0.35647	-1.4881	0.0049346	2.3067
GABA	7.0139	2.8102	0.0053569	2.2711
TG(22:5_34:1)	0.2649	-1.9165	0.0069168	2.1601
C4	10.551	3.3994	0.0074648	2.127
Cer(d18:2/14:0)	0.35875	-1.4789	0.0075923	2.1196
Cer(d18:2/22:0)	0.0953	-3.3914	0.0098763	2.0054
Cer(d18:2/20:0)	0.090911	-3.4594	0.010378	1.9839
TrpBetaine	9.1152	3.1883	0.011296	1.9471
Cer(d16:1/22:0)	0.12253	-3.0288	0.013312	1.8758
Cer(d18:1/22:0)	0.11178	-3.1612	0.016761	1.7757
HexCer(d18:1/24:0)	0.20307	-2.2999	0.018392	1.7354
OH-GlutAcid	5.3878	2.4297	0.019383	1.7126
PC aa C38:1	0.30477	-1.7142	0.020497	1.6883
TG(20:4_34:1)	0.13517	-2.8872	0.021331	1.671
Cer(d18:2/24:0)	0.25644	-1.9633	0.024747	1.6065
Cer(d18:1/20:0)	0.16523	-2.5974	0.027764	1.5565
HexCer(d18:2/24:0)	0.41812	-1.258	0.03393	1.4694
HexCer(d18:2/18:0)	0.21698	-2.2043	0.041737	1.3795
HexCer(d18:1/26:1)	0.21137	-2.2422	0.044866	1.3481
Met-SO	4.1568	2.0555	0.047555	1.3228
Cer(d18:1/23:0)	0.16412	-2.6072	0.047814	1.3204
Hex2Cer(d18:1/22:0)	0.26667	-1.9069	0.048004	1.3187

Table S3

Metabolite	FC	log2(FC)	raw.pval	p-value (-log10)
TG(22:2_32:4)	0.15405	-2.6985	8.51E-05	4.0698
Cer(d18:1/16:0)	3.6562	1.8703	0.0001709	3.7673
C2	0.080956	-3.6267	0.00020062	3.6976
Taurine	0.043183	-4.5334	0.00022994	3.6384
Val	0.36687	-1.4466	0.00023315	3.6324
PC aa C34:2	0.42967	-1.2187	0.00031907	3.4961
Hex2Cer(d18:1/16:0)	2.9	1.536	0.00032352	3.4901
C16	0.18845	-2.4078	0.00035647	3.448
lysoPC a C20:4	3.1239	1.6433	0.00036879	3.4332
C0	0.19549	-2.3548	0.00038284	3.417
Gly	0.099437	-3.3301	0.00043325	3.3633
Pro	0.084449	-3.5658	0.00044432	3.3523
lysoPC a C16:1	2.0519	1.0369	0.00051671	3.2868
Trp	0.21183	-2.239	0.00052878	3.2767
HexCer(d18:1/18:0)	5.8867	2.5575	0.0007411	3.1301
HexCer(d18:1/20:0)	3.7294	1.899	0.0008211	3.0856
lysoPC a C18:1	2.845	1.5084	0.00093075	3.0312
beta-Ala	0.042703	-4.5495	0.00095875	3.0183
Choline	0.14657	-2.7703	0.0010147	2.9936
Thr	0.32442	-1.6241	0.0010843	2.9648
PC aa C32:2	0.42019	-1.2509	0.0011396	2.9433
Betaine	0.19698	-2.3439	0.0011786	2.9286
HexCer(d18:2/16:0)	4.0185	2.0066	0.0012819	2.8921
TG(18:3_32:1)	6.2842	2.6517	0.0013243	2.878
Phe	0.40501	-1.304	0.0013357	2.8743
PC ae C30:0	0.46429	-1.1069	0.0013661	2.8645
Tyr	0.36402	-1.4579	0.0018628	2.7298
PC aa C30:0	0.24106	-2.0525	0.0020078	2.6973
Hex2Cer(d18:1/24:1)	3.1737	1.6661	0.0020126	2.6962
Cystine	5.516	2.4636	0.0020525	2.6877
TG(17:1_32:1)	8.1307	3.0234	0.0020734	2.6833
Hypoxanthine	0.18219	-2.4564	0.0022046	2.6567
H1	2.2799	1.1889	0.0022418	2.6494
Cer(d18:1/14:0)	3.0189	1.594	0.0024377	2.613
AconAcid	0.33041	-1.5977	0.0024382	2.6129
Hex2Cer(d18:1/18:0)	3.5053	1.8095	0.0026105	2.5833
HexCer(d18:1/16:0)	2.9595	1.5654	0.0028611	2.5435
Cer(d18:2/18:0)	3.1837	1.6707	0.0029762	2.5263
PC aa C32:3	0.30103	-1.732	0.003057	2.5147
HexCer(d18:1/22:0)	2.6984	1.4321	0.0030771	2.5119
TG(22:5_34:3)	0.11766	-3.0873	0.0034576	2.4612

lysoPC a C26:0	0.45365	-1.1404	0.0037186	2.4296
PC aa C36:2	0.47101	-1.0862	0.0037937	2.4209
Hex2Cer(d18:1/24:0)	10.867	3.4419	0.0037962	2.4207
Cer(d18:1/24:1)	2.4391	1.2864	0.0041564	2.3813
PC aa C32:1	0.4102	-1.2856	0.0044239	2.3542
Glu	0.25061	-1.9965	0.0044569	2.351
Lac	0.32077	-1.6404	0.0048023	2.3185
His	0.39157	-1.3526	0.0048084	2.318
Cys	0.23465	-2.0914	0.005139	2.2891
PAG	0.1706	-2.5513	0.0052999	2.2757
lysoPC a C20:3	2.3754	1.2482	0.0053831	2.269
3-Met-His	0.32868	-1.6052	0.0055805	2.2533
HCys	0.48041	-1.0577	0.0060706	2.2168
PC aa C34:3	0.41815	-1.2579	0.0064643	2.1895
5-AVA	0.047435	-4.3979	0.006637	2.178
PC aa C36:3	0.41131	-1.2817	0.0069719	2.1566
ProBetaine	0.45958	-1.1216	0.0070209	2.1536
Cer(d18:1/18:0)	5.0694	2.3418	0.0076988	2.1136
PC aa C32:0	0.4782	-1.0643	0.0088888	2.0512
Asn	0.35963	-1.4754	0.008982	2.0466
Ala	0.3009	-1.7327	0.0097374	2.0116
HexCer(d18:1/24:1)	2.355	1.2357	0.010006	1.9997
FA(20:3)	2.3407	1.2269	0.010333	1.9858
C4	0.13001	-2.9433	0.010522	1.9779
GABA	0.23932	-2.063	0.010636	1.9732
Cer(d18:2/16:0)	2.5578	1.3549	0.010823	1.9657
Asp	0.18332	-2.4475	0.011201	1.9507
Cer(d18:1/22:0)	3.2182	1.6862	0.011289	1.9473
Ile	0.44989	-1.1524	0.011453	1.9411
PC aa C34:1	0.42422	-1.2371	0.01205	1.919
AA	2.4514	1.2936	0.01285	1.8911
Hex2Cer(d18:1/14:0)	2.6318	1.396	0.013003	1.8859
Cer(d18:2/14:0)	3.4551	1.7887	0.013239	1.8782
Cer(d18:1/20:0)	2.3467	1.2306	0.013523	1.8689
t4-OH-Pro	0.36251	-1.4639	0.013668	1.8643
Leu	0.46472	-1.1056	0.01521	1.8179
Met	0.46268	-1.1119	0.016529	1.7818
HexCer(d18:1/14:0)	2.7985	1.4846	0.018937	1.7227
AABA	0.46938	-1.0912	0.022863	1.6409
Cer(d18:2/24:1)	2.3436	1.2287	0.027303	1.5638
Cer(d18:2/24:0)	2.5012	1.3226	0.027707	1.5574
Trigonelline	0.34935	-1.5172	0.033658	1.4729
Met-SO	0.22047	-2.1813	0.041691	1.38

C3	0.29028	-1.7845	0.042385	1.3728
OH-GlutAcid	0.24849	-2.0087	0.042552	1.3711
TG(20:4_36:2)	2.9212	1.5466	0.044153	1.355
Gln	0.44859	-1.1565	0.045739	1.3397
Spermine	0.40467	-1.3052	0.045893	1.3383
Cer(d18:1/23:0)	3.4903	1.8033	0.046216	1.3352
Cer(d16:1/22:0)	4.7018	2.2332	0.047299	1.3251
1-Met-His	0.48694	-1.0382	0.049001	1.3098

Table S4

Metabolite	FC	log2(FC)	raw.pval	p-value (-log10)
Cys	0.053954	-4.2121	4.63E-08	7.3345
C2	0.028632	-5.1262	5.48E-08	7.2613
Glu	0.065433	-3.9338	5.79E-08	7.2377
Taurine	0.01398	-6.1605	1.19E-07	6.925
Pro	0.034287	-4.8662	1.49E-07	6.827
Ala	0.084797	-3.5598	1.95E-07	6.7102
Spermine	0.033408	-4.9037	3.87E-07	6.4118
C0	0.086294	-3.5346	3.88E-07	6.4112
C16	0.080071	-3.6426	4.49E-07	6.3478
Betaine	0.084674	-3.5619	5.84E-07	6.2333
AconAcid	0.054223	-4.2049	7.47E-07	6.1264
Gly	0.040628	-4.6214	7.54E-07	6.1226
Lac	0.068295	-3.8721	1.13E-06	5.9481
Ile	0.1313	-2.9291	1.16E-06	5.9373
HCys	0.18572	-2.4288	1.32E-06	5.8789
PAG	0.077848	-3.6832	1.84E-06	5.734
Thr	0.1077	-3.2149	2.22E-06	5.6539
TG(20:1_31:0)	0.08164	-3.6146	2.48E-06	5.6064
Ser	0.2421	-2.0463	2.82E-06	5.549
Choline	0.081832	-3.6112	3.03E-06	5.518
Hypoxanthine	0.083862	-3.5758	3.30E-06	5.4818
His	0.11863	-3.0755	3.43E-06	5.4643
Tyr	0.12516	-2.9982	3.44E-06	5.4631
PC ae C40:6	9.5432	3.2545	3.73E-06	5.4279
t4-OH-Pro	0.093098	-3.4251	4.44E-06	5.353
Met	0.15698	-2.6714	4.58E-06	5.3396
Cer(d18:2/16:0)	12.179	3.6063	4.99E-06	5.3023
PC ae C44:6	2.3534	1.2348	5.11E-06	5.2915
Lys	0.16894	-2.5654	5.66E-06	5.2471
Arg	0.037721	-4.7285	6.00E-06	5.2218
Leu	0.14085	-2.8278	7.07E-06	5.1503
PC ae C38:6	8.7431	3.1282	8.05E-06	5.0944
PC aa C40:4	12.551	3.6498	8.28E-06	5.0822
Gln	0.10146	-3.301	8.60E-06	5.0656
PC aa C42:2	2.3674	1.2433	8.61E-06	5.065
PC ae C38:4	9.0454	3.1772	9.03E-06	5.0441
Spermidine	0.22006	-2.184	9.49E-06	5.0226
Val	0.12769	-2.9692	9.94E-06	5.0028
Orn	0.16022	-2.6419	1.01E-05	4.9942
TG(18:2_32:2)	0.080722	-3.6309	1.01E-05	4.9941
Cit	0.27429	-1.8662	1.05E-05	4.977

DG(16:0_18:1)	2.435	1.2839	1.07E-05	4.9691
Asn	0.087755	-3.5104	1.19E-05	4.9239
Phe	0.13417	-2.8978	1.20E-05	4.9224
PC ae C42:5	2.4401	1.2869	1.23E-05	4.9087
TG(17:2_34:3)	0.07363	-3.7636	1.41E-05	4.8504
PC ae C42:0	2.4798	1.3102	1.68E-05	4.7758
alpha-AAA	0.073069	-3.7746	1.76E-05	4.7543
HexCer(d18:1/24:1)	2.3952	1.2601	1.83E-05	4.7375
PC aa C36:0	2.4929	1.3178	2.01E-05	4.6978
H1	0.30539	-1.7113	2.28E-05	4.6412
PC ae C38:5	7.4227	2.8919	2.55E-05	4.5928
PC aa C40:6	4.4517	2.1543	2.74E-05	4.5616
PC aa C42:0	2.3759	1.2485	2.96E-05	4.5284
Carnosine	0.18476	-2.4363	3.09E-05	4.5096
PC aa C40:5	7.6669	2.9387	3.22E-05	4.4918
beta-Ala	0.012343	-6.3402	3.61E-05	4.443
DG(18:1_18:1)	2.4833	1.3123	3.72E-05	4.4291
Suc	0.13833	-2.8538	3.81E-05	4.4186
PC ae C40:5	9.9839	3.3196	4.28E-05	4.3683
Serotonin	0.16076	-2.637	4.35E-05	4.3612
PC ae C36:4	5.5421	2.4704	5.22E-05	4.2823
PC aa C38:3	6.8459	2.7752	5.56E-05	4.2547
HArg	0.16393	-2.6089	5.68E-05	4.2453
PC ae C40:4	7.199	2.8478	5.71E-05	4.2434
PC aa C38:0	8.8384	3.1438	5.91E-05	4.2286
HexCer(d18:1/16:0)	27.85	4.7996	6.02E-05	4.2204
ProBetaine	0.22041	-2.1817	6.09E-05	4.2156
PC aa C38:4	6.2381	2.6411	6.76E-05	4.1701
Trp	0.082038	-3.6076	6.99E-05	4.1557
TMAO	0.14332	-2.8027	7.14E-05	4.1464
Asp	0.060318	-4.0513	7.95E-05	4.0996
Hex2Cer(d18:1/16:0)	17.921	4.1635	9.84E-05	4.0069
SM C18:0	37.622	5.2335	0.00010671	3.9718
PC aa C42:5	9.5258	3.2518	0.00010803	3.9664
PC ae C44:5	2.7256	1.4466	0.00011013	3.9581
TG(20:4_36:2)	2.6461	1.4039	0.00012728	3.8952
Cer(d18:1/16:0)	35.751	5.1599	0.0001366	3.8646
Trigonelline	0.19208	-2.3802	0.00014076	3.8515
SM (OH) C22:2	2.4764	1.3083	0.00014171	3.8486
Putrescine	0.45763	-1.1278	0.00014508	3.8384
AABA	0.11804	-3.0827	0.00014999	3.8239
TG(17:0_34:2)	0.058278	-4.1009	0.00015177	3.8188
lysoPC a C20:4	2.7846	1.4775	0.00017879	3.7477

PC ae C36:5	10.676	3.4163	0.00019191	3.7169
DG(16:0_20:3)	2.3666	1.2428	0.00019478	3.7105
PC ae C40:3	3.8908	1.9601	0.00020454	3.6892
PC aa C40:2	10.181	3.3478	0.0002122	3.6733
PC aa C36:1	19.221	4.2646	0.0002396	3.6205
PC ae C40:1	2.0644	1.0457	0.00024349	3.6135
Xanthine	0.19337	-2.3706	0.00027227	3.565
PC aa C40:3	5.1683	2.3697	0.00028102	3.5513
Cer(d16:1/18:0)	2.7497	1.4593	0.0002837	3.5471
PC aa C38:6	2.5154	1.3308	0.00029084	3.5363
PC ae C40:2	3.7645	1.9125	0.00029358	3.5323
1-Met-His	0.13876	-2.8494	0.00031253	3.5051
Dopamine	0.40902	-1.2898	0.00032818	3.4839
PC aa C38:5	2.6843	1.4245	0.00033121	3.4799
TG(18:2_33:1)	0.064617	-3.9519	0.00034058	3.4678
DG(16:0_18:2)	2.7501	1.4595	0.00037033	3.4314
3-Met-His	0.10836	-3.2061	0.00041353	3.3835
PC ae C32:2	4.4475	2.153	0.00042144	3.3753
FA(20:1)	0.42619	-1.2304	0.00046561	3.332
PC ae C38:1	6.7371	2.7521	0.00048201	3.3169
SM (OH) C16:1	5.7627	2.5267	0.00048373	3.3154
Cer(d18:0/24:1)	2.8423	1.5071	0.00050431	3.2973
PC ae C42:4	2.8896	1.5309	0.00053477	3.2718
PC ae C34:3	3.9566	1.9843	0.00056869	3.2451
PC ae C44:3	2.2092	1.1435	0.00064394	3.1912
PC ae C36:1	6.9559	2.7982	0.00074004	3.1307
PC aa C36:4	2.5165	1.3314	0.00074711	3.1266
PC aa C34:1	3.7876	1.9213	0.00079284	3.1008
Cystine	0.45191	-1.1459	0.00080521	3.0941
SM C24:1	24.885	4.6372	0.00080886	3.0921
SM C18:1	5.0208	2.3279	0.00097986	3.0088
PC ae C38:3	3.7412	1.9035	0.00098203	3.0079
Cer(d18:2/18:0)	2.633	1.3967	0.00099032	3.0042
AA	2.0724	1.0513	0.0010169	2.9927
PC aa C32:0	6.315	2.6588	0.0010275	2.9882
PC ae C36:3	5.3264	2.4132	0.0010452	2.9808
PC ae C34:0	8.0027	3.0005	0.0011742	2.9302
FA(18:2)	0.4544	-1.138	0.0011817	2.9275
Cer(d18:1/22:0)	2.6814	1.423	0.0013938	2.8558
HexCer(d18:1/18:1)	2.7562	1.4627	0.0014392	2.8419
PC ae C34:1	4.4175	2.1432	0.0015846	2.8001
PC ae C34:2	6.6383	2.7308	0.0015949	2.7973
Cer(d18:2/24:1)	48.978	5.6141	0.001635	2.7865

SM (OH) C14:1	2.7129	1.4398	0.001674	2.7762
SM C16:0	4.0747	2.0267	0.0017001	2.7695
Cer(d18:1/24:1)	60.011	5.9071	0.0017167	2.7653
SM C24:0	7.4749	2.9021	0.0017172	2.7652
TG(20:4_34:1)	2.6514	1.4067	0.0017456	2.758
PC ae C32:1	5.0347	2.3319	0.0020693	2.6842
DG(16:0_16:1)	2.5862	1.3708	0.0021028	2.6772
BABA	0.024552	-5.348	0.0024074	2.6184
PC ae C38:2	3.527	1.8184	0.0024365	2.6132
Cer(d18:2/20:0)	2.9811	1.5758	0.0024635	2.6084
Hex2Cer(d18:1/26:1)	3.4591	1.7904	0.002543	2.5946
C4	0.040648	-4.6207	0.0026826	2.5714
PC aa C36:3	2.0508	1.0362	0.002869	2.5423
PC aa C24:0	0.47753	-1.0663	0.0029017	2.5373
Cer(d18:1/24:0)	2.9162	1.5441	0.0029616	2.5285
PC ae C36:2	3.8293	1.9371	0.0031211	2.5057
Hex2Cer(d18:1/20:0)	3.1466	1.6538	0.0033476	2.4753
HexCer(d18:2/16:0)	23.758	4.5703	0.0034358	2.464
PC ae C30:1	4.1423	2.0504	0.0034749	2.4591
SDMA	0.10284	-3.2815	0.0035821	2.4459
GABA	0.092625	-3.4325	0.0035918	2.4447
HexCer(d18:2/23:0)	3.7045	1.8893	0.0037389	2.4273
Cer(d18:1/18:0)	18.344	4.1972	0.0038455	2.415
OH-GlutAcid	0.058411	-4.0976	0.0039371	2.4048
TG(20:1_24:3)	0.05685	-4.1367	0.003954	2.403
PC aa C38:1	23.711	4.5675	0.0040854	2.3888
DG(18:0_20:4)	3.41	1.7698	0.0042943	2.3671
DiCA(12:0)	0.3796	-1.3974	0.0046311	2.3343
Cer(d18:1/14:0)	14.636	3.8715	0.0046461	2.3329
Hex2Cer(d18:1/18:0)	2.5732	1.3636	0.0047111	2.3269
5-AVA	0.028391	-5.1384	0.0048155	2.3174
TG(14:0_34:1)	0.33901	-1.5606	0.0050877	2.2935
Hex2Cer(d18:1/24:1)	38.551	5.2687	0.0056863	2.2452
TG(17:1_32:1)	3.3008	1.7228	0.0060784	2.2162
PC ae C30:0	2.1735	1.12	0.0064619	2.1896
C3	0.090521	-3.4656	0.007093	2.1492
HexCer(d18:2/24:0)	3.7103	1.8915	0.0073163	2.1357
Hex2Cer(d18:1/22:0)	3.3851	1.7592	0.0081198	2.0905
PC aa C42:4	9.3889	3.231	0.0084112	2.0751
HexCer(d18:1/18:0)	16.42	4.0374	0.0098425	2.0069
TG(22:6_34:1)	3.3698	1.7526	0.0099192	2.0035
DG(14:0_14:0)	0.076783	-3.7031	0.010625	1.9737
HexCer(d18:1/22:0)	12.142	3.6019	0.011079	1.9555

Met-SO	0.089406	-3.4835	0.011265	1.9483
HexCer(d18:1/24:0)	9.1904	3.2001	0.011596	1.9357
PC aa C42:6	6.6814	2.7402	0.013536	1.8685
PC aa C42:1	2.0611	1.0434	0.014135	1.8497
HexCer(d18:1/23:0)	5.4165	2.4373	0.014363	1.8428
Hex2Cer(d18:1/24:0)	4.447	2.1528	0.015149	1.8196
Hex2Cer(d18:1/14:0)	6.4737	2.6946	0.015218	1.8176
HexCer(d18:1/20:0)	7.1845	2.8449	0.015586	1.8073
FA(14:0)	0.17396	-2.5232	0.017791	1.7498
TG(20:1_26:1)	0.16524	-2.5974	0.017853	1.7483
SM (OH) C22:1	5.4419	2.4441	0.020299	1.6925
Creatinine	0.11873	-3.0742	0.021572	1.6661
ADMA	0.34588	-1.5317	0.022252	1.6526
Cer(d18:2/22:0)	5.3831	2.4284	0.022997	1.6383
TG(20:2_32:1)	0.11023	-3.1813	0.023454	1.6298
PC aa C36:2	2.4167	1.273	0.024658	1.608
TG(18:2_28:0)	0.1787	-2.4844	0.026029	1.5845
DG(21:0_22:6)	0.20466	-2.2887	0.027225	1.565
HexCer(d18:1/14:0)	5.1015	2.3509	0.028045	1.5521
Cer(d18:2/24:0)	4.4999	2.1699	0.02828	1.5485
TG(14:0_34:3)	0.10413	-3.2636	0.028919	1.5388
TG(17:2_36:2)	0.091863	-3.4444	0.029276	1.5335
TG(18:1_32:2)	0.10562	-3.243	0.029748	1.5265
HipAcid	0.20025	-2.3202	0.030458	1.5163
TG(20:4_36:5)	0.097914	-3.3523	0.030706	1.5128
CE(22:1)	0.086786	-3.5264	0.030932	1.5096
TG(18:2_36:1)	0.20252	-2.3038	0.03161	1.5002
TG(16:1_34:3)	0.094529	-3.4031	0.03178	1.4979
TG(14:0_39:3)	0.092323	-3.4372	0.03377	1.4715
Cer(d18:1/20:0(OH))	0.31274	-1.677	0.035901	1.4449

Table S5

Enriched pathway	Total Cmpd	Hits	Statistic Q	Expected Q	Raw p	Holm p	FDR
Fatty acid Metabolism	2	1	99.958	20	6.70E-08	4.35E-06	1.45E-06
Mitochondrial Beta-Oxidation of Short Chain Saturated Fatty Acids	1	1	99.958	20	6.70E-08	4.35E-06	1.45E-06
Mitochondrial Beta-Oxidation of Long Chain Saturated Fatty Acids	1	1	99.958	20	6.70E-08	4.35E-06	1.45E-06
Beta Oxidation of Very Long Chain Fatty Acids	2	2	99.888	20	4.80E-07	2.98E-05	7.80E-06
Glucose-Alanine Cycle	3	3	99.478	20	7.13E-07	4.35E-05	8.77E-06
Histidine Metabolism	5	5	98.334	20	8.10E-07	4.86E-05	8.77E-06
Folate Metabolism	1	1	99.838	20	9.90E-07	5.84E-05	9.14E-06
Arachidonic Acid Metabolism	2	2	99.149	20	1.12E-06	6.52E-05	9.14E-06
Cysteine Metabolism	2	2	99.791	20	1.63E-06	9.31E-05	9.78E-06
Oxidation of Branched Chain Fatty Acids	3	3	99.495	20	1.92E-06	0.000108	9.78E-06
Glutamate Metabolism	8	8	97.945	20	2.01E-06	0.000111	9.78E-06
Taurine and Hypotaurine Metabolism	1	1	99.753	20	2.28E-06	0.000123	9.78E-06
Pantothenate and CoA Biosynthesis	1	1	99.753	20	2.28E-06	0.000123	9.78E-06
Beta-Alanine Metabolism	5	5	98.574	20	2.31E-06	0.000123	9.78E-06
Glutathione Metabolism	4	4	99.728	20	2.42E-06	0.000124	9.78E-06
Lysine Degradation	2	2	99.544	20	2.54E-06	0.000127	9.78E-06
Propanoate Metabolism	3	3	99.15	20	2.63E-06	0.000129	9.78E-06
Alanine Metabolism	3	3	99.718	20	2.71E-06	0.00013	9.78E-06
Methionine Metabolism	8	8	99.322	20	2.90E-06	0.000136	9.90E-06
Glycine and Serine Metabolism	10	10	98.843	20	3.26E-06	0.00015	9.90E-06
Porphyrin Metabolism	1	1	99.701	20	3.35E-06	0.000151	9.90E-06

Bile Acid Biosynthesis	5	1	99.701	20	3.35E-06	0.000151	9.90E-06
Arginine and Proline Metabolism	9	8	98.455	20	3.69E-06	0.000159	1.04E-05
Aspartate Metabolism	7	7	98.393	20	4.50E-06	0.000189	1.22E-05
Homocysteine Degradation	2	2	99.554	20	5.12E-06	0.00021	1.33E-05
Betaine Metabolism	3	3	99.436	20	5.69E-06	0.000228	1.37E-05
Amino Sugar Metabolism	2	2	99.358	20	5.91E-06	0.000231	1.37E-05
Nicotinate and Nicotinamide Metabolism	2	2	99.358	20	5.91E-06	0.000231	1.37E-05
Carnitine Synthesis	4	4	99.211	20	6.70E-06	0.000248	1.50E-05
Warburg Effect	4	4	98.622	20	8.01E-06	0.000288	1.74E-05
Urea Cycle	7	7	97.891	20	8.50E-06	0.000298	1.78E-05
Phenylalanine and Tyrosine Metabolism	3	3	99.227	20	9.63E-06	0.000327	1.96E-05
Purine Metabolism	5	5	99.216	20	9.99E-06	0.00033	1.97E-05
Pyrimidine Metabolism	2	2	99.016	20	1.08E-05	0.000346	2.04E-05
Phosphatidylcholine Biosynthesis	1	1	99.459	20	1.10E-05	0.000346	2.04E-05
Ammonia Recycling	7	7	99.016	20	1.26E-05	0.000379	2.28E-05
Selenoamino Acid Metabolism	2	2	99.28	20	1.32E-05	0.000384	2.28E-05
Phosphatidylethanolamine Biosynthesis	2	2	99.289	20	1.33E-05	0.000384	2.28E-05
Tryptophan Metabolism	4	4	98.773	20	1.63E-05	0.000441	2.72E-05
Phospholipid Biosynthesis	2	2	98.732	20	1.96E-05	0.00051	3.19E-05
Threonine and 2-Oxobutanoate Degradation	1	1	99.253	20	2.10E-05	0.000525	3.33E-05
Valine, Leucine and Isoleucine Degradation	5	5	98.663	20	2.57E-05	0.000616	3.97E-05
Tyrosine Metabolism	4	3	98.734	20	2.71E-05	0.000622	4.09E-05

Malate-Aspartate Shuttle	2	2	98.721	20	2.82E-05	0.000622	4.16E-05
Spermidine and Spermine Biosynthesis	5	5	96.573	20	3.81E-05	0.000801	5.51E-05
Catecholamine Biosynthesis	2	1	98.8	20	5.42E-05	0.001084	7.50E-05
Thyroid hormone synthesis	1	1	98.8	20	5.42E-05	0.001084	7.50E-05
Phenylacetate Metabolism	1	1	98.265	20	0.000114	0.002044	0.000154
Alpha Linolenic Acid and Linoleic Acid Metabolism	2	2	75.916	20	0.000236	0.004017	0.000313
Methylhistidine Metabolism	2	2	96.187	20	0.000277	0.004425	0.00036
Biotin Metabolism	1	1	96.554	20	0.000451	0.006758	0.00057
Glycolysis	1	1	96.339	20	0.000509	0.007126	0.00057
Galactose Metabolism	1	1	96.339	20	0.000509	0.007126	0.00057
Gluconeogenesis	1	1	96.339	20	0.000509	0.007126	0.00057
Lactose Synthesis	1	1	96.339	20	0.000509	0.007126	0.00057
Lactose Degradation	1	1	96.339	20	0.000509	0.007126	0.00057
Transfer of Acetyl Groups into Mitochondria	1	1	96.339	20	0.000509	0.007126	0.00057
Trehalose Degradation	1	1	96.339	20	0.000509	0.007126	0.00057
Citric Acid Cycle	1	1	96.144	20	0.000565	0.007126	0.000583
Ketone Body Metabolism	1	1	96.144	20	0.000565	0.007126	0.000583
Butyrate Metabolism	1	1	96.144	20	0.000565	0.007126	0.000583
Mitochondrial Electron Transport Chain	1	1	96.144	20	0.000565	0.007126	0.000583
Phytanic Acid Peroxisomal Oxidation	1	1	96.144	20	0.000565	0.007126	0.000583
Sphingolipid Metabolism	3	3	93.766	20	0.00059	0.007126	0.000599
Steroidogenesis	1	1	77.495	20	0.020631	0.020631	0.020631

Table S6

Pathway	Total Cmpd	Hits	Raw p	p-value (-log10)	Holm adjust	FDR	Impact
Galactose metabolism	2	2	4.04E-07	6.3939	1.82E-05	1.52E-05	0.14531
Glycerophospholipid metabolism	3	3	6.74E-07	6.1714	2.97E-05	1.52E-05	0.1377
Glycine, serine and threonine metabolism	6	6	2.39E-06	5.621	0.0001029	2.77E-05	0.52589
Glutathione metabolism	7	7	2.46E-06	5.6084	0.00010348	2.77E-05	0.1262
Sphingolipid metabolism	2	2	4.34E-06	5.3624	0.00017797	3.91E-05	0.21576
Primary bile acid biosynthesis	1	1	6.66E-06	5.1768	0.0002662	4.28E-05	0.00758
Lipoic acid metabolism	1	1	6.66E-06	5.1768	0.0002662	4.28E-05	0.0017
Arachidonic acid metabolism	2	2	7.64E-06	5.117	0.00029026	4.30E-05	0.27659
Purine metabolism	2	2	9.51E-06	5.0219	0.00035184	4.63E-05	0.01623
Porphyrin metabolism	2	2	1.10E-05	4.9601	0.00039461	4.63E-05	0
Pantothenate and CoA biosynthesis	4	4	1.13E-05	4.9461	0.00039626	4.63E-05	0.04762
beta-Alanine metabolism	5	5	1.41E-05	4.8508	0.00047938	4.67E-05	0.45522
Starch and sucrose metabolism	1	1	1.47E-05	4.8338	0.00048385	4.67E-05	0.4207
Neomycin, kanamycin and gentamicin biosynthesis	1	1	1.47E-05	4.8338	0.00048385	4.67E-05	0
One carbon pool by folate	6	6	1.56E-05	4.8079	0.00048385	4.67E-05	0.18901
Glyoxylate and dicarboxylate metabolism	4	4	1.72E-05	4.7645	0.00051598	4.84E-05	0.11
Arginine biosynthesis	6	6	2.62E-05	4.5816	0.00076001	6.51E-05	0.50532
Alanine, aspartate and glutamate metabolism	7	7	2.68E-05	4.5717	0.00076001	6.51E-05	0.621
Taurine and hypotaurine metabolism	1	1	2.90E-05	4.5383	0.00078171	6.51E-05	0
Thiamine metabolism	1	1	2.90E-05	4.5383	0.00078171	6.51E-05	0
Arginine and proline metabolism	9	8	3.47E-05	4.4596	0.00086773	7.44E-05	0.60697
Nitrogen metabolism	2	2	4.03E-05	4.3952	0.00096609	8.23E-05	0

Pyrimidine metabolism	2	2	4.65E-05	4.3325	0.0010695	9.10E-05	0
Steroid biosynthesis	1	1	4.98E-05	4.3029	0.0010952	9.33E-05	0
Propanoate metabolism	2	2	5.36E-05	4.2707	0.0011259	9.65E-05	0
Cysteine and methionine metabolism	3	3	6.01E-05	4.2209	0.0012026	0.000101	0.22222
Selenocompound metabolism	1	1	6.03E-05	4.2196	0.0012026	0.000101	0
Tyrosine metabolism	2	1	9.34E-05	4.0295	0.0016819	0.000145	0.13972
Ubiquinone and other terpenoid-quinone biosynthesis	1	1	9.34E-05	4.0295	0.0016819	0.000145	0
Linoleic acid metabolism	1	1	0.000116	3.9367	0.0018509	0.000168	0
alpha-Linolenic acid metabolism	1	1	0.000116	3.9367	0.0018509	0.000168	0
Biosynthesis of unsaturated fatty acids	3	2	0.000227	3.6431	0.0031844	0.000319	0
Histidine metabolism	4	4	0.000234	3.6303	0.0031844	0.000319	0.22131
Phenylalanine, tyrosine and tryptophan biosynthesis	2	2	0.000308	3.5114	0.0036963	0.00039	1
Phenylalanine metabolism	2	2	0.000308	3.5114	0.0036963	0.00039	0.35714
Steroid hormone biosynthesis	1	1	0.000319	3.4958	0.0036963	0.00039	0
Lysine degradation	2	2	0.000321	3.4937	0.0036963	0.00039	0
Butanoate metabolism	3	3	0.000416	3.3805	0.0036963	0.000493	0.03175
Valine, leucine and isoleucine biosynthesis	4	4	0.000532	3.2743	0.0037223	0.000614	0
Nicotinate and nicotinamide metabolism	1	1	0.000619	3.2085	0.0037223	0.000696	0
Tryptophan metabolism	2	2	0.000651	3.1865	0.0037223	0.000714	0.24798
Valine, leucine and isoleucine degradation	3	3	0.001364	2.8652	0.0054555	0.001461	0
Citrate cycle (TCA cycle)	1	1	0.006383	2.195	0.019148	0.00668	0.03273
D-Amino acid metabolism	1	1	0.036039	1.4432	0.072077	0.036858	0

Biotin metabolism	1	1	0.5747<br>2	0.2405<br>4	0.57472	0.5747<br>2	0

Table S7

Enriched pathway	Total Cmpd	Hits	Statistic Q	Expected Q	Raw p	Holm p	FDR
Tryptophan Metabolism	4	4	97.115	20	5.05E-06	0.000323	0.000111
Porphyrin Metabolism	1	1	99.605	20	5.85E-06	0.000369	0.000111
Bile Acid Biosynthesis	5	3	99.358	20	7.58E-06	0.00047	0.000111
Carnitine Synthesis	3	3	98.264	20	9.21E-06	0.000562	0.000111
Oxidation of Branched Chain Fatty Acids	3	3	91.788	20	1.36E-05	0.000818	0.000111
Beta Oxidation of Very Long Chain Fatty Acids	2	2	99.19	20	1.45E-05	0.000854	0.000111
Ammonia Recycling	7	7	97.206	20	1.46E-05	0.000854	0.000111
Phosphatidylcholine Biosynthesis	1	1	99.355	20	1.57E-05	0.000893	0.000111
Phosphatidylethanolamine Biosynthesis	2	2	98.929	20	1.59E-05	0.000893	0.000111
Phospholipid Biosynthesis	3	3	96.453	20	1.73E-05	0.000953	0.000111
Taurine and Hypotaurine Metabolism	2	2	99.096	20	1.92E-05	0.001038	0.000112
Arachidonic Acid Metabolism	2	2	95.933	20	2.66E-05	0.001411	0.000142
Methionine Metabolism	6	6	98.675	20	3.00E-05	0.001561	0.000148
Betaine Metabolism	3	3	98.66	20	3.61E-05	0.001839	0.000165
Arginine and Proline Metabolism	5	5	98.268	20	3.91E-05	0.001957	0.000167
Alanine Metabolism	3	3	98.596	20	4.37E-05	0.002139	0.000175
Phenylalanine and Tyrosine Metabolism	3	3	97.505	20	4.71E-05	0.002261	0.000177
Glutamate Metabolism	7	7	94.745	20	5.16E-05	0.002425	0.000183
Glycine and Serine Metabolism	8	8	98.158	20	5.83E-05	0.00268	0.000196
Threonine and 2-Oxobutanoate Degradation	1	1	98.718	20	6.19E-05	0.002784	0.000198
Glutathione Metabolism	4	4	98.3	20	6.72E-05	0.002958	0.000204
Valine, Leucine and Isoleucine Degradation	4	4	96.329	20	7.13E-05	0.003064	0.000204

Purine Metabolism	5	5	97.618	20	7.71E-05	0.003236	0.000204
Methylhistidine Metabolism	2	2	94.024	20	8.58E-05	0.003517	0.000204
Fatty acid Metabolism	2	2	98.338	20	0.0001025	0.004101	0.000204
Mitochondrial Beta-Oxidation of Short Chain Saturated Fatty Acids	1	1	98.347	20	0.000103	0.004101	0.000204
Mitochondrial Beta-Oxidation of Long Chain Saturated Fatty Acids	1	1	98.347	20	0.000103	0.004101	0.000204
Lysine Degradation	2	2	94.283	20	0.000103	0.004101	0.000204
Histidine Metabolism	5	5	95.95	20	0.000105	0.004101	0.000204
Steroid Biosynthesis	1	1	98.329	20	0.0001053	0.004101	0.000204
Glycerolipid Metabolism	1	1	98.329	20	0.0001053	0.004101	0.000204
Fatty Acid Elongation In Mitochondria	1	1	98.329	20	0.0001053	0.004101	0.000204
Fatty Acid Biosynthesis	1	1	98.329	20	0.0001053	0.004101	0.000204
Beta-Alanine Metabolism	5	5	95.96	20	0.0001222	0.004101	0.00023
Tyrosine Metabolism	3	3	95.957	20	0.0001649	0.004947	0.000287
Catecholamine Biosynthesis	1	1	97.894	20	0.0001674	0.004947	0.000287
Thyroid hormone synthesis	1	1	97.894	20	0.0001674	0.004947	0.000287
Glucose-Alanine Cycle	3	3	96.977	20	0.0001706	0.004947	0.000287
Warburg Effect	3	3	96.197	20	0.0002019	0.005249	0.000331
Propanoate Metabolism	3	3	96.64	20	0.0002202	0.005506	0.000352
Folate Metabolism	1	1	97.488	20	0.0002386	0.005727	0.000372
Aspartate Metabolism	5	5	96.168	20	0.0002716	0.006246	0.000408
Cysteine Metabolism	2	2	97.27	20	0.0002739	0.006246	0.000408
Homocysteine Degradation	2	2	96.609	20	0.0003026	0.006355	0.000431

Sphingolipid Metabolism	3	3	94.425	20	0.0003143	0.006355	0.000431
Pantothenate and CoA Biosynthesis	1	1	97.07	20	0.0003252	0.006355	0.000431
Glycolysis	1	1	96.93	20	0.0003572	0.00643	0.000431
Galactose Metabolism	1	1	96.93	20	0.0003572	0.00643	0.000431
Gluconeogenesis	1	1	96.93	20	0.0003572	0.00643	0.000431
Lactose Synthesis	1	1	96.93	20	0.0003572	0.00643	0.000431
Lactose Degradation	1	1	96.93	20	0.0003572	0.00643	0.000431
Transfer of Acetyl Groups into Mitochondria	1	1	96.93	20	0.0003572	0.00643	0.000431
Trehalose Degradation	1	1	96.93	20	0.0003572	0.00643	0.000431
Pyrimidine Metabolism	2	2	96.304	20	0.0004092	0.00643	0.000485
Malate-Aspartate Shuttle	2	2	95.528	20	0.0004264	0.00643	0.000488
Amino Sugar Metabolism	2	2	95.999	20	0.0004401	0.00643	0.000488
Nicotinate and Nicotinamide Metabolism	2	2	95.999	20	0.0004401	0.00643	0.000488
Selenoamino Acid Metabolism	2	2	95.795	20	0.0004423	0.00643	0.000488
Urea Cycle	4	4	95.324	20	0.0004589	0.00643	0.000498
Alpha Linolenic Acid and Linoleic Acid Metabolism	2	2	87.367	20	0.0007087	0.00643	0.000756
Spermidine and Spermine Biosynthesis	2	2	92.695	20	0.0013837	0.00643	0.001452
Steroidogenesis	1	1	93.171	20	0.0017905	0.00643	0.001848
Phenylacetate Metabolism	1	1	91.623	20	0.0027088	0.00643	0.002752
Biotin Metabolism	1	1	74.822	20	0.026108	0.026108	0.026108

Table S8

Pathway	Total Cmpd	Hits	Raw p	p-value (-log10)	Holm adjust	FDR	Impact
Biosynthesis of unsaturated fatty acids	3	3	4.01E-07	6.3966	1.89E-05	1.89E-05	0
Lipoic acid metabolism	1	1	5.85E-06	5.2326	0.00026927	9.20E-05	0.0017
Primary bile acid biosynthesis	2	2	7.76E-06	5.1101	0.00034922	9.20E-05	0.01516
Glycerophospholipid metabolism	3	3	7.83E-06	5.1063	0.00034922	9.20E-05	0.1377
Sphingolipid metabolism	2	2	1.75E-05	4.7577	0.00075127	0.00011224	0.21576
Galactose metabolism	2	2	1.87E-05	4.7287	0.00078442	0.00011224	0.14531
Taurine and hypotaurine metabolism	2	2	1.92E-05	4.7161	0.00078829	0.00011224	0.42857
Porphyrin metabolism	2	2	2.11E-05	4.6767	0.00084204	0.00011224	0
Glycine, serine and threonine metabolism	6	6	2.15E-05	4.6677	0.00084204	0.00011224	0.52589
One carbon pool by folate	6	6	3.00E-05	4.5227	0.0011405	0.00014106	0.18901
Glyoxylate and dicarboxylate metabolism	4	4	3.98E-05	4.3997	0.0014739	0.00015029	0.11
Histidine metabolism	4	4	3.99E-05	4.3996	0.0014739	0.00015029	0.22131
Arginine and proline metabolism	5	5	4.30E-05	4.3663	0.0015057	0.00015029	0.08604
Alanine, aspartate and glutamate metabolism	6	6	4.48E-05	4.349	0.0015221	0.00015029	0.621
Tryptophan metabolism	2	2	6.79E-05	4.1682	0.0022403	0.00021271	0.24798
Glutathione metabolism	4	4	7.96E-05	4.0993	0.0025457	0.00023369	0.11182
Valine, leucine and isoleucine biosynthesis	4	4	0.000101	3.9958	0.0031301	0.00023558	0
Glycosylphosphatidylinositol (GPI)-anchor biosynthesis	1	1	0.000105	3.9777	0.0031577	0.00023558	0.03947
Fatty acid biosynthesis	1	1	0.000105	3.9777	0.0031577	0.00023558	0.01473
Fatty acid elongation	1	1	0.000105	3.9777	0.0031577	0.00023558	0

Fatty acid degradation	1	1	0.000105	3.9777	0.0031577	0.00023558	0
Valine, leucine and isoleucine degradation	3	3	0.000144	3.8409	0.0037505	0.00028883	0
Purine metabolism	2	2	0.00015	3.8251	0.0037505	0.00028883	0.01623
Tyrosine metabolism	1	1	0.000167	3.7762	0.0040183	0.00028883	0.13972
Ubiquinone and other terpenoid-quinone biosynthesis	1	1	0.000167	3.7762	0.0040183	0.00028883	0
Lysine degradation	2	2	0.000169	3.7734	0.0040183	0.00028883	0
Pantothenate and CoA biosynthesis	4	4	0.000171	3.766	0.0040183	0.00028883	0.04762
Phenylalanine, tyrosine and tryptophan biosynthesis	2	2	0.000178	3.7491	0.0040183	0.00028883	1
Phenylalanine metabolism	2	2	0.000178	3.7491	0.0040183	0.00028883	0.35714
Cysteine and methionine metabolism	3	3	0.000263	3.5797	0.0047379	0.00041237	0.22222
beta-Alanine metabolism	4	4	0.000282	3.55	0.0047912	0.0004273	0.45522
Thiamine metabolism	1	1	0.000325	3.4879	0.0052026	0.00047758	0
Starch and sucrose metabolism	1	1	0.000357	3.4471	0.0053582	0.00048488	0.4207
Neomycin, kanamycin and gentamicin biosynthesis	1	1	0.000357	3.4471	0.0053582	0.00048488	0
Arachidonic acid metabolism	2	2	0.000361	3.4424	0.0053582	0.00048488	0.27659
Pyrimidine metabolism	2	2	0.000409	3.3881	0.0053582	0.00053417	0
Propanoate metabolism	1	1	0.00044	3.3568	0.0053582	0.00054439	0
Nitrogen metabolism	2	2	0.00044	3.3564	0.0053582	0.00054439	0
Arginine biosynthesis	3	3	0.000503	3.2982	0.0053582	0.00060466	0.12234
Selenocompound metabolism	1	1	0.000515	3.2885	0.0053582	0.00060466	0
Linoleic acid metabolism	1	1	0.000846	3.0727	0.0059213	0.00094661	0
alpha-Linolenic acid metabolism	1	1	0.000846	3.0727	0.0059213	0.00094661	0

Nicotinate and nicotinamide metabolism	1	1	0.001268	2.8969	0.0063402	0.001386	0
Butanoate metabolism	2	2	0.001344	2.8715	0.0063402	0.0014358	0.03175
Steroid hormone biosynthesis	1	1	0.001791	2.747	0.0063402	0.00187	0
D-Amino acid metabolism	1	1	0.001927	2.7151	0.0063402	0.0019691	0
Biotin metabolism	1	1	0.026108	1.5832	0.026108	0.026108	0

Table S9

Enriched pathway	Total Cmpd	Hits	Statistic Q	Expected Q	Raw p	Holm p	FDR
Taurine and Hypotaurine Metabolism	2	2	99.86	20	9.32E-07	6.43E-05	1.53E-05
Pantothenate and CoA Biosynthesis	1	1	99.824	20	1.16E-06	7.88E-05	1.53E-05
Porphyrin Metabolism	1	1	99.779	20	1.83E-06	0.000123	1.53E-05
Beta Oxidation of Very Long Chain Fatty Acids	2	2	99.755	20	1.83E-06	0.000123	1.53E-05
Glutamate Metabolism	8	8	98.55	20	1.95E-06	0.000127	1.53E-05
Bile Acid Biosynthesis	5	5	99.392	20	2.11E-06	0.000135	1.53E-05
Glutathione Metabolism	4	4	99.706	20	3.05E-06	0.000192	1.53E-05
Ammonia Recycling	7	7	99.539	20	3.13E-06	0.000194	1.53E-05
Homocysteine Degradation	2	2	99.664	20	3.40E-06	0.000208	1.53E-05
Cysteine Metabolism	2	2	99.69	20	3.53E-06	0.000212	1.53E-05
Methionine Metabolism	8	8	99.632	20	3.55E-06	0.000212	1.53E-05
Glycine and Serine Metabolism	10	10	99.619	20	3.89E-06	0.000225	1.53E-05
Purine Metabolism	6	6	99.498	20	3.95E-06	0.000225	1.53E-05
Alanine Metabolism	3	3	99.664	20	3.98E-06	0.000225	1.53E-05
Phosphatidylcholine Biosynthesis	1	1	99.659	20	4.37E-06	0.00024	1.53E-05
Aspartate Metabolism	7	6	99.476	20	4.50E-06	0.000243	1.53E-05

Betaine Metabolism	3	3	99.572	20	4.53E-06	0.000243	1.53E-05
Arginine and Proline Metabolism	9	9	99.529	20	4.82E-06	0.000251	1.53E-05
Carnitine Synthesis	4	4	99.498	20	4.87E-06	0.000251	1.53E-05
Steroid Biosynthesis	1	1	99.603	20	5.93E-06	0.000296	1.53E-05
Glycerolipid Metabolism	1	1	99.603	20	5.93E-06	0.000296	1.53E-05
Fatty Acid Elongation In Mitochondria	1	1	99.603	20	5.93E-06	0.000296	1.53E-05
Threonine and 2-Oxobutanoate Degradation	1	1	99.594	20	6.19E-06	0.000296	1.53E-05
Urea Cycle	7	7	99.445	20	6.39E-06	0.000296	1.53E-05
Fatty acid Metabolism	2	2	99.575	20	6.60E-06	0.000297	1.53E-05
Beta-Alanine Metabolism	6	5	99.172	20	6.64E-06	0.000297	1.53E-05
Phosphatidylethanolamine Biosynthesis	2	2	99.512	20	6.77E-06	0.000297	1.53E-05
Mitochondrial Beta-Oxidation of Short Chain Saturated Fatty Acids	1	1	99.545	20	7.76E-06	0.000326	1.53E-05
Mitochondrial Beta-Oxidation of Long Chain Saturated Fatty Acids	1	1	99.545	20	7.76E-06	0.000326	1.53E-05
Arachidonic Acid Metabolism	2	2	99.467	20	7.79E-06	0.000326	1.53E-05
Malate-Aspartate Shuttle	2	2	99.312	20	7.80E-06	0.000326	1.53E-05

Histidine Metabolism	6	5	98.992	20	7.86E-06	0.000326	1.53E-05
Folate Metabolism	1	1	99.539	20	8.00E-06	0.000326	1.53E-05
Spermidine and Spermine Biosynthesis	5	5	99.515	20	8.01E-06	0.000326	1.53E-05
Tryptophan Metabolism	4	4	99.361	20	8.40E-06	0.000326	1.53E-05
Propanoate Metabolism	3	2	99.482	20	8.53E-06	0.000326	1.53E-05
Amino Sugar Metabolism	2	2	99.471	20	8.76E-06	0.000326	1.53E-05
Nicotinate and Nicotinamide Metabolism	2	2	99.471	20	8.76E-06	0.000326	1.53E-05
Tyrosine Metabolism	4	3	99.299	20	9.00E-06	0.000326	1.53E-05
Selenoamino Acid Metabolism	2	2	99.492	20	9.00E-06	0.000326	1.53E-05
Valine, Leucine and Isoleucine Degradation	5	5	99.393	20	9.21E-06	0.000326	1.53E-05
Glucose-Alanine Cycle	3	3	99.471	20	9.29E-06	0.000326	1.53E-05
Phenylalanine and Tyrosine Metabolism	3	3	99.442	20	9.82E-06	0.000326	1.58E-05
Methylhistidine Metabolism	2	2	98.958	20	1.09E-05	0.000326	1.70E-05
Warburg Effect	4	4	99.36	20	1.13E-05	0.000326	1.73E-05
Phospholipid Biosynthesis	3	3	98.769	20	1.18E-05	0.000326	1.77E-05

Pyrimidine Metabolism	2	1	99.38	20	1.44E-05	0.000332	2.08E-05
Phenylacetate Metabolism	1	1	99.38	20	1.44E-05	0.000332	2.08E-05
Lysine Degradation	3	2	99.318	20	1.52E-05	0.000332	2.14E-05
Citric Acid Cycle	1	1	99.328	20	1.70E-05	0.000339	2.17E-05
Ketone Body Metabolism	1	1	99.328	20	1.70E-05	0.000339	2.17E-05
Butyrate Metabolism	1	1	99.328	20	1.70E-05	0.000339	2.17E-05
Mitochondrial Electron Transport Chain	1	1	99.328	20	1.70E-05	0.000339	2.17E-05
Phytanic Acid Peroxisomal Oxidation	1	1	99.328	20	1.70E-05	0.000339	2.17E-05
Catecholamine Biosynthesis	2	1	99.259	20	2.06E-05	0.000339	2.54E-05
Thyroid hormone synthesis	1	1	99.259	20	2.06E-05	0.000339	2.54E-05
Oxidation of Branched Chain Fatty Acids	4	4	97.276	20	2.45E-05	0.000339	2.96E-05
Biotin Metabolism	1	1	98.874	20	4.77E-05	0.000572	5.67E-05
Glycolysis	1	1	98.715	20	6.22E-05	0.000684	6.60E-05
Galactose Metabolism	1	1	98.715	20	6.22E-05	0.000684	6.60E-05
Gluconeogenesis	1	1	98.715	20	6.22E-05	0.000684	6.60E-05

Lactose Synthesis	1	1	98.715	20	6.22E-05	0.000684	6.60E-05
Lactose Degradation	1	1	98.715	20	6.22E-05	0.000684	6.60E-05
Transfer of Acetyl Groups into Mitochondria	1	1	98.715	20	6.22E-05	0.000684	6.60E-05
Trehalose Degradation	1	1	98.715	20	6.22E-05	0.000684	6.60E-05
Fatty Acid Biosynthesis	2	2	93.585	20	0.00024696	0.000988	0.000258
Sphingolipid Metabolism	3	3	94.065	20	0.00083581	0.002507	0.000861
Steroidogenesis	1	1	94.279	20	0.0012517	0.002507	0.00127
Alpha Linolenic Acid and Linoleic Acid Metabolism	1	1	89.968	20	0.0039076	0.003908	0.003908

Table S10

Pathway	Total Cmpd	Hits	Raw p	p-value (-log10)	Holm adjust	FDR	Impact
Linoleic acid metabolism	1	1	1.16E-07	6.9352	5.57E-06	2.79E-06	0
alpha-Linolenic acid metabolism	1	1	1.16E-07	6.9352	5.57E-06	2.79E-06	0
Arachidonic acid metabolism	2	2	2.61E-07	6.5832	1.20E-05	4.18E-06	0.27659
Glycerophospholipid metabolism	3	3	7.51E-07	6.1246	3.38E-05	8.94E-06	0.1377
Taurine and hypotaurine metabolism	2	2	9.32E-07	6.0308	4.10E-05	8.94E-06	0.42857
Thiamine metabolism	1	1	1.16E-06	5.9363	4.98E-05	9.26E-06	0
Primary bile acid biosynthesis	3	3	1.54E-06	5.8119	6.48E-05	1.06E-05	0.02493
Lipoic acid metabolism	1	1	1.83E-06	5.7376	7.50E-05	1.10E-05	0.0017
Glycine, serine and threonine metabolism	6	6	2.93E-06	5.5337	0.000117	1.27E-05	0.52589
One carbon pool by folate	6	6	3.22E-06	5.4918	0.000126	1.27E-05	0.18901
Porphyrin metabolism	2	2	3.58E-06	5.4466	0.000136	1.27E-05	0
Glutathione metabolism	7	7	3.98E-06	5.4002	0.000147	1.27E-05	0.1262
beta-Alanine metabolism	6	5	4.23E-06	5.3739	0.000152	1.27E-05	0.11194
Pantothenate and CoA biosynthesis	4	3	4.83E-06	5.3164	0.000169	1.27E-05	0
Cysteine and methionine metabolism	3	3	4.99E-06	5.3018	0.00017	1.27E-05	0.22222
Selenocompound metabolism	1	1	5.02E-06	5.2993	0.00017	1.27E-05	0
Purine metabolism	3	3	5.40E-06	5.2677	0.000173	1.27E-05	0.02769
Biosynthesis of unsaturated fatty acids	2	2	5.61E-06	5.2513	0.000174	1.27E-05	0
Glyoxylate and dicarboxylate metabolism	4	4	5.68E-06	5.2454	0.000174	1.27E-05	0.11
Glycosylphosphatidylinositol (GPI)-anchor biosynthesis	1	1	5.93E-06	5.2272	0.000174	1.27E-05	0.03947
Fatty acid elongation	1	1	5.93E-06	5.2272	0.000174	1.27E-05	0

Fatty acid degradation	1	1	5.93E-06	5.2272	0.000174	1.27E-05	0
Histidine metabolism	5	5	6.07E-06	5.2167	0.000174	1.27E-05	0.31147
Arginine biosynthesis	6	6	7.13E-06	5.1469	0.000178	1.43E-05	0.50532
Nitrogen metabolism	2	2	8.76E-06	5.0575	0.00021	1.68E-05	0
Galactose metabolism	2	2	9.30E-06	5.0313	0.000214	1.72E-05	0.14531
Valine, leucine and isoleucine biosynthesis	4	4	9.74E-06	5.0113	0.000214	1.73E-05	0
Valine, leucine and isoleucine degradation	3	3	1.15E-05	4.939	0.000242	1.97E-05	0
Phenylalanine, tyrosine and tryptophan biosynthesis	2	2	1.33E-05	4.8748	0.000267	2.13E-05	1
Phenylalanine metabolism	2	2	1.33E-05	4.8748	0.000267	2.13E-05	0.35714
Lysine degradation	3	2	1.40E-05	4.8548	0.000267	2.16E-05	0
Pyrimidine metabolism	2	1	1.44E-05	4.8405	0.000267	2.17E-05	0
Arginine and proline metabolism	9	9	1.53E-05	4.8145	0.000267	2.23E-05	0.6279
Citrate cycle (TCA cycle)	1	1	1.70E-05	4.7704	0.000267	2.33E-05	0.03273
Propanoate metabolism	2	1	1.70E-05	4.7704	0.000267	2.33E-05	0
Tyrosine metabolism	2	1	2.06E-05	4.6853	0.000268	2.68E-05	0.13972
Ubiquinone and other terpenoid-quinone biosynthesis	1	1	2.06E-05	4.6853	0.000268	2.68E-05	0
Alanine, aspartate and glutamate metabolism	7	7	2.14E-05	4.6706	0.000268	2.70E-05	0.621
Tryptophan metabolism	2	2	2.28E-05	4.6422	0.000268	2.81E-05	0.24798
D-Amino acid metabolism	1	1	2.71E-05	4.5663	0.000268	3.26E-05	0
Nicotinate and nicotinamide metabolism	1	1	3.04E-05	4.5169	0.000268	3.56E-05	0
Sphingolipid metabolism	2	2	3.70E-05	4.4315	0.000268	4.23E-05	0.21576
Biotin metabolism	1	1	4.77E-05	4.3215	0.000286	5.32E-05	0
Starch and sucrose metabolism	1	1	6.22E-05	4.2064	0.000311	6.63E-05	0.4207

Neomycin, kanamycin and gentamicin biosynthesis	1	1	6.22E-05	4.2064	0.000311	6.63E-05	0
Butanoate metabolism	3	3	0.00013	3.8859	0.00039	0.000136	0.03175
Fatty acid biosynthesis	2	2	0.000247	3.6074	0.000494	0.000252	0.01473
Steroid hormone biosynthesis	1	1	0.001252	2.9025	0.001252	0.001252	0

Table S11
